# ESTREL-Fatigue—association of levodopa with post-stroke fatigue

**DOI:** 10.1093/esj/aakag029

**Published:** 2026-04-07

**Authors:** Lukas Boos, Josefin E Kaufmann, Mirjam I Sauter, Philippe Lyrer, Annaelle Zietz, Simon Trüssel, Sophia V Engelter, Andreas R Luft, Alexandros Polymeris, Valerian L Altersberger, Karin Wiesner, Jeremia P O Held, Yannik Rottenberger, Anne Schwarz, Friedrich Medlin, Ettore A Accolla, Sandrine Foucras, Georg Kägi, Gian Marco De Marchis, Svetlana Politz, Matthias Greulich, Alexander A Tarnutzer, Rolf Sturzenegger, Mira Katan, Urs Fischer, Krassen Nedeltchev, Janine Schär, Katrien Van Den Keybus Deglon, Pierre-André Rapin, Alexander Salerno, David Seiffge, Elias Auer, Julian Lippert, Leo H Bonati, Corina Schuster-Amft, Szabina Gäumann, Joelle N Chabwine, Andrea M Humm, Jens Carsten Möller, Raoul Schweinfurther, Bartosz Bujan, Piotr Jedrysiak, Peter S Sandor, Roman Gonzenbach, Mylius Veit, Dietmar Lutz, Carmen Lienert, Nils Peters, Davide Strambo, René M Müri, Sabine Schädelin, Lars G Hemkens, Gary A Ford, Anna Kuppuswamy, Anners Lerdal, Dawn B Simpson, Coralie English, Stewart Blackwell, Henrik Gensicke, Christopher Traenka, Stefan T Engelter

**Affiliations:** Departement of Rehabilitation and Neurology, University Department of Geriatric Medicine FELIX PLATTER, University of Basel, Basel, Switzerland; Department of Neurology and Stroke Center, Department of Clinical Research, University Hospital Basel and University of Basel, Basel, Switzerland; Departement of Rehabilitation and Neurology, University Department of Geriatric Medicine FELIX PLATTER, University of Basel, Basel, Switzerland; Department of Neurology and Stroke Center, Department of Clinical Research, University Hospital Basel and University of Basel, Basel, Switzerland; Departement of Rehabilitation and Neurology, University Department of Geriatric Medicine FELIX PLATTER, University of Basel, Basel, Switzerland; Department of Neurology and Stroke Center, Department of Clinical Research, University Hospital Basel and University of Basel, Basel, Switzerland; Departement of Rehabilitation and Neurology, University Department of Geriatric Medicine FELIX PLATTER, University of Basel, Basel, Switzerland; Departement of Rehabilitation and Neurology, University Department of Geriatric Medicine FELIX PLATTER, University of Basel, Basel, Switzerland; Department of Neurology and Stroke Center, Department of Clinical Research, University Hospital Basel and University of Basel, Basel, Switzerland; Departement of Rehabilitation and Neurology, University Department of Geriatric Medicine FELIX PLATTER, University of Basel, Basel, Switzerland; Department of Neurology and Stroke Center, Department of Clinical Research, University Hospital Basel and University of Basel, Basel, Switzerland; Departement of Rehabilitation and Neurology, University Department of Geriatric Medicine FELIX PLATTER, University of Basel, Basel, Switzerland; Department of Neurology and Stroke Center, Department of Clinical Research, University Hospital Basel and University of Basel, Basel, Switzerland; Division of Vascular Neurology and Neurorehabilitation, Department of Neurology, University Hospital of Zurich and University of Zurich, Zurich, Switzerland; Cereneo, Center for Neurology and Rehabilitation, Vitznau, Switzerland; Department of Neurology and Stroke Center, Department of Clinical Research, University Hospital Basel and University of Basel, Basel, Switzerland; Stroke Division, Department of Neurology, Beth Israel Deaconess Medical Center, Harvard Medical School, Boston, MA, United States; Department of Neurology and Stroke Center, Department of Clinical Research, University Hospital Basel and University of Basel, Basel, Switzerland; Department of Medicine and Neurology, Melbourne Brain Centre at the Royal Melbourne Hospital, University of Melbourne, Parkville, Victoria, Australia; Departement of Rehabilitation and Neurology, University Department of Geriatric Medicine FELIX PLATTER, University of Basel, Basel, Switzerland; Division of Vascular Neurology and Neurorehabilitation, Department of Neurology, University Hospital of Zurich and University of Zurich, Zurich, Switzerland; Bellevue Medical Group, Zürich, Switzerland; Division of Vascular Neurology and Neurorehabilitation, Department of Neurology, University Hospital of Zurich and University of Zurich, Zurich, Switzerland; Division of Vascular Neurology and Neurorehabilitation, Department of Neurology, University Hospital of Zurich and University of Zurich, Zurich, Switzerland; Department of Neurology, David Geffen School of Medicine at UCLA, Los Angeles, CA, United States; Service of Neurology and Stroke Unit, HFR Fribourg–Cantonal Hospital, Fribourg, Switzerland; Service of Neurology and Stroke Unit, HFR Fribourg–Cantonal Hospital, Fribourg, Switzerland; Service of Neurology and Stroke Unit, HFR Fribourg–Cantonal Hospital, Fribourg, Switzerland; Department of Neurology, Kantonsspital St. Gallen, St. Gallen, Switzerland; Department of Neurology, Inselspital Bern, University Hospital and University of Bern, Bern, Switzerland; Neurology Department & Stroke Center, HOCH-Kantonsspital St. Gallen, University Teaching and Research Hospital, St. Gallen, Switzerland; Kantonsspital Münsterlingen, Münsterlingen, Thurgau, Switzerland; Kantonsspital Winterthur, Winterthur, Zurich, Switzerland; Neuropraxis Zürich GmbH, Zurich, Switzerland; Neurology, Cantonal Hospital of Baden, Baden, Switzerland; Faculty of Medicine, University of Zurich, Zurich, Switzerland; Departement Innere Medizin, Kantonsspital Graubünden, Neurologie, Chur, Switzerland; Department of Neurology and Stroke Center, Department of Clinical Research, University Hospital Basel and University of Basel, Basel, Switzerland; Department of Neurology, Inselspital Bern, University Hospital and University of Bern, Bern, Switzerland; Department of Neurology, Inselspital Bern, University Hospital and University of Bern, Bern, Switzerland; Department of Neurology, Cantonal Hospital of Aarau, Aarau, Switzerland; Department of Neurology and Stroke Center, Klinik Hirslanden, Zürich, Switzerland; Acute Neurorehabilitation Unit, Department of Clinical Neurosciences, Lausanne University Hospital and University of Lausanne, Lausanne, Switzerland; Lavigny Rehabilitation Institution, Lavigny, Switzerland; Stroke Center, Service of Neurology, Department of Clinical Neurosciences, Lausanne University Hospital and University of Lausanne, Lausanne, Switzerland; Department of Neurology, Inselspital Bern, University Hospital and University of Bern, Bern, Switzerland; Department of Neurology, Inselspital Bern, University Hospital and University of Bern, Bern, Switzerland; Graduate School of Health Science, University of Bern, Bern, Switzerland; Department of Neurology, Inselspital Bern, University Hospital and University of Bern, Bern, Switzerland; Department of Neurology and Stroke Center, Department of Clinical Research, University Hospital Basel and University of Basel, Basel, Switzerland; Research Department, Reha Rheinfelden, Rheinfelden 4310, Switzerland; Research Department, Reha Rheinfelden, Rheinfelden 4310, Switzerland; School of Engineering and Computer Science, Bern University of Applied Sciences, Biel, Switzerland; Department for Sport, Exercise and Health, University of Basel, Basel, Switzerland; Research Department, Reha Rheinfelden, Rheinfelden 4310, Switzerland; Division of Neurorehabilitation, Fribourg Hospital, Meyriez-Murten, Switzerland; Laboratory for Neurorehabilitation Science, Medicine Section, University of Fribourg, Fribourg, Switzerland; Service of Neurology and Stroke Unit, HFR Fribourg–Cantonal Hospital, Fribourg, Switzerland; Division of Neurorehabilitation, Fribourg Hospital, Meyriez-Murten, Switzerland; Rehaklinik Zihlschlacht AG, Zihlschlacht, Switzerland; Department of Neurology, Philipps University, Marburg, Germany; Rehaklinik Zihlschlacht AG, Zihlschlacht, Switzerland; Neurorehabilitation, Klinik Lengg Zürich, Zurich, Switzerland; Neurorehabilitation, Klinik Lengg Zürich, Zurich, Switzerland; Research Department, Rehaklinik Bad Zurzach ZURZACH Care Group, Zurzach, Switzerland; Kliniken Valens Pfäfers, St. Gallen, Switzerland; Department of Neurology, Philipps University, Marburg, Germany; Department of Neurology, Center for Neurorehabilitation, Valens, Switzerland; Department of Neurology, Kantonsspital Graubünden, Chur, Switzerland; Kliniken Valens Pfäfers, St. Gallen, Switzerland; Kliniken Valens Pfäfers, St. Gallen, Switzerland; Department of Neurology and Stroke Center, Klinik Hirslanden, Zürich, Switzerland; Stroke Center, Service of Neurology, Department of Clinical Neurosciences, Lausanne University Hospital and University of Lausanne, Lausanne, Switzerland; Department of Neurology, Inselspital Bern, University Hospital and University of Bern, Bern, Switzerland; Research Department, Rehaklinik Bad Zurzach ZURZACH Care Group, Zurzach, Switzerland; Gerontechnology and Rehabilitation Group, University of Bern, Bern, Switzerland; Department Clinical Research, University Hospital Basel, Basel, Switzerland; Department of Clinical Research, University of Bern, Bern, Switzerland; Pragmatic Evidence Lab, Research Center for Clinical Neuroimmunology and Neuroscience Basel (RC2NB), University Hospital Basel and University of Basel, Basel, Switzerland; Meta-Research Innovation Center at Stanford (METRICS), Stanford University, Stanford, CA, United States; Radcliffe Department of Medicine, University of Oxford, Oxford, United Kingdom; School of Biomedical Sciences, Faculty of Biological Sciences, University of Leeds, Leeds, United Kingdom; Department of Public Health and Interdisciplinary Health Science, Institute of Health and Society, Faculty of Medicine, University of Oslo, Oslo, Norway; Research Department, Lovisenberg Diaconal Hospital, Oslo, Norway; School of Health Sciences, College of Health, Medicine and Wellbeing, University of Newcastle, Newcastle, Australia; Heart and Stroke Program, Hunter Medical Research Institute, Newcastle, Australia; School of Health Sciences, College of Health, Medicine and Wellbeing, University of Newcastle, Newcastle, Australia; Heart and Stroke Program, Hunter Medical Research Institute, Newcastle, Australia; Centre of Research Excellence to Accelerate Stroke Trial Innovation and Translation, University of Sydney, Sydney, Level 5, Block K, Westmead Hospital, Australia; School of Health Sciences, College of Health, Medicine and Wellbeing, University of Newcastle, Newcastle, Australia; Heart and Stroke Program, Hunter Medical Research Institute, Newcastle, Australia; Departement of Rehabilitation and Neurology, University Department of Geriatric Medicine FELIX PLATTER, University of Basel, Basel, Switzerland; Department of Neurology and Stroke Center, Department of Clinical Research, University Hospital Basel and University of Basel, Basel, Switzerland; Department of Clinical Research, University of Bern, Bern, Switzerland; Departement of Rehabilitation and Neurology, University Department of Geriatric Medicine FELIX PLATTER, University of Basel, Basel, Switzerland; Department of Neurology and Stroke Center, Department of Clinical Research, University Hospital Basel and University of Basel, Basel, Switzerland; Departement of Rehabilitation and Neurology, University Department of Geriatric Medicine FELIX PLATTER, University of Basel, Basel, Switzerland; Department of Neurology and Stroke Center, Department of Clinical Research, University Hospital Basel and University of Basel, Basel, Switzerland

**Keywords:** fatigue, levodopa, post-stroke fatigue, rehabilitation, stroke

## Abstract

**Introduction:**

Post-stroke fatigue (PSF) is common and impacts stroke rehabilitation. Dopaminergic treatment may have beneficial effects on PSF. This study investigated whether levodopa, compared with placebo, was associated with a lower frequency or severity of PSF during in-hospital rehabilitation.

**Patients and methods:**

Enhancement of Stroke Rehabilitation with Levodopa (ESTREL)-Fatigue was an exploratory analysis of secondary outcome data obtained in the multicentre, randomised, placebo-controlled ESTREL trial. Participants with acute stroke received levodopa 100 mg/carbidopa 25 mg or placebo 3 times daily for 39 days to enhance motor recovery. Participants who (i) reported fatigue at 5 weeks and who (ii) took at least 80% of the study medication were included in ESTREL-Fatigue. No adjustments for confounding were made. The primary endpoint was the presence of PSF at 5 weeks, defined as a T-score of ≥ 55 on the Patient-Reported Outcomes Measurement Information System (PROMIS) Fatigue-Short-form-4a. As secondary endpoints, T-score cutoffs of ≥ 60 (moderate fatigue) and ≥ 70 (severe fatigue) were used. Binary logistic regression was used to compare PSF at 5 weeks between treatment groups. Results are presented as odds ratios (ORs) with 95% CI.

**Results:**

A total of 456 of 505 (90.3%) participants were included (levodopa/placebo 235/221, median age 73 years, 41% female). Post-stroke fatigue at 5 weeks was present in 63/235 (26.8%) levodopa-treated participants and in 65/221 (29.4%) placebo-treated participants (OR: 0.88; 95% CI, 0.58–1.32; risk ratio 0.91; risk difference − 2.6%). For cutoffs of ≥ 60 and ≥ 70, ORs were 0.78 (95% CI, 0.43–1.41) and 0.8 (95% CI, 0.25–2.44), respectively. A sensitivity analysis as per intention-to-treat with all 610 randomised ESTREL participants also showed no significant difference in fatigue presence between levodopa and placebo groups (OR: 0.95; 95% CI, 0.65–1.39) and a sensitivity analysis using a mixed-effects logistic regression showed no evidence of centre-related clustering.

**Conclusion:**

In ESTREL-Fatigue, levodopa, compared to placebo, was not associated with less PSF during in-hospital rehabilitation.

## Graphical Abstract

**Graphical Abstract:**
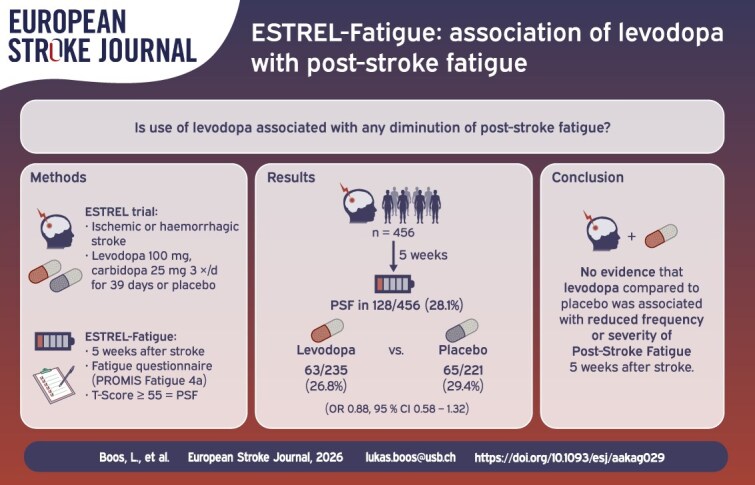

## Introduction

Post-stroke fatigue (PSF) is one of the most debilitating and distressing symptoms after stroke.^1^ Post-stroke fatigue is defined as a feeling of exhaustion (rather than tiredness, motor weakness or lack of motivation), that can involve physical, emotional, cognitive or perceptual aspects, has an impact on daily activities or routines and is not relieved by rest.^2–5^ A recent systematic review from 2023 reports a global PSF prevalence of 46.79% in stroke survivors.^6^ Post-stroke fatigue is associated with increased mortality^7,8^ and reduced quality of life,^9^ highlighting its significant burden on stroke survivors.

An established neuroimmune model of PSF proposes that inflammation in brain tissue after stroke may result in metabolic changes,^10,11^ including reduced synthesis of monoamine neurotransmitters such as dopamine.^12,13^ These immune-mediated changes after stroke may contribute to PSF or post-stroke depression.^14,15^ Dopamine, a behaviourally powerful neuromodulator, can affect motor function, reward processing and motivational behaviour.^16^ Imaging studies further suggest an association between impaired dopaminergic pathways, decreased cortical activation and the ability to maintain focused attention.^17–19^ Consequently, strategies to increase dopamine concentration have been identified as a promising target in the treatment^5^ and prevention of PSF.

There is limited evidence about the clinical efficacy of interventions for the prevention and treatment of PSF.^20^ Currently, there is no evidence that PSF can be prevented using existing interventions. A range of non-pharmacological interventions has been reported to improve PSF, though evidence quality is low.^21^ For pharmacological interventions, a recent systematic review concluded that there is insufficient evidence to support a specific pharmacological treatment for PSF.^22^ However, a small randomised controlled trial (*n* = 36^23^) targeting dopaminergic pathways with modafinil, reported reduced PSF and improved quality of life.

With these considerations in mind, we assessed whether use of levodopa during inpatient rehabilitation was associated with reduced (i) frequency and (ii) severity of PSF, compared with those who received a placebo in the randomised controlled Enhancement of Stroke Rehabilitation with Levodopa (ESTREL) trial.

## Patients and methods

### Study design and study population

Enhancement of Stroke Rehabilitation with Levodopa-Fatigue was an exploratory analysis of data from the ESTREL trial (NCT03735901^24^). Enhancement of Stroke Rehabilitation with Levodopa was a multicentre, randomised, parallel-group, double-masked, placebo-controlled trial conducted across 13 acute stroke centres and units and 11 rehabilitation centres in Switzerland. The aim of the ESTREL trial was to investigate whether levodopa, compared with placebo administered during in-hospital rehabilitation, could enhance motor recovery in patients after acute stroke. Participants were randomly assigned in a 1:1 ratio to receive either levodopa/carbidopa (100/25 mg) 3 times daily or a matching placebo for 5 weeks (39 days, including dose escalation and tapering). The primary endpoint of ESTREL was the Fugl–Meyer Motor Assessment (FMMA) at 3 months.^25^ The main results of ESTREL have been published,^26^ showing no significant evidence that levodopa enhances motor recovery when added to standardised rehabilitation therapy.

Enhancement of Stroke Rehabilitation with Levodopa-Fatigue utilised data prospectively obtained as secondary outcomes. The approach to analysing fatigue was mentioned in the ESTREL study protocol (Supplementary Material “Study protocol ESTREL,” p. 22), which stated that exploratory analyses would be performed (Supplementary Material “Study protocol ESTREL,” p. 39). The ESTREL statistical analysis plan stated that secondary outcomes were to be analysed using logistic regression models (Supplementary Material “SAP ESTREL,” p. 10), without mentioning the analyses of fatigue explicitly. This analysis should therefore be interpreted as post hoc. In detail, all ESTREL participants were asked to complete the Patient-Reported Outcomes Measurement Information System (PROMIS) form 29 at 5 weeks after randomisation, which contains self-reported information about fatigue. All ESTREL participants who (i) provided information about the presence or absence of fatigue and (ii) took at least 80% of the study medication (levodopa or placebo) were eligible for this sub-study. Participants with incomplete data regarding fatigue in PROMIS were excluded.

We investigated PSF at 5 weeks post-stroke because this corresponded to (i) the end of the levodopa treatment and (ii) the predefined timing of assessments in the ESTREL trial.

### Participant characteristics

Participant variables considered for subgroup and exploratory analysis included age, sex, stroke type (ischaemic or haemorrhagic), affected arterial territory, use of acute recanalisation therapy, previous stroke, pre-stroke mRS, stroke severity measures at randomisation (mRS, FMMA and NIHSS), aphasia (NIHSS item aphasia) and depression status at baseline and 5 weeks.

### Primary endpoint

The primary endpoint of ESTREL-Fatigue was presence of PSF at 5 weeks after randomisation. Presence of PSF was determined by the PROMIS Short-form v1.0—Fatigue 4a (PROMIS Fatigue SF-4a) component of PROMIS-29 v2.1, a large item bank that enables use of domain-specific short forms.^27^ The PROMIS Fatigue SF-4a consists of 4 fatigue items rated on a 5-point scale, where higher scores indicate more fatigue. A T-score of 50 on the PROMIS Fatigue SF-4a represents the average level of fatigue in the pooled United States general population, with a standard deviation of ± 10.

For ESTREL-Fatigue, PSF presence was defined as participants with a T-score of 55 or larger. As there was no established cut-off score for PSF or a stroke-specific cross-validation for this measure, we referred to the general population, where a T-score of ≥ 55 has been reported to detect fatigue as the most sensitive cutoff. This cutoff was based on recommendations from the US National Institutes of Health.^28^

### Secondary endpoints

Primary analysis of Fatigue SF-4a T-scores was repeated using higher cutoffs of ≥ 60 and ≥ 70. Fatigue severity categories were then defined as mild (55–59), moderate (60–69) and severe (≥70), according to categories from the PROMIS Fatigue Item Bank.^29^ Fatigue severity distribution was then compared between treatment groups. Lastly, fatigue was examined using the single fatigue item from the PROMIS-10 form,^30^ which asks participants to rate their fatigue on a scale from “none” to “very severe.” To assess treatment effect agreement between PROMIS SF-4a and PROMIS-10 scores, all cutoffs were evaluated to dichotomise into fatigue and no fatigue.

Two further exploratory subgroup analyses were performed: (i) interaction of levodopa treatment and participant characteristics, and (ii) association of fatigue with these characteristics regardless of treatment. For both analyses, variables analysed included age, sex (male vs female), type of stroke (ischaemic vs haemorrhagic), affected vessel (middle vs anterior, vs posterior vs vertebrobasilar artery), acute recanalisation therapy (yes vs no), previous stroke (yes vs no), depression at baseline (yes vs no), aphasia at baseline (yes vs no) and stroke impairment (mRS, NIHSS, FMMA). Age, NIHSS scores and FMMA scores were dichotomised using their respective medians; mRS was dichotomised low (0–2) and high (3–5).

Sensitivity analysis included recalculation of the primary endpoint using a mixed-effects logistic regression with a random intercept to look for centre-related clustering. Intention-to-treat analysis was performed including all randomised ESTREL participants, irrespective of medication adherence and compared fatigue presence between groups. Medication allocation in non-adherent participants excluded from ESTREL-Fatigue was also analysed.

### Statistical methods

Baseline characteristics were summarised descriptively by the treatment group. Standardised mean differences (SMDs) were calculated to quantify the magnitude of between-group differences for categorical and continuous variables.

T-scores for PROMIS Fatigue SF-4a were calculated using the online scoring service provided by the US assessment centre,^31^ using response pattern scoring for each participant. Raw scores were also converted into T-scores by using the analogue conversion table provided by the National Institutes of Health in the United States.^28^ Due to the higher accuracy of the response pattern scoring, these T-scores were used for subsequent analyses.

For the primary (cutoff T-score of ≥ 55) and secondary endpoints (cutoff T-score of 60 and 70), logistic regression analyses were performed with fatigue as the dependent variable and the treatment group (levodopa vs placebo) as the independent variable. Results were expressed as odds ratios (ORs) with their respective 95% CI and visualised using forest plots.

Fatigue severity distribution categories were compared between treatment groups using a shift analysis and was visualised using a grouped bar plot. Interaction effects between levodopa treatment and participant characteristics were assessed using logistic regression models, including interaction terms between treatments and respective subgroup variables. For vascular subgroups, significance of interaction effects was evaluated using likelihood ratio tests via ANOVA. The significance of affected vessel subgroups on treatment effects was evaluated using *P*-values from likelihood ratio tests.

Univariable logistic regression models were used to identify potential associations between fatigue and binary variables. No adjustments for confounding were made due to deviation from intention-to-treat in the main analysis.

All statistical analyses were performed using R version 4.4.0 (24 April 2024).

## Results

### Participants and baseline data

**Figure 1:**
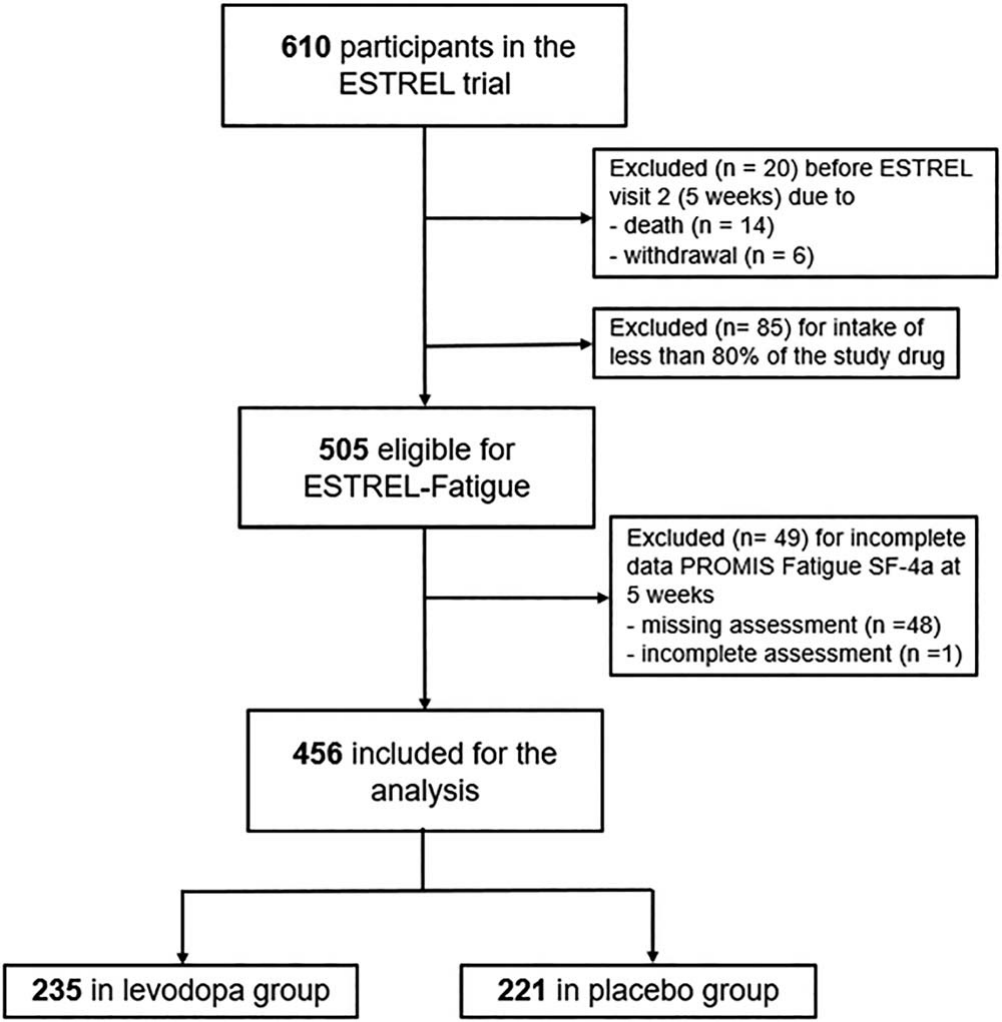
Flowchart of enrolled patients.

A total of 610 patients were enrolled in ESTREL. Of these, 505 (82.8%) were eligible for the ESTREL-Fatigue analyses. Participants not eligible either (i) died (*n* = 14) or withdrew (*n* = 6) before the 5-week assessment, or (ii) had < 80% study medication adherence (*n* = 85). Of these 505 participants, 49 were excluded because PROMIS Fatigue SF-4a data were either incomplete (*n* = 1) or missing (*n* = 48) (9.7% of 505). Therefore, 456 (90.3% of 505) eligible participants were included in ESTREL-Fatigue analyses (Figure 1).

Among the 456 participants (median age of 73 years, 59.2% male), 235 (51.5%) received levodopa and 221 (48.5%) the placebo. No statistically significant difference in baseline characteristics between the levodopa and the placebo arms (Table 1) was detected. Overall, participants had a mean T-score for PROMIS Fatigue SF-4a of 48.51, and 128 of 456 participants (28.1%) reported PSF.

**Table 1:** Baseline demographic and clinical characteristics by allocated treatment.

	**Overall**	**Levodopa**	**Placebo**	**SMD**
***n***	456	235	221	
**Age, median [IQR]**	73 [63, 82]	72 [62, 81]	74 [64, 83]	0.152
**Sex, female (%)**	186 (40.8)	87 (37.0)	99 (44.8)	0.159
**Type of stroke**				
** Acute ischaemic stroke, *n* (%)**	392 (86.0)	203 (86.4)	189 (85.5)	0.025
** Acute haemorrhagic stroke, *n* (%)**	64 (14.0)	32 (13.6)	32 (14.5)	0.025
**Affected vessel (all strokes)**				
** Middle cerebral artery, *n* (%)**	348 (76.3)	176 (74.9)	172 (77.8)	0.069
** Anterior cerebral artery, *n* (%)**	46 (10.1)	30 (12.8)	16 (7.2)	0.185
** Posterior cerebral artery, *n* (%)**	34 (7.5)	15 (6.4)	19 (8.6)	0.084
** Vertebrobasilar arteries, *n* (%)**	80 (17.6)	47 (20.2)	33 (14.9)	0.138
**Acute recanalisation therapy**				0.110
** None**	281 (61.6)	147 (62.6)	134 (60.6)	
** Endovascular therapy**	58 (12.7)	28 (11.9)	30 (13.6)	
** Intravenous thrombolysis**	94 (20.6)	46 (19.6)	48 (21.7)	
** Bridging therapy**	23 (5.0)	14 (6.0)	9 (4.1)	
**Stroke prior to index event , yes (%)**	70 (15.4)	37 (15.7)	33 (14.9)	0.023
**Prestroke mRS, mean (SD)**	0.45 (0.89)	0.42 (0.86)	0.48 (0.92)	0.071
**Stroke severity**				
** mRS at baseline, mean (SD)**	4.14 (0.69)	4.13 (0.72)	4.16 (0.67)	0.044
** FMMA at baseline, mean (SD)**	36.78 (23.18)	37.25 (23.33)	36.28 (23.07)	0.042
** NIHSS at baseline, mean (SD)**	8.05 (3.74)	7.97 (3.80)	8.13 (3.69)	0.044

Abbreviations: FMMA, Fugl–Meyer motor assessment; SMD, standardised mean difference.

### Primary endpoint

**Figure 2:**
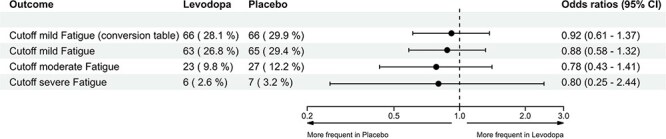
Forest plot of fatigue 5 weeks after ischaemic or haemorrhagic stroke using PROMIS Fatigue SF-4a. Fatigue was measured using different cutoff T-scores of the PROMIS Fatigue SF-4a, showing the number and proportion of participants for each levodopa and placebo group. Odds ratios represent levodopa vs placebo. Mild fatigue = cutoff T-score ≥ 55 using the conversion table and the assessment centre. Moderate fatigue = cutoff T-score ≥ 60. Severe fatigue = cutoff T-score ≥ 70. Abbreviation: PROMIS = Patient-Reported Outcomes Measurement Information System.

Post-stroke fatigue at 5 weeks was present in 63/235 (26.8%) participants in the levodopa group and in 65/221 (29.4%) in the placebo group. The OR was 0.88 (95% CI, 0.58–1.32), with a risk ratio of 0.91 (95% CI, 0.70–1.17) and a risk difference of −2.6% (95% CI, −10.4%–5.2%), indicating no statistically significant difference between groups (Figure 2).

### Secondary endpoints

Applying a T-score cutoff of ≥ 60, PSF was present in 23/235 participants (9.8%) in the levodopa group, and 27/221 participants (12.2%) in the placebo group. The OR was 0.78 (95% CI, 0.43–1.41). For the T-score cutoff of ≥ 70, PSF was present in 6 participants (2.6%) in the levodopa group and 7 participants (3.2%) in the placebo group (OR 0.80; 95% CI, 0.25–2.44; risk ratio 0.81; 95% CI, 0.29–2.25) (Figure 2).

**Figure 3:**
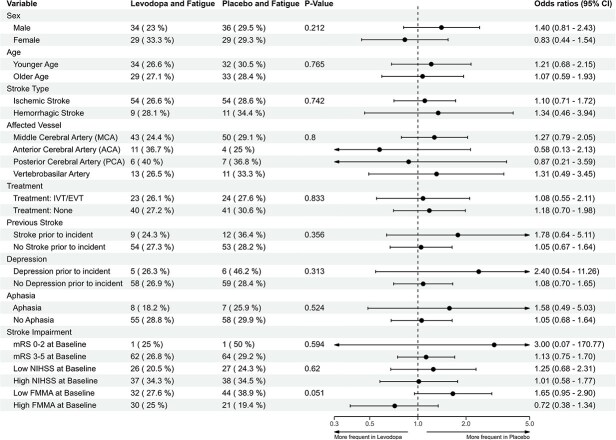
Interaction analysis between patient characteristics and the impact of levodopa on PSF. This interaction analysis shows how participant baseline characteristics are associated with the impact of levodopa on fatigue 5 weeks post-randomisation (percentage of participants with the respective variable and fatigue for each treatment group). The resulting *P*-values reflect the overall significance of the interaction terms. The numeric variables age, NIHSS and FMMA were converted into a binary form using the respective median (73 for age, 34 for FMMA, 7 for NIHSS), the mRS was converted into 0–2 as low and 3–5 as high. Abbreviations: FMMA = Fugl–Meyer motor assessment, PSF = post-stroke fatigue.

**Figure 4:**
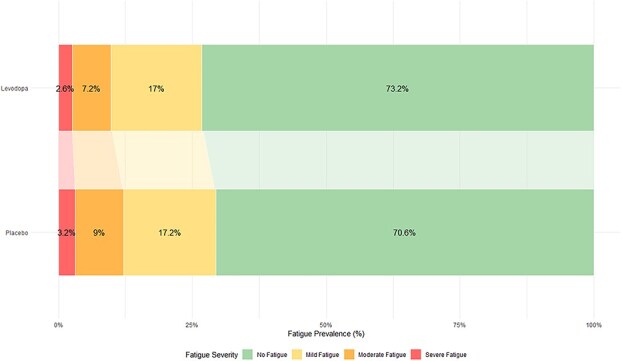
Distribution of the proportion of participants with different levels of fatigue severity in both treatment groups. Shift analysis: no fatigue = T-score < 55, mild fatigue = T-score of 55–60, moderate fatigue = T-score of 60–70 and severe fatigue = 70–80, visualised as proportions.

Interaction analysis did not identify any participant characteristics associated with lower PSF occurrence when comparing them between the 2 treatment groups (Figure 3). Shift analysis showed similar fatigue severity distribution between groups (Figure 4), with mild fatigue being most common (17% in the levodopa group and 17.2% in the placebo group).

**Figure 5:**
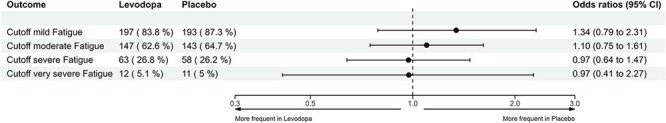
Forest plot of fatigue 5 weeks after ischaemic or haemorrhagic stroke using the single fatigue item of PROMIS-10. The proportion of fatigue was measured using the different answers of the single fatigue item of PROMIS-10, showing the number and proportion of participants for each levodopa and placebo group. Abbreviation: PROMIS = Patient-Reported Outcomes Measurement Information System.

Analysis of PROMIS-10 also showed no significant difference at 5 weeks, based on dichotomised scores on the single fatigue item using the cutoffs “mild” (OR 1.34; 95% CI, 0.79–2.31), “moderate” (OR 1.10; 95% CI, 0.75–1.61), “severe” (OR 0.97; 95% CI, 0.64–1.47) or “very severe” (OR 0.97; 95% CI, 0.41–2.27) (Figure 5). Using this dichotomisation, 85.9% of participants reported fatigue as “mild,” 63.9% as “moderate,” 26.5% as “severe” and 7.7% as “very severe.”

Participants with PSF at 5 weeks had higher NIHSS baseline scores (higher NIHSS = worse outcome) (OR 1.83; 95% CI, 1.21–2.78), and lower baseline FMMA scores (lower FMMA = worse outcome) (OR 1.73; 95% CI, 1.14–2.63) than those without. Female participants (31.2%) reported PSF at 5 weeks more often than men (25.9%), though this difference was not statistically significant (OR 1.29; 95% CI, 0.86–1.96).

**Figure 6:**
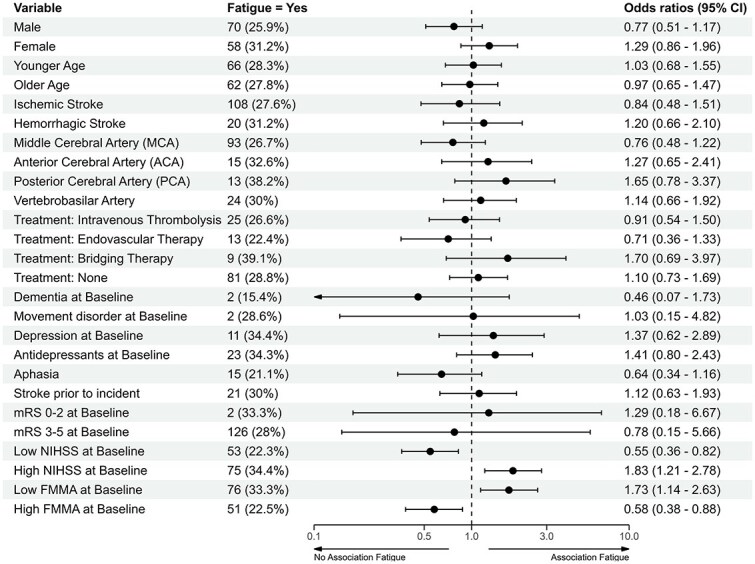
Associations between fatigue and participant characteristics. Age, NIHSS and FMMA were converted into a binary form using the respective median (73 for age, 34 for FMMA, 7 for NIHSS), the mRS was converted into 0–2 as low and 3–5 as high. Abbreviation: FMMA = Fugl–Meyer motor assessment.

In summary, PSF at 5 weeks was associated with higher baseline stroke severity, but showed no significant association with sex, age, stroke type, affected territory or use of acute recanalisation therapy (Figure 6).

### Sensitivity analysis

Participants included in ESTREL-Fatigue had lower baseline NIHSS scores compared with the excluded participants from the total ESTREL population (mean 8.05 vs 8.77, statistically significant *P*-value of .044).

Sensitivity analysis using a mixed-effects logistic regression with a random intercept for centres showed no evidence of centre-related clustering. The estimated treatment effect remained unchanged (OR 1.14; 95% CI, 0.76–1.71), confirming the robustness of the primary analysis.

An intention-to-treat analysis including all 610 randomised ESTREL participants (analysable *n* = 510, missing fatigue data for 100 participants) was performed to determine odds of a participant having fatigue in the treatment group (OR 1.05; 95% CI, 0.72–1.53), yielding results similar to the primary analysis.

The 85 participants excluded for non-adherence were equally distributed between the Levodopa (*n* = 43) and Placebo (*n* = 42) groups (*P* = .91). Fatigue data were available for 54 of these non-adherent participants, of which 21 (38.9%) reported fatigue.

## Discussion

### Key results

In ESTREL-Fatigue the frequency and the distribution of PSF severity did not differ between the levodopa group and the placebo group at 5 weeks.

To our knowledge, ESTREL-Fatigue is the largest trial studying the impact of pharmacological interventions with dopaminergic agents on PSF. Levodopa in ESTREL was administered early and in all participants prior to the start of in-hospital rehabilitation. Enhancement of Stroke Rehabilitation with Levodopa-Fatigue might therefore be considered as a study of prevention rather than as a study of PSF treatment. The lack of any signal of benefit of levodopa found in the ESTREL-Fatigue study does not contradict findings of the MIDAS-trial,^23^ which was given only to participants confirmed to have PSF. Modafinil in Debilitating Fatigue After Stroke (MIDAS) was a randomised, placebo-controlled, phase 2 trial, showing that 200 mg of modafinil daily for 6 weeks was associated with reduced PSF symptoms The MIDAS trial used the multidimensional fatigue inventory compared with placebo in 36 (among 232 screened) stroke patients with PSF. Modafinil is a wakefulness-promoting agent that stimulates monoaminergic pathways to increase the release of histamine, norepinephrine, serotonin, dopamine and orexin.^32^ Additional explanations for the seemingly discrepant results between MIDAS and ESTREL-Fatigue include that (i) modafinil has several modes of action and the effect of dopamine might be less important, and (ii) different time-points of diagnosing PSF (after 5 weeks in ESTREL-Fatigue vs 13 weeks in MIDAS), which may have contributed to differing key findings. To our knowledge, no studies have specifically investigated pharmacological strategies for the prevention of PSF. While interventions that reduce the risk of developing PSF may also prove effective for its treatment, this assumption remains speculative in the absence of direct trial evidence.

The time course of PSF remains incompletely understood. Two longitudinal studies suggest that acute phase PSF may persist between early onset and 18 months and was reportedly associated with poorer mental health outcomes.^33,34^ A meta-analysis reported, that 66% of fatigued patients remained fatigued at follow up (6 studies, 3–18 months post-stroke), while 15% of initially non-fatigued patients developed PSF.^35^

ESTREL-Fatigue analysed data of the ESTREL trial, which primarily focused on motor recovery and was therefore not intended to evaluate the effect of an agent on PSF or use widely accepted diagnostic tools for PSF.

The International Stroke Recovery and Research Alliance (ISRRA) Fatigue Roadmap recommended further research into dopamine re-uptake inhibitors as a promising intervention for PSF.^5^ While our findings did not signal benefit of levodopa on PSF, they do not necessarily negate the positive findings of MIDAS, which used modafinil in a selected population. A new trial (Modafinil and Exercise for Post Stroke Fatigue—MODEX) investigating the effect of Modafinil on PSF (200 mg daily for 56 days vs Placebo)^36^ is ongoing and may deliver additional insights.

### Limitations and further research

This study has limitations. ESTREL was designed and powered to detect between-group differences regarding motor recovery and not PSF and as such, our results and their potential associations should be considered exploratory.

Fatigue assessment is difficult and currently, no single tool specifically measuring PSF captures all fatigue domains.^5^ The PROMIS Fatigue SF-4a was used for detecting presence of PSF in the ESTREL trial, which is validated for various populations with fatigue (US general population,^37^ cancer,^38^ rheumatoid arthritis,^39^ fibromyalgia,^40^ idiopathic inflammatory myopathy,^41^ multiple sclerosis^42–44^), but not yet validated for PSF. The PROMIS Fatigue SF-4a was not among recommended stroke assessment scales^45^ and was not commonly used to identify patients with PSF, despite its use in more recent studies.^46,47^ In 2024, after data collection for ESTREL was completed, the Fatigue Severity Scale (FSS-7) measure was ranked best for PSF studies by a roundtable conducted by ISRRA due to its ability to assess a range of fatigue domains.^5^ The FSS-7 was not used in the ESTREL trial.^26^ While the PROMIS Fatigue SF-4a assesses subjective experience of fatigue and its impact on daily activities, it does not capture specific functional interference domains, motivation, exercise-related fatigue or symptom ranking assessed by the FSS-7. Thus, the use of PROMIS Fatigue SF-4a, as predefined in the ESTREL trial, represents a limitation of this study. Furthermore, PSF assessments based on patient-reported outcome measures may increase the risk of reporting bias.

Although no overall benefit of levodopa for PSF was found, limitations of PROMIS mean it is possible levodopa may have beneficial effects on certain subdivisions or phenotypes of PSF. The necessity to classify PSF into different phenotypes has been shown to be increasingly important.^11^ For example, cognitive fatigue is more common in patients with cortical lesions and patients with subcortical lesions experience more physical fatigue.^48^ Additionally, patients with depression experience reduced motivation and more mental fatigue, while those with anxiety experience more physical fatigue.^49^ Different phenotypes may also explain seemingly contradictory data regarding associations between PSF and patient characteristics. For example, some studies reported an association between PSF and higher age,^8^ some with younger age^50^ and others reported no associations regarding age at all.^1,51^ The interrelationship between stroke impairment level and PSF is also unclear, with some evidence indicating a relationship^51–53^ and other literature supporting no relationship.^1,54^ In ESTREL-Fatigue, PSF was associated with higher stroke severity (higher NIHSS) and motor impairment (lower FMMA) at baseline, highlighting the importance of these factors in the context of PSF. Furthermore, as in many stroke trials,^55^ women were underrepresented in our study population (41%), which limits the generalisability of findings to female patients. In general, the multi-factorial nature of fatigue is unlikely to be successfully treated by a single mechanistic or biological therapy and therefore dopamine reuptake inhibitors alone may not be sufficient treatment for all phenotypes.

We neither analysed the interrelationship with nor corrected for the presence of mental health issues after stroke such as depression, anxiety or sleep disorders. These mental health issues can overlap with certain phenotypes of PSF and may have affected our results.^56–58^ Furthermore, using a treatment that started within the first 7 days after stroke onset and lasting 39 days meant we were unable to answer the question of whether a longer treatment duration or delayed start of levodopa would have a different effect. Post-stroke fatigue was analysed only at a single time point, preventing analysis of its temporal dynamic.

We also did not evaluate relationships between neuroimaging findings. Although there was a study showing patients with reduced functional connectivity between the dorsolateral prefrontal cortex and the caudate nucleus benefited more from modafinil therapy,^59^ a systematic review from 2023 did not find robust evidence of a relationship between PSF and structural imaging features.^60^ Altered brain networks might be more crucial than specific lesion location and screening of risk factors may be more important. Future studies may identify distinct trajectories, associated patient variables and predictors that enable earlier, targeted intervention.

As this sub-study used an unadjusted per-protocol analysis and excluded participants with incomplete PSF data and outcomes, there is a risk of bias due to disruption of the original randomisation. Although non-adherence was balanced between the levodopa and placebo groups, non-adherent participants tended to report fatigue more often, introducing a possible attrition bias, which should be considered in the interpretation of the treatment effect. However, no clear differences in participant characteristics between levodopa and placebo groups was found and the sensitivity analysis using an intention-to-treat approach produced similar findings.

Our study population had an overall mean T-score of 48.51, lower than the pooled general US-population used to calibrate PROMIS Fatigue (T-score of 50). Other studies with stroke survivors in the United States using PROMIS Fatigue showed a mean T-score of 53.2^61^ and 51.5^62^ indicating that our study population is less affected by fatigue. A global meta-analysis showed a pooled prevalence of PSF for Switzerland of 39.84% compared to 52.19% for the United States,^6^ suggesting possible cultural differences.

### Strengths

With 456 analysable participants and minimal missing data, our study benefits from a robust sample size, which stands out compared to the typically smaller cohorts in PSF research (median of 50 participants^63^). We used double blinding to assess patient reported outcomes, which is a major strength compared to most fatigue studies. Our sensitivity analyses yielded treatment effects consistent with the primary analysis.

The single fatigue item (Global08) in PROMIS-10 also did not lead to a significant difference between the 2 treatment groups. The PROMIS-10 showed sufficient reliability, validity and responsiveness to examine global health after stroke^64–66^ and the Global08 item for fatigue showed sufficient validity in a Dutch population.^67^

## Conclusion

We found no evidence that levodopa was associated with a reduced frequency or severity of PSF during in-hospital rehabilitation 5 weeks after stroke onset.

## Supplementary Material

aakag029_Supplemental_Files

## Data Availability

Trial data can be made available upon reasonable request to the corresponding author. Such requests must be accompanied by detailed study proposals, a description of study objectives and a statistical analysis plan. Each request will be checked for compatibility with regulatory (ethics committee) requirements and with patient-informed consent.

## Patient and public involvement

Patients or members of the public were not involved in this study’s design, conduct or recruitment. The research question and outcome measures were based on established clinical priorities and existing scientific evidence.

## Publishing rights statement

The corresponding author has the right to grant on behalf of all authors and does grant on behalf of all authors, a worldwide license to the publishers and its licenses in perpetuity, in all forms, formats and media (whether known now or created in the future), to (i) publish, reproduce, distribute, display and store the contribution, (ii) translate the contribution into other languages, create adaptations, reprints, include within collections and create summaries, extracts and/or abstracts of the contribution, (iii) create any other derivative work(s) based on the contribution, (iv) to exploit all subsidiary rights in the contribution, (v) the inclusion of electronic links from the contribution to third-party material where-ever it may be located and (vi) license any third party to do any or all of the above.
