# Supplementary material for: ESTREL-Fatigue—association of levodopa with post-stroke fatigue
Source: Eur Stroke J. 2026 Apr 7;11(4):aakag029. doi: 10.1093/esj/aakag029 (PMC13131240; doi:10.1093/esj/aakag029)
Supplement: aakag029_Supplemental_Files [file aakag029_supplemental_files.zip › Clinical Study Protocol ESTREL V1.3, 03.07.2019.pdf]

Enhancement of Stroke Rehabilitation with Levodopa (ESTREL): a randomized placebo-controlled trial

Clinical Study Protocol

Study Type:	Clinical trial with Investigational Medicinal Product (IMP)
Study Categorisation:	Risk category C
Study Registration:	Federal Office of Public Health portal (www.kofam.ch; to be registered)<br>ClinicalTrials.gov NCT03735901
Study Identifier:	ESTREL
Sponsor, Sponsor-Investigator or Principal Investigator:	Prof. Dr. med. Stefan Engelter<br>Chair Rehabilitation<br>Felix-Platter Spital Basel<br>Burgfelderstrasse 101 – CH-4002 Basel<br>Phone: +41 61 326 4063<br>Fax: +41 61 326 4108<br>E-Mail: stefan.engelter@fps.ch
Investigational Product:	Levodopa/carbidopa 100/25mg
Protocol Version and Date:	1.3, 03.07.2019

Signature Page(s)

Study number	ClinicalTrials.gov: NCT03735901
Study Title	***Enhancement of Stroke Rehabilitation with Levodopa (ESTREL): a randomized placebo-controlled trial***

The Sponsor-Investigator and trial statistician have approved the protocol version [1.3 (dated 03.07.2019)] and confirm hereby to conduct the study according to the protocol, current version of the World Medical Association Declaration of Helsinki, ICH-GCP guidelines and the local legally applicable requirements.

Sponsor-Investigator:
Prof. Dr. med. Stefan Engelter, Chair Rehabilitation Felix-Platter Spital Basel, Switzerland

Place/Date Signature

Trial Statistician:
Sabine Schädelin, MSc, Universität Basel, Departement Klinische Forschung, CTU Basel, Switzerland

Place/Date Signature

Local Principal Investigator at study site:
I have read and understood this trial protocol and agree to conduct the trial as set out in this study protocol, the current version of the World Medical Association Declaration of Helsinki, ICH-GCP guidelines and the local legally applicable requirements.

Site	Universitätsspital Basel<br>Neurologische Klinik<br>Petersgraben 4<br>CH-4031 Basel<br>Switzerland
Principal investigator	Prof. Philippe Lyrer

Place/Date

Signature

Table of Contents

STUDY SYNOPSIS **7**
ABBREVIATIONS **11**
1. STUDY ADMINISTRATIVE STRUCTURE **15**
1.1 Sponsor, Sponsor-Investigator 15
1.2 Principal Investigator(s) 15
1.3 Statistician ("Biostatistician") 15
1.4 Manufacturer and Supplier of Investigational Medical Product (IMP) and Placebo 15
1.5 Monitoring institution 15
1.6 Data Safety Monitoring Committee 16
1.7 Any other relevant Committee, Person, Organisation, Institution 16
1.7.1 Trial Steering Committee 16
2. ETHICAL AND REGULATORY ASPECTS **16**
2.1 Study registration 16
2.2 Categorisation of study 16
2.3 Competent Ethics Committee (CEC) 16
2.4 Competent Authorities (CA) 17
2.5 Ethical Conduct of the Study 17
2.6 Declaration of interest 17
2.7 Patient Information and Informed Consent 17
2.8 Participant privacy and confidentiality 18
2.9 Early termination of the study 18
2.10 Protocol amendments 18
3. BACKGROUND AND RATIONALE **18**
3.1 Background and Rationale 18
3.2 Investigational Product (treatment, device) and Indication 20
3.3 Preclinical Evidence 20
3.4 Clinical Evidence to Date 20
3.5 Dose Rationale: Rationale for the intended purpose in study 21
3.6 Explanation for choice of comparator (or placebo) 21
3.7 Risks / Benefits 21
3.8 Justification of choice of study population 22
4. STUDY OBJECTIVES **22**
4.1 Overall Objective 22
4.2 Primary Objective 22
4.3 Secondary Objectives 22
4.4 Further objectives 23
4.5 Safety Objectives 23
5. STUDY OUTCOMES **23**
5.1 Primary Outcome 23
5.2 Secondary Outcomes 23
5.2.1 Secondary outcome assessments (all patients) 24
5.2.2 Secondary outcome assessments in selected centers only: 25
5.2.3 Further assessments (selected centers only) 26
5.3 Safety Outcomes 26

6. STUDY DESIGN 26
6.1 General study design and justification of design 26
6.2 Methods of minimising bias 28
6.2.1 Blinding procedures 28
6.2.2 Other methods of minimising bias 28
6.3 Unblinding Procedures (Code break) 28
7. STUDY POPULATION 28
7.1 Eligibility criteria 28
7.2 Recruitment and pre-screening 28
7.3 Assignment to study groups 29
7.4 Criteria for withdrawal / discontinuation of participants 29
8. STUDY INTERVENTION 30
8.1 Identity of Investigational Products (treatment / medical device) 30
8.1.1 Experimental Intervention 30
8.1.2 Control Intervention 30
8.1.3 Packaging, Labelling and Supply (re-supply) 30
8.1.4 Storage Conditions 30
8.2 Dose / Device modifications 30
8.3 Compliance with study intervention 30
8.4 Data Collection and Follow-up for withdrawn participants 30
8.5 Trial specific preventive measures 30
8.6 Concomitant Interventions (treatments) 31
8.7 Study Drug / Medical Device Accountability 32
8.8 Return or Destruction of Study Drug / Medical Device 32
9. STUDY ASSESSMENTS 32
9.1 Assessments of outcomes 32
9.1.1 Assessment of primary outcome 32
9.1.2 Assessment of secondary outcomes 32
9.1.3 Assessments in participants who prematurely stop the study 33
9.2 Procedures at each visit during the Interventional Study Phase 33
9.2.1 Pre-screening (day -7 to day 0 before randomization) 33
9.2.2 Visit 1: (day 0) 33
9.2.3 Study treatment: Run-in, full-dose treatment, tapering phase (day 1-39) 33
9.2.4 Visit 2: (5 weeks +/- 7 days) 33
9.2.5 Visit 3: (3 months +/- 14 days) 33
9.3 Procedures at each visit during the Observational Study Phase 34
9.3.1 Visit 4: 6-months long-term follow-up (6 months +/- 20 days) 34
9.3.2 Visit 5: 12-months long-term follow-up (12 months +/- 30 days) 34
9.4 Additional data collection and visits 34
10. SAFETY 34
10.1 Safety Interventional Study Phase 34
10.1.1 (Serious) Adverse events and other safety related events 34
10.1.2 Definition and assessment of (serious) adverse events and other safety related events .. 34
10.1.3 Reporting of serious adverse events (SAE) and other safety related events 36
10.1.4 Follow up of (Serious) Adverse Events 36
10.2 Safety Observational Study Phase 37

10.2.1 Notification of safety and protective measures (HRO Art. 20) 37
10.2.2 Serious events (HRO Art. 21) 37
11. STATISTICAL METHODS 37
11.1 Hypothesis 37
11.2 Determination of Sample Size 37
11.3 Statistical criteria of termination of trial 38
11.4 Planned Analyses 39
11.4.1 Datasets to be analysed, analysis populations 39
11.4.2 Primary Analysis 39
11.4.3 Sensitivity Analyses 39
11.4.4 Secondary Analyses 40
11.4.5 Interim analyses 40
11.4.6 Safety analysis 40
11.4.7 Deviation(s) from the original statistical plan 40
11.5 Handling of missing data and drop-outs 40
12. QUALITY ASSURANCE AND CONTROL 41
12.1 Data handling and record keeping / archiving 41
12.1.1 Electronic Case Report Forms 41
12.1.2 Specification of source documents 41
12.1.3 Record keeping / archiving 41
12.2 Data management 41
12.2.1 Data Management System 41
12.2.2 Data security, access and back-up 41
12.2.3 Analysis and archiving 41
12.2.4 Electronic and central data validation 41
12.3 Monitoring 42
12.4 Audits and Inspections 42
12.5 Confidentiality, Data Protection 42
12.6 Storage of biological material and related health data 42
13. PUBLICATION AND DISSEMINATION POLICY 42
14. FUNDING AND SUPPORT 43
14.1 Funding 43
15. INSURANCE 43
16. REFERENCES 44
17. APPENDICES 47

STUDY SYNOPSIS

**Sponsor / Sponsor-Investigator**	Prof. Dr. med. Stefan Engelter<br>Chair Rehabilitation<br>Felix-Platter Spital Basel<br>Burgfelderstrasse 101 – CH-4055 Basel<br>Phone: +41 326 4063<br>Fax: +41 326 4108<br>E-Mail: stefan.engelter@fps.ch
**Study Title:**	***Enhancement of Stroke Rehabilitation with Levodopa: a randomized placebo-controlled trial***
**Short Title / Study ID:**	***ESTREL***
**Protocol Version and Date:**	1.3, 03.07..2019
**Trial registration:**	ClinicalTrials.gov NCT03735901
**Study category and Rationale**	Category C. The use of Levodopa/Carbidopa is authorised for the treatment of Parkinson`s Disease. The use of Levodopa/Carbidopa for the pharmacological enhancement of stroke rehabilitation is outside the authorised indication. The investigational medicinal product (IMP) is not authorized in Switzerland.
**Clinical Phase:**	Phase III
**Background and Rationale:**	In stroke medicine, the large body of high-quality evidence proving benefits of acute revascularization therapies and secondary prevention is offset by a large gap of evidence on means to enhance stroke recovery. Levodopa is a promising candidate for the pharmacological enhancement of stroke recovery. Dopamine is a key player in processes of motor learning, reward, and brain plasticity. Preclinical research and studies with healthy individuals suggest that there is scope for benefit from applying levodopa in addition to standardized rehabilitation. Indeed, there are some, however limited and inconsistent data from randomized controlled trials (RCTs) testing levodopa in stroke patients. A meta-analysis across 6 RCTs indicated the possibility of a more favorable outcome in levodopa-treated stroke patients than in control patients. Heterogeneity between trials was considerable. The RCTs differed regarding patient populations (chronic/acute stroke), types of stroke (ischemic/hemorrhagic), dosage and duration of the study treatment, length of follow-up, and outcome measures. None mentioned adaptation of concomitant rehabilitative therapies to the principles of motor learning. Of note, safety concerns were absent. Motor deficits are common and affect quality of life in stroke patients prompting for motor improvement as a top priority. Given the high prevalence and tremendous burden of stroke, a straightforward applicable measure to improve motor outcome is highly relevant. Given the promising but inconclusive clinical trial evidence on benefits, a well-designed randomized controlled trial studying the usefulness of levodopa in enhancing motor recovery after stroke is warranted.
**Primary objective:**	To investigate whether levodopa compared to placebo given in addition to standardized rehabilitation based on the principles of motor learning is associated with a patient-relevant enhancement of functional recovery in acute stroke patients.

**Outcomes:**	***The primary outcome*** is the between-group difference of scores in the Fugl-Meyer-Motor Assessment (FMMA) measured 3 months after randomization.<br><br>**S*econdary outcomes***<br>a) Between-group differences in:<br>i) the FMMA total score at the end of study treatment (at 5 weeks), at 6 and 12 months<br>ii) the FMMA upper extremity and lower extremity score at 5 weeks, 3, 6, and 12 months<br>iii) NIH-Stroke Scale score, modified Rankin Scale, Patient-reported outcomes (PROMIS 10 and 29), Patient-reported assessment of relevance of motor improvement and the Rivermead Mobility Index at 5 weeks, 3, 6 and 12 months<br><br>b) Relative risks of mortality, recurrent stroke, re-hospitalization, serious adverse events and selected other adverse events possibly related to the Investigational Medicinal Product (IMP).<br><br>c) Between-group differences (assessed in a subset of patients in selected centers only) in:<br>Action Research Arm Test, Box-and-Block-Test, Jamar dynamometer test, Motricity index, Trunk control, Functional ambulation categories, 10-meter walk test, Montreal Cognitive Assessment, and daily life activities measured with movement sensors, at 5 weeks, 3, 6 and 12 months
**Study design:**	Multicenter, randomized (ratio 1:1), parallel-group, placebo-controlled superiority trial with blinded patients, care-providers, investigators, and outcome assessors.

**Inclusion / Exclusion criteria:**	***Inclusion criteria:***<br>1. Acute ischemic or hemorrhagic (i.e. intracerebral hemorrhage excluding subarachnoid hemorrhage and cerebal venous sinus thrombosis) stroke ≤ 7 days prior to randomization<br>2. Clinically meaningful hemiparesis (i.e. scoring a total of ≥ 3 points on the following NIH stroke scale score items (i) motor arm, (ii) motor leg, (iii) limb ataxia; a distal arm paresis is equivalent to one of the aforementioned (i-iii)).<br>3. Time of randomization ≥24-hours since thrombolysis or thrombectomy.<br>4. In-hospital rehabilitation required<br>5. Capable to participate in standardized rehabilitation therapy<br>6. Informed consent of patient or next of kin<br><br>***Exclusion criteria:***<br>1. Age < 18 years<br>2. Diagnosis of Parkinson's Disease<br>3. Use of Levodopa mandatory according to judgement of treating physician<br>4. Inability or unwillingness to comply with study procedures including adherence to study drug intake (orally, or via nasogastric tube or percutaneous endoscopic gastrostomy tube)<br>5. Severe aphasia (i.e. unable to follow two-stage-commands)<br>6. Previously dependent in the basal activities of daily living (defined as modified Ranking Scale prior to stroke > 3)<br>7. Pre-existing hemiparesis<br>8. Known hypersensitivity to Levodopa/Carbidopa and other contraindications for Levodopa/Carbidopa as outlined in the summary of product characteristics (as appended to the study protocol)<br>9. Women who are pregnant or breast feeding, or who intend to become pregnant during the course of the study. Women of childbearing age must take a pregnancy test to be eligible for the study.<br>10. Lack of safe contraception, defined as: Female Participants of childbearing potential, not using and not willing to continue using a medically reliable method of contraception for the entire study duration, such as oral, injectable, or implantable contraceptives, or intrauterine contraceptive devices, or who are not using any other method considered sufficiently reliable by the Investigator in individual cases. Female Participants who are surgically sterilized / hysterectomized or post-menopausal for longer than 2 years are not considered as being of child-bearing potential.
**Measurements and procedures:**	*Study enrolment:* ESTREL participants will be randomized (1:1, stratified by center and using a standard minimization algorithm) to either receive treatment with i) Levodopa 100mg/Carbidopa 25mg three times daily or ii) matching placebo three times daily, both concomitant to standardized rehabilitative therapy adapted to principles of motor learning.<br><br>Participants, and all study personnel will be blinded to study treatment. The day after randomization participants will start study treatment which will be administered in a dose escalation phase, a full dosage treatment phase and a tapering phase. Enrolment and randomization will take place in the acute care where the study treatment will be started. Participants will transit to the rehabilitation center during study treatment phase.<br>In the interventional phase of the study, participants will be followed up at in-person visits 5 weeks and 3 months (outcome assessments by clinical examination) after randomization. In the observational phase of the study, long-term follow-ups will be performed at 6 and 12 months after randomization.

**Study Product / Intervention:**	Levodopa 100mg/Carbidopa 25mg or matching placebo three times daily, administered for 39 days in addition to standardized rehabilitative therapy.<br><br>*Dose escalation phase:*<br>day 1-3: IMP/placebo solely in the morning<br>day 4-6: IMP/placebo in the morning and at lunch time<br>*Full dosage treatment phase:*<br>day 7-34: IMP/placebo three times daily<br>*Tapering phase:*<br>day 35-37: IMP/placebo in the morning and at lunch time<br>day 38-39: IMP/placebo solely in the morning
**Control Intervention (if applicable):**	Matching placebo
**Number of Participants with Rationale:**	Enrolment of 610 participants is planned over the duration of 37 months. A drop-out rate of 10% is assumed leaving 549 evaluable patients. Assuming, that the FMMA is normally distributed with a standard deviation of 25 points, 548 patients will allow to detect a mean difference between the Levodopa- and the control-group in the FMMA score of 6 points (which is assumed to be patient-relevant) at 3 months with a power of 80% (two-sided significance level of 5%).
**Study Duration:**	50 months (screening of first patient to finishing the study)
**Study Schedule:**	First-Participant-In (planned): 03-2019<br>Last-Participant-Out (planned): 12-2022
**Investigator(s):**	See Appendix I
**Study Centre(s):**	Multicenter-study. Study sites: stroke centers and units (12 acute centers anticipated) as well as rehabilitation centers (7 centers anticipated) with experience in stroke patient rehabilitation.
**Statistical Considerations:**	The FMMA at 3 months will be analyzed by a mixed effects model, with FMMA-score at baseline as covariate to adjust for differences at baseline and treatment as two-level factor (“levodopa” vs “placebo”) as well as type of event (acute ischemic vs hemorrhagic stroke) as fixed effects. Center will be included as random factor.<br>The secondary outcomes will be analyzed by regression models appropriate to the data type. Missing data will be addressed using multiple imputation techniques by chained equations.
**GCP Statement:**	This study will be conducted in compliance with the protocol, the current version of the Declaration of Helsinki, the ICH-GCP as well as all national legal and regulatory requirements.

ABBREVIATIONS

Provide a list of abbreviations used on the protocol - to be completed

AE	Adverse Event
ARAT	Action Research Arm Test
ASR	Annual Safety Report
BASEC	Business Administration System for Ethical Committees,
BBT	Box- and Block-Test
CA	Competent Authority (e.g. Swissmedic)
CEC	Competent Ethics Committee
CDMA	Clinical Data Management Application
ClinO	Ordinance on Clinical Trials in Human Research (in German: KlinV, in French: OClin, in Italian: OSRUm)
COMT	Catechol-o-Methyl-Transferase
CRF	Case Report Form
eCRF	Electronic Case Report Form
CTCAE	Common terminology criteria for adverse events
CTU	Clinical Trial Unit
DR	Dopamine Receptor
DSMC	Data Safety Monitoring Committee
DSUR	Development safety update report
EDTA	Ethylendiaminetetraacetic acid
FAC	Functional Ambulation Categories
FAS	Full analysis set
FMMA (UE/LE)	Fugl-Meyer Motor Assessment (Upper Extremity / Lower Extremity)
FOPH	Federal Office of Public Health
GCP	Good Clinical Practice
IB	Investigator’s Brochure
Ho	Null hypothesis
H1	Alternative hypothesis
HRA	Federal Act on Research involving Human Beings (in German: HFG, in French: LRH, in Italian: LRUm)
IMP	Investigational Medicinal Product
IIT	Investigator-initiated Trial
ISO	International Organisation for Standardisation
ITT	Intention to treat
JDT	Jamar Dynamometer Test
MD	Medical Device
MedDO	Medical Device Ordinance (in German: MepV, in French: ODim)
MI	Motricity Index
MOCA	Montreal Cognitive Assessment
mRS	modified Rankin Scale
NG tube	Nasogastric (tube)
NIHSS	National Institutes of Health Stroke Scale
PEG	Percutaneous Endoscopic Gastrostomy (feeding tube)
PI	Principal Investigator
PP	Per Protocol
PROMIS	Patient Reported Outcome Measures in Stroke
RCT	Randomized Controlled Trial

RMI	Rivermead Mobility Index
SAE	Serious Adverse Event
SD	Standard Deviation
SDV	Source Data Verification
SNCTP	Swiss National Clinical Trials Portal
SOP	Standard Operating Procedure
SPC	Summary of product characteristics
SSRI	Selective Serotonin Reuptake Inhibor
SUSAR	Suspected Unexpected Serious Adverse Reaction
TCT	Trunk Control Test
TSC	Trial Steering Committee
TMF	Trial Master File
10MWT	10-meter-walk-test

STUDY SCHEDULE

Study phase	Pre-Screening	Enrolment	Interventional phase / main study						Observational phase		
**Visit**		Visit 1					Visit 2	Visit 3	Visit 4	Visit 5	Unscheduled Visit***
**Main purpose**	Eligibility pre-check	Screening, Consent, Enrolment, Randomization	Dose escalation	Transition* to rehab center	Full dose study treatment	Dose tapering	End of treatment	Primary outcome assessment	Long-term folllow up	Long-term follow up	End of study (unscheduled)
**Timepoint**	day -7 to 0	day 0	day 1-6	day (X)	day 7-34	day 35-39	5 weeks (+/- 7 days)	3 months (+/- 14 days)	6 months (+/- 20 days)	12months (+/- 30 days)	unscheduled
Informed consent	-	x	-	-	-	-	-	-	-	-	-
Randomization	-	x	-	-	-	-	-	-	-	-	-
Dispensing of study drug	-	x	-	-	-	-	-	-	-	-	-
Group A (Levodopa)	-	x	x	x	x	x	*End of treatment*	-	-	-	-
Group B (Placebo)	-	x	x	x	x	x	*End of treatment*	-	-	-	-
Distribution of therapy booklet	-	x	-	-	-	-	-	-	-	-	-
Assessments											
Inclusion-/Exclusion Criteria	x	x	-	-	-	-	-	-	-	-	-
Demographics	x	x	-	-	-	-	-	-	-	-	-
Medical History	-	x	-	-	-	-	-	-	-	-	-
Pregnancy Test (females)	-	x	-	-	-	-	-	-	-	-	-
Physical examination	-	x	-	-	-	-	x	x	x	x	x
Concomitant rehabilitative therapies	-	-	x	x	x	x	x	x	x	x	x
Concomitant medication	-	x	-	x	-	-	x	x	x	x	x
Drug accountability	-	-	-	-	-	-	x	-	-	-	[x]
Blood sampling **	-	x	-	-	-	-	-	-	-	-	-
Outcome measures											
FMMA (Primary outcome)	-	x	-	-	-	-	x	x	x	x	x
NIH-Stroke Scale Score	x	x	-	-	-	-	x	x	x	x	x
Modified Rankin-Scale Score	-	x	-	-	-	-	x	x	x	x	x
PROMIS-10	-	-	-	-	-	-	x	x	x	x	x
PROMIS 29	-	-	-	-	-	-	x	x	x	x	x
Patient reported assessment of improvement	-	-	-	-	-	-	x	x	x	x	x
Rivermead Mobility Index	-	x	-	-	-	-	x	x	x	x	x
Action Research Arm Test**	-	x	-	-	-	-	x	x	x	x	x
Box- and Block-Test**	-	x	-	-	-	-	x	x	x	x	x
Jamar dynamometer Test**	-	x	-	-	-	-	x	x	x	x	x
Motricity index**	-	x	-	-	-	-	x	x	x	x	x
Trunk control**	-	x	-	-	-	-	x	x	x	x	x
Functional ambulation categories**	-	x	-	-	-	-	x	x	x	x	x
10-meter walk test **	-	x	-	-	-	-	x	x	x	x	x
Montreal Cognitive Assessment **	-	x	-	-	-	-	x	x	x	x	x
Daily activity measurement with movement sensors **	-	x	-	-	-	-	x	x	x	x	x
Mortality (all cause)	-	-	x	x	x	x	x	x	x	x	x
Recurrent stroke (any)	-	-	x	x	x	x	x	x	x	x	x
AE/SAE	-	-	x	x	x	x	x	x	-	-	[x]

*Transition: no in-person visit, data collection only, at day of transition of the patient from acute care center to rehabilitation center (any day during treatment phase);

** optional, and in selected centers only, blood sampling may be performed at baseline or any follow up visit.

*** An unscheduled “end of study” visit will be performed in case of discontinuation or withdrawal of consent. [x] Assessment only applicable if the unscheduled visit takes place before end of treatment (regarding drug accountability) or before Visit 3 (regarding AE/SAEs); outcome assessments at this visit will be performed upon participants` approval.

STUDY FLOW CHART

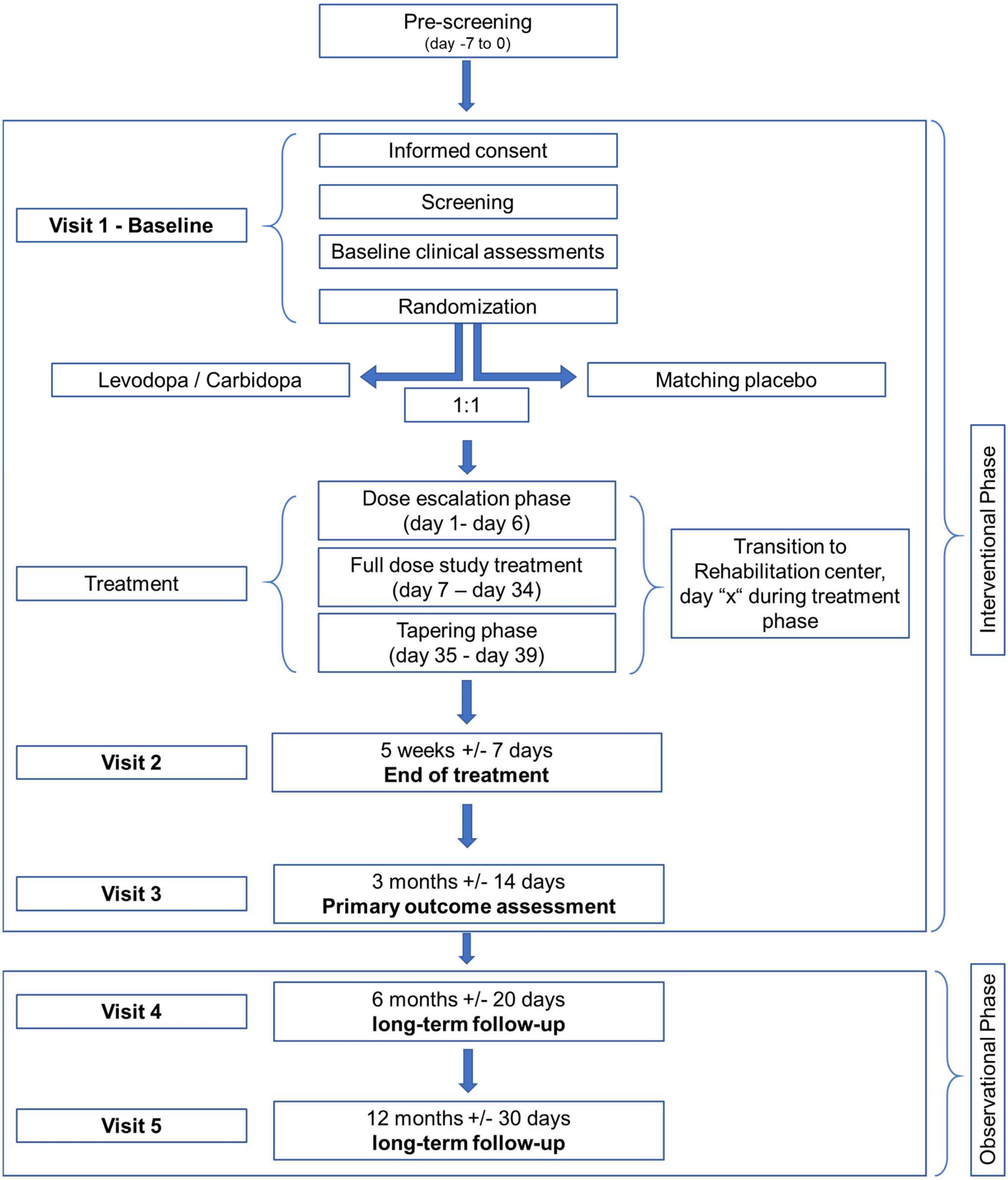

1. STUDY ADMINISTRATIVE STRUCTURE

1.1 Sponsor, Sponsor-Investigator

The Sponsor / Sponsor-Investigator of the study, Prof. Stefan Engelter, has the final responsibility for the design and management of the study, for the collection, analysis and interpretation of all study data and for the writing of scientific reports.

Prof. Dr. med. Stefan Engelter
Head Rehabilitation
Felix-Platter Spital Basel
Burgfelderstrasse 101 – CH-4002 Basel
Phone: +41 61 326 4063
Fax: +41 61 326 4108
E-Mail: stefan.engelter@fps.ch

1.2 Principal Investigator(s)

Prof. Dr. med. Stefan Engelter
Head Rehabilitation
Felix-Platter Spital Basel
Burgfelderstrasse 101 – CH-4002 Basel
Phone: +41 61 326 4063
Fax: +41 61 326 4108
E-Mail: stefan.engelter@fps.ch

1.3 Statistician ("Biostatistician")

Sabine Schädelin, MSc
Universität Basel, Departement Klinische Forschung, Clinical Trial Unit
c/o Universitätsspital Basel │Spitalstrasse 12 │ CH-4031 Basel
Phone: +41 61 556 51 67
Fax: +41 61 265 94 10
Email: sabine.schaedelin@usb.ch

1.4 Manufacturer and Supplier of Investigational Medical Product (IMP) and Placebo

Laboratorium Dr. G. Bichsel AG
Weissenaustrasse 73
3800 Interlaken
Tel.: +41 (0) 33 827 80 00
Fax: +41 (0) 33 827 80 10
Email:
Pasqual.Bichsel@bichsel.ch
Helene.Mohr@Bichsel.ch
Florian.Kunz@Bichsel.ch

1.5 Monitoring institution

Clinical Trial Unit (CTU)
University Hospital Basel
Schanzenstrasse 55
CH-4031 Basel
Switzerland
Tel: +41 61 556 56 25
Fax:+41 61 265 94 10
Email: klaus.ehrlich@usb.ch

1.6 Data Safety Monitoring Committee

An independent Data Safety Monitoring Committee will monitor safety aspects of the trial and monitor the frequency of the clinically apparent safety outcomes listed in Section 11.3 of the study protocol (i.e. mortality, recurrent stroke, SAEs). The DSMC`s responsibilities and roles will be documented in the DSMC charter. The DSMC is primarily responsible for safeguarding the interest of the participants in the trial. An independent statistician will provide the DSMC with the relevant safety data unblinded to individual treatment assignment. All investigators of the study and personnel involved in the study conduct at the CTU Basel, including the trial statistician will remain blinded to these data and analyses performed for safety monitoring. The review and evaluation of efficacy data is not the scope of this DSMC. The DSMC will provide recommendations to the Principal Investigator about stopping, continuing or amending the trial – if necessary – based on the criteria listed in section 11.3 of the study protocol.

1.7 Any other relevant Committee, Person, Organisation, Institution

1.7.1 Trial Steering Committee

The ESTREL Trial Steering Committee (TSC) consists of the Sponsor-Investigator, the Trial Statistician, the ESTREL Medical Network Advisor, designated Principal Investigators at participating ESTREL study centers, a clinical epidemiologist, a therapist, and an external, internationally recognised expert in acute stroke, rehabilitation and pharmacological modification of recovery.
Responsibilities of the ESTREL-TSC are: design of the study (including development of the protocol as well as protocol amendments), the supervision of the study as well as interpretation and reporting of the final study results. The TSC will be advised by the DSMC on early termination or modification of the study as described in sections 1.6 and 11.3 of the study protocol.
Members of the ESTREL-TSC including contact details are listed in the appendix II.

2. ETHICAL AND REGULATORY ASPECTS

Before the study will be conducted, the protocol, the proposed patient information and consent form as well as other study-specific documents will be submitted to a properly constituted Competent Ethics Committee (CEC) and to the competent authorities (CA; Swissmedic / Swiss Federal Office of Public Health (FOPH) / foreign CAs) - in agreement with local legal requirements - for formal approval. Any amendment to the protocol must as well be approved (if legally required) by these institutions.

The decision of the CEC and Swissmedic/foreign CA concerning the conduct of the study will be made in writing to the Sponsor-Investigator before commencement of this study. The clinical study can only begin once approval from all required authorities has been received. Any additional requirements imposed by the authorities shall be implemented.

2.1 Study registration

The study is registered with ClinicalTrials.gov NCT03735901. The study will also be registered in the Swiss National Clinical Trials Portal (SNCTP), a database of the FOPH (www.kofam.ch).

2.2 Categorisation of study

According to the criteria of the Swiss Ordinance on Clinical Trials in Human Research (ClinO, Art. 19) the study falls into Category C. Levodopa 100mg/Carbidopa 25mg is authorized for the treatment of Parkinson`s Disease. The use of Levodopa/Carbidopa for the pharmacological enhancement of stroke rehabilitation in this study is outside the authorized indication. The investigational medicinal product (IMP) is not authorized in Switzerland.

2.3 Competent Ethics Committee (CEC)

The responsible investigator at each site ensures that approval from an appropriately constituted Competent Ethics Committee (CEC) is sought for the clinical study.

All changes in the research activity and all unanticipated problems involving risks to humans will be reported to the CEC. No substantial changes or amendments to the protocol will be made without prior approval by the CEC, except where necessary to eliminate apparent immediate hazards to study participants. Non-substantial protocol amendments will be reported according to chapter 2.10 of the protocol.

The investigator`s duties with regard to reporting of safety outcome events, adverse events and serious adverse events to the Sponsor-Investigator and the CEC/CA is defined in section 10 of the study protocol.

Premature study end or interruption of the study (according to section 2.9 of the protocol) will be reported to the CEC within 15 days. The regular end of the study will be reported to the CEC within 90 days, the final study report shall be submitted within one year after study end.

2.4 Competent Authorities (CA)

The Sponsor-Investigator will obtain approval from the CA before the start of the study.

All changes in the research activity and all unanticipated problems involving risks to humans will be reported to the CA. No substantial changes or amendments to the protocol will be made without prior approval by the CA, except where necessary, to eliminate apparent immediate hazards to study participants. Non-substantial protocol amendments will be reported according to chapter 2.10 of the protocol.

The investigator`s duties with regard to reporting of safety outcome events, adverse events and serious adverse events to the Sponsor-Investigator and the CEC/CA is defined in section 10 of the study protocol.

Premature study end or interruption of the study (according to section 2.9 of the protocol) will be reported to the CEC within 15 days. The regular end of the study will be reported to the CEC within 90 days, the final study report shall be submitted within one year after study end.

2.5 Ethical Conduct of the Study

The study will be carried out in accordance to the protocol and with principles enunciated in the current version of the Declaration of Helsinki, the guidelines of Good Clinical Practice (GCP) issued by ICH, the Swiss Law and Swiss regulatory authority's requirements. The CEC and regulatory authorities will receive annual safety and interim reports and be informed about study stop/end in agreement with local requirements. Requirements of foreign CECs and CAs will be followed.

2.6 Declaration of interest

The investigators involved in this study have no conflict of interest related to the subject of this study.

2.7 Patient Information and Informed Consent

Potential participants will be screened and informed on the study after entering the ward (e.g. Intermediate Care / Stroke Unit or neurology ward). No emergency inclusion will be performed. Before study enrolment (screening) the investigators will explain to each participant and/or his next-of-kin the nature of the study, its purpose, the procedures involved, the expected duration, the potential risks and benefits and any discomfort it may entail. Each participant and/or his/her next-of-kin will be informed that the participation in the study is voluntary and that he/she may withdraw from the study at any time and that withdrawal of consent will not affect his/her subsequent medical assistance and treatment.

The participant will be informed that his/her medical records may be examined by authorised individuals other than their treating physician.

All participants in the study will be provided a participant information sheet and a consent form describing the study and providing sufficient information for participants to make an informed decision whether to participate in the study or not.

Patients with severe (particularly sensory) aphasia (i.e. unable to follow two-stage-commands) are excluded from the study. In some patients (e.g. mild aphasia or neglect) written informed consent by a next-of-kin might be necessary as it may not be possible to ascertain that the participant fully understands the content, procedures within the study as well as potential benefits and harms. A next-of-kin will be fully informed by local investigators on the objective of the study, the course of the study as well as potential benefits or harms for the participant.

Eligible participants and/or their next-of-kin should read and consider the statement before signing and dating the informed consent form. Before signing the informed consent form, eligible participants and/or their next-of-kin will be given sufficient time to decide on the participation in the study. The participant and/or his/her next-of-kin will be given a copy of the signed document. The consent form must also be signed and dated by the investigator (or his designee) at the same time as the participant and/or his/her next-of-kin signs, and it will be retained as part of the study records. The formal consent of a participant and/or his/her next-of-kin, using the approved consent form, must be obtained before the participant is

submitted to any study procedure.
As soon as possible, deferred informed consent by the participant will be obtained at one of the study follow up visits if inclusion in the study was done upon informed consent by a next-of-kin.

Study participants will not receive any financial compensation for the participation in the study. Refund for travel costs may be requested as outlined in section 7.2.

2.8 Participant privacy and confidentiality

The investigator affirms and upholds the principle of the participant's right to privacy and shall comply with applicable privacy laws. Especially, anonymity of the participants shall be guaranteed when presenting the data at scientific meetings or publishing them in scientific journals.
Individual subject medical information obtained as a result of this study is considered confidential and disclosure to third parties is prohibited. Subject confidentiality will be further ensured by utilising subject identification code numbers to correspond to treatment data in the computer files.
For data verification purposes, authorised representatives of the Sponsor-Investigator, the CEC or CA may require direct access to parts of the medical records relevant to the study, including participants' medical history.

2.9 Early termination of the study

The Sponsor-Investigator may terminate the study prematurely according to certain circumstances, for example:

- ethical concerns
- insufficient participant recruitment
- when the safety of the participants is doubtful or at risk
- alterations in accepted clinical practice that make the continuation of a clinical trial unwise
- early evidence of harm of the experimental intervention

The study may be stopped for safety reasons. The DSMC will monitor safety aspects of the trial and monitor the frequency of the clinically apparent safety outcomes listed in Section 11.3 of the study protocol (i.e. mortality, recurrent stroke, SAEs). The DSMC will provide recommendations to the Principal Investigator and the TSC about stopping, continuing or amending the trial – if necessary – based on the criteria listed in section 11.3 of the study protocol.

2.10 Protocol amendments

Substantial amendments are only implemented after approval of the CEC and CA respectively. Under emergency circumstances, deviations from the protocol to protect the rights, safety and well-being of human subjects may proceed without prior approval of the sponsor and the CEC/CA. Such deviations shall be documented and reported to the sponsor and the CEC/CA as soon as possible.
All non-substantial amendments are communicated to the CA as soon as possible if applicable and to the CEC within the Annual Safety Report (ASR).

3. BACKGROUND AND RATIONALE

3.1 Background and Rationale

In stroke medicine, there is a large body of high-quality evidence the benefits and harm of acute revascularization therapy (e.g. thrombolysis, thrombectomy) and of secondary prevention (e.g. carotid surgery, antithrombotic agents). However, it is unclear, whether putative means to enhance recovery after stroke – in particular of motor deficits – are beneficial.[1] Various pharmacological approaches to enhance motor recovery after stroke have been evaluated.[1] Safety concerns emerged for some agents like piracetam[2] and amphetamines.[3] Although most evidence is available for selective serotonin reuptake inhibitors,[4] due to heterogeneity between trials and methodological limitations a Cochrane review rendered no definitive recommendation.[5] A promising alternative is levodopa.[1] Dopamine regulates many aspects of neural functioning, including excitability, synaptic transmission, plasticity,

protein trafficking and gene transcription.[1, 6] Dopamine has a key role in brain processes, such as movement, learning, reward and plasticity[7, 8] and it is relevant for motivation,[9] activational aspects such as speed, vigor and persistence of activities,[10] action learning,[11] and control of voluntary movements.[12] There is evidence that neuroplasticity is improved by levodopa in stroke patients as well as in healthy subjects.[13-15] In healthy, elderly subjects, levodopa enhanced the ability to form a motor memory to levels of healthy young subjects.[13] Likewise, encoding a motor memory with training was shown to be enhanced in chronic stroke patients receiving levodopa.[14] Furthermore, a positive correlation of levodopa metabolite levels and the effectiveness of skill learning has been shown.[16] In healthy subjects, the intake of levodopa has also been associated with improved straight-walking tendency and the effect of veering less when walking blindfolded along a straight line.[17] On the contrary, in patients with Parkinson`s disease - in whom the dopaminergic system is severely affected - skill learning is impaired.[18] The potential usefulness of levodopa in recovery processes is further supported by preclinical animal experiments, indicating the close relationship between dopamine and mechanisms of neuronal plasticity. The neocortex, and in particular the motor cortex is an area with dense dopaminergic nerve terminals with dopamine receptors (DR) D1 and D2 receptors.[15] DR1 and DR2 receptors are also present in glial and immune cells,[19, 20] putatively contributing to a coordinated synthesis of neurotrophic factors,[21] and anti-inflammatory actions.[19, 22] On the cellular level, the activation of D1R and D2R may modulate the activity of N-methyl-D-aspartate receptors in the peri-infarct area through the activation of protein kinase A.[23] Thus, DR signaling and activation of ionotropic glutamate receptors have reportedly been implicated in long-term plasticity processes after brain injury.[24] Hence, it is reasonable to assume that the recovery-promoting effect of levodopa treatment is achieved by an enhanced activation of the dopamine system in the motor cortex to promote motor skill learning.[25]

The first randomized controlled trial (RCT) on this topic included stroke patients 3 weeks to 6 months after stroke and found, that levodopa (100 mg per day for three weeks) as add-on treatment to physical therapy significantly improved motor recovery compared to physical therapy alone (Rivermead motor assessment: difference between levodopa and placebo 3.9 points; 95% CI 0.55-7.2).[26] This beneficial effect was sustained three weeks after levodopa treatment had been stopped.[26] However, this study was small (47 analyzed patients), and had a high risk of bias indicated by unbalanced baseline characteristics and due to high attrition. Two subsequent factorial RCTs were also small (overall 29 patients receiving levodopa) and had a high risk for various biases. Both did not reproduce this beneficial effect.[27, 28] Further in stroke related aphasia, patients receiving levodopa (100mg per day) eight weeks after stroke onset experienced greater improvement in verbal fluency and repetition compared to placebo.[29]

The UK-based 'Dopamine in Rehabilitation of Stroke' (DARS)-study is the only larger clinical trial evaluating dopaminergic agents in stroke rehabilitation. DARS did not indicate a benefit of the applied intervention (a single levodopa 100mg dosage before a therapy session). There was no clear difference in the proportion of patients walking independently at 8 weeks (primary outcome; OR 0.78, 95% CI 0.53, 1.15). The meaning of these findings are, however, a challenge to construe.[30] [31] In addition, the intensity of rehabilitation sessions in DARS was lower than recommended (in DARS on average 22 sessions (SD 10)) during the 8-week treatment period (i.e. less than 3 times per week). Current guidelines (United States) recommend 3 hours of therapy per weekday. In addition, it might have been a challenge in practice to administer the study drug within the required time period of each therapy session (the protocol required levodopa to be given 45-60 minutes prior to each therapy session, although 0-15 minutes were also acceptable). Finally, the expected effect size (i.e. a 50% increase in the rate of patients walking independently 8 weeks post randomization as compared to placebo) might have been overly optimistic, with limited statistical power.

It is noteworthy, that indicators of harm were absent in the aforementioned studies, and that levodopa is a well-tolerated agent used for other conditions such as Parkinson's disease.[26-28, 32, 33]

ESTREL takes into account lessons learnt from previous research on this field as follows:

(i) in ESTREL, levodopa will be administered three times a day (tid) as preclinical data point towards beneficial effects of continuous levodopa administration on learning abilities.[25] Furthermore, compared to a single dose administration, the tid-approach minimizes the risk of decoupling the study medication from rehabilitation sessions due to medication intake too early or too late.

(ii) Concomitant rehabilitative therapy will be standardized and adapted to the principles of motor learning.

(iii) Inclusion into the study will be as early as possible but not later than 7 days post-stroke onset, which takes into account current knowledge of neuroplasticity and follows recent consensus recommendations on target timepoint to start rehabilitative measures in stroke patients.[34]

(iv) The timepoint of primary outcome measurement (at 3 months post-stroke) as well as the primary outcome measure (Fugl-Meyer-Motor Assessment, FMMA) are also in line with recent consensus recommendations on outcome measurements in stroke recovery and rehabilitation trials.[35]

In summary, levodopa may theoretically enhance motor recovery after stroke, through its action on any of the several different neural systems, suggesting that there is scope for benefit from applying levodopa in addition to rehabilitative therapy (i.e. physical, occupational and speech therapy) in stroke patients.[15, 25, 36-38]

3.2 Investigational Product and Indication

White capsules of a combination of Levodopa 100mg and Carbidopa 25mg or placebo will be produced by Laboratorium Dr. G. Bichsel AG, 3800 Unterseen, Switzerland. Labelling will be performed according to the document «Anleitung Notifikations-dossier» on the Swissmedic homepage and in line with annex 13, volume 4 of EUDRALEX. A sample of the label will be enclosed to the Swissmedic application.
Detailed Information on the Investigational Medical Product (IMP) are given in the appendix III.

3.3 Preclinical Evidence

Preclinical in-vivo studies with Levodopa/Carbidopa did not reveal toxic or genotoxic effects or effects that could have clinical relevance if used in humans.
A summary of preclinical data, including findings of animal studies is outlined in section 3.1. of the study protocol and is included in the Summary of Product Characteristics in the appendix III to this protocol.

3.4 Clinical Evidence to Date

Data on clinical trials using levodopa in stroke are summarized in two, rather limited systematic reviews lacking information on recent studies.[39, 40]
In a rapid systematic review searching Medline, the Cochrane Library and clinicaltrials.gov using (“stroke” AND “levodopa”) and related terms combined with standard filters for randomized controlled trials the following trials were identified and included in a meta-analysis (search performed August 17th 2017). Results from the aforementioned DARS-trial were also included in this analysis.
Results from 6 RCTs - including DARS - comparing levodopa versus control in stroke patients, for which data on motor outcome stratified to the type of study treatment were available.[26-28, 41-43] A non-significant trend towards a more favorable motor outcome in levodopa-treated stroke patients as compared to control patients (forest plot, Figure 1 below) was observed in the meta-analysis. Importantly, the heterogeneity between trials was considerable. (see appendix IV for a summary of these studies).

	Levodopa			Control				Std. Mean Difference	
Study or Subgroup	Mean	SD	Total	Mean	SD	Total	Weight	IV, Random, 95% CI	Year
Celik 2004	9.1	4.5	14	7.3	4.5	12	12.1%	0.39 [-0.39, 1.17]	2004
Acler 2005	23	6	10	22	6	10	10.7%	0.16 [-0.72, 1.04]	2005
Scheidtmann 2001	15.4	3.48	22	11.3	3.48	25	14.5%	1.16 [0.54, 1.78]	2005
Sonde 2007 (with AMPH co-treatment)	22.6	7.8	3	37.9	23.7	5	5.3%	-0.67 [-2.17, 0.83]	2007
Sonde 2007 (without AMPH co-treatment)	25.9	10.6	6	39.4	9.1	7	7.1%	-1.28 [-2.52, -0.04]	2007
Lokk 2011 (without MPH co-treatment)	66.3	31.7	20	53.4	34.4	20	14.4%	0.38 [-0.24, 1.01]	2011
Lokk 2011 (with MPH co-treatment)	57.7	37.1	19	57	35.3	19	14.3%	0.02 [-0.62, 0.65]	2011
Ford 2019 (DARS-trial)	3.1	3.1	308	3.3	3	295	21.7%	-0.07 [-0.23, 0.09]	2015
**Total (95% CI)**			**402**			**393**	**100.0%**	**0.15 [-0.25, 0.55]**	

Heterogeneity: $Tau^2 = 0.18$; $Chi^2 = 21.20$, $df = 7$ ($P = 0.003$); $I^2 = 67\%$
Test for overall effect: $Z = 0.73$ ($P = 0.46$)

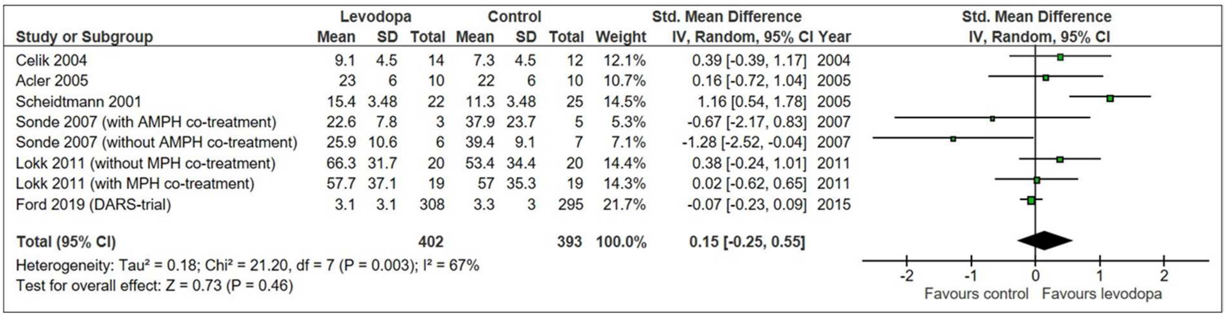

[Figure 1: Meta-analysis assessing the impact of levodopa on motor recovery after stroke. Celik 2004 reported the Rivermead Motor assessment (RMA) score as change from baseline after 6 weeks only; Acler 2005 and Scheidtmann 2001 reported the RMA after 5 and 6 weeks; Sonde 2007 reported the Fugl-Meyer (FM) leg and arm score after 3 months (leg score results used here); Lokk 2011 used FM score after 3 months; DARS 2019 reported the manual ability (ABILHAND) score after 8 weeks. Sensitivity analyses excluding Celik 2004 or using FM arm score results in Sonde 2007 had consistent findings and did not explain statistical heterogeneity.
Abbreviations: AMPH: amphetamine; MPH: methylphenidate]

Furthermore, the RCTs differed largely with regard to patient populations (i.e., chronic or acute stroke), types of stroke (ischemic or hemorrhagic), dosage and duration of the applied study treatment (i.e. levodopa/control), lengths of follow-up, and the choice of the primary outcome measures. Information about the concomitant rehabilitative therapy was sparse and standardization was lacking. Of note, major safety concerns were absent in these RCTs.
Thus, given promising but overall inconclusive clinical trial evidence on benefits, a well-designed, randomized controlled trial studying the usefulness of levodopa in enhancing recovery after stroke is warranted.

3.5 Dose Rationale: Rationale for the intended purpose in study

The experimental intervention in this trial will be Levodopa 100mg/Carbidopa 25mg given three times a day (tid). As detailed in sections 6.1 and 8.1.1 of the protocol there will be a dose escalation (days 1-6) and a tapering phase (days 35-39). There is no data available on dose escalation and tapering of the IMP in the studied population. According to the Swiss Arzneimittelkompendium[44], there are recommendations on dose escalation and full dosage treatment of combinations of Levodopa/Carbidopa in Parkinson`s Disease which take into account tolerability and treatment response. Dose escalation of the IMP/Placebo treatment in ESTREL is adapted to these recommendations.[44] (see appendix III of the protocole for further additional information) The fixed dose escalation scheme in ESTREL is taking into account that no immediate treatment response (as it would be expected in Parkinson`s Disease) which could serve as guidance for the dosage given to the patient, is expected. Tolerability of the IMP will be monitored. If required, dose adaptations are allowed as outlined in detail in section 7.4. In the Swiss "Arzneimittelkompendium", there are – for Parkinson`s- Disease – no recommendations for dose tapering of Levodopa/Carbidopa. In case of abrupt treatment discontinuation patients should be monitored closely. In ESTREL the IMP will be tapered carefully over a period of 5 days to ensure patient safety.

The aforementioned (section 3.4) existing studies on use of levodopa in stroke rehabilitation explored a single dose administration of the agent, given - according to the protocol - at least 30 minutes[26] or 45 to 60 minutes prior to a rehabilitation treatment session,[43] although 0-15 minutes were also acceptable.[43] The peak effect of levodopa can be expected 0.5 to 2 hours after an oral dose.[43] Animal research demonstrated the beneficial effect of a continuous levodopa administration on learning abilities, suggesting that a tid-administration is preferable regarding neuronal plasticity.[25] In addition, a tid-administration is established to be safe and effective for Parkinson's disease. Furthermore, it avoids failures in coordinating the timing of medication intake with that of rehabilitative therapy sessions. The usage of 100 mg (rather than a higher or a lower dose) of levodopa for each single study drug administration, considers the findings of prior studies in humans using 3 different dosages. Different dosages of levodopa resulted in different plasticity effect in human motor cortex.[45, 46] In both studies, 100 mg single dosages prolonged facilitatory and inhibitory plasticity, whereas 25 mg and 200 mg, respectively abolished plasticity effects. A dose of 100 mg for each single administration, was also used in studies about enhanced learning in healthy humans[38, 47] and in the RCTs about stroke motor recovery.[26, 28, 43]

3.6 Explanation for choice of comparator (or placebo)

There is no established active comparator, therefore the comparator used in ESTREL will be matching placebo (identical in aspect, texture, and taste) provided by Bichsel Pharmacy, Interlaken, Switzerland.

3.7 Risks / Benefits

It is expected that all study participants will be subject to in-patient stroke treatment and stroke rehabilitation during the whole study treatment period and will thus be very closely monitored by medical staff for the occurrence of any potential adverse effects.

Potential benefits for the participants would be the effect of enhancement of post-stroke recovery (in the Levodopa/Carbidopa group). All participants will likely benefit from the participation in concomitant, standardized rehabilitative therapy throughout the study period.

No specific AEs related to the study intervention are expected in the placebo-group. In the DARS trial there was no difference in the occurrence of SAEs in the intervention group compared to the placebo group (mean number of SAEs in all patients 1.2 (SD 0.6) within 8 weeks).

Adverse events (AEs) that have been associated with Levodopa/Carbidopa, particularly dyskinesia, nausea, dizziness or headache, (according to the DARS-trial protocol and according to the Summary of product characteristics, appendix III) are outlined in section 10.1.1. of the study protocol. These AEs are pre-specified AEs to be assessed in ESTREL.

We expect that potential adverse effects of the study treatment (which ends 5 weeks after randomization) in both groups would occur during the 5-week treatment phase or to a maximum within 7 weeks thereafter (i.e. within 3 months after randomization which is the time frame of the interventional study phase).

Based on pharmacokinetic data(see appendix III), no adverse effects of the IMP treatment are expected in the observational phase (i.e. 3 to 12 months) of the study as multiples of the IMP half-life and wash-

out periods will be already reached at the end of the interventional phase (i.e. at 3 months after randomization).

3.8 Justification of choice of study population

The present study will enrol patients with acute ischemic or hemorrhagic (i.e. intracerebral hemorrhage excluding subarachnoid hemorrhage and cerebal venous sinus thrombosis) stroke effecting a clinically meaningful hemiparesis i.e. scoring a total of ≥ 3 points on the following NIH stroke scale score items (i) motor arm, (ii) motor leg, (iii) limb ataxia (a distal arm paresis is equivalent to one of the aforementioned (i-iii)) and thus requiring in-hospital rehabilitative therapy. Inclusion and exclusion criteria are provided in the section STUDY SYNOPSIS (see above, section Inclusion and Exclusion criteria).

Patients with severe aphasia (i.e. unable to follow two-stage commands) are excluded from the study, assuming that these patients would not be capable to follow the study procedures or to participate in concomitant standardized rehabilitative therapy. In case a patient is capable of following the study procedures and of participation in concomitant standardized rehabilitative therapy but is not capable of understanding the content and procedures within the study as well as potential benefits and harms (e.g. with moderate aphasia), (additional) written informed consent by a next-of-kin is necessary. Including such patients ensures improved external validity of findings to routine clinical care, where such individuals also belong to the target population of the tested intervention. In these cases, a next-of-kin will be fully informed by local investigators on the objective of the study, the course of the study as well as potential benefits or harms for the participant. If needed, written informed consent will be obtained by a next-of-kin of the potential participant before any study procedure is performed. The next-of-kin will be asked to decide on the participation of his/her relative in the study taking the patients` presumed will on a participation into account. As soon as possible, deferred informed consent by the participant will be obtained at one of the study follow up visits.

The maximum time window from stroke onset to Visit 1 is 7 days, thus inclusion of patients in emergency situations or the hyperacute stroke phase is not intended or necessary.

4. STUDY OBJECTIVES

4.1 Overall Objective

The objective of the study is to investigate the benefits and harms of Levodopa / Carbidopa 100/25mg given three times daily in addition to standardized rehabilitative therapy compared to matching placebo in stroke rehabilitation.

4.2 Primary Objective

To investigate whether Levodopa/Carbidopa compared to matching placebo given in addition to standardized rehabilitation based on the principles of motor learning is associated with a patient-relevant enhancement of functional recovery in acute stroke patients as measured by the between-group difference of final scores in the Fugl-Meyer-Motor-Assessment (FMMA) 3 months after randomization.

4.3 Secondary Objectives

Secondary objectives of the study are:

1) To study whether levodopa compared to placebo given in addition to standardized rehabilitative therapy in patients with acutestroke is associated with

a) improvements of physical function based on the patient’s self-assessment
b) improvement in patient-self assessed general health aspects, pain, mood, anxiety, fatigue and social participation
c) long-term sustainability of a patient-relevant improvement of motor function
d) improvement of selective hand and wrist movement
e) a higher rate of patients walking independently of the help of another person.
f) less severe impairment
g) a higher level of activity of daily living

h) improvements of quality of life
(i) better cognitive performance
(j) no signals of harms (i.e. indications for increased all-cause mortality, recurrent stroke or serious adverse events).

4.4 Further objectives

a) to compare 3-month functional outcome of stroke patients participating in ESTREL to those not participating based on routine data of the Swiss Stroke Registry.

b) to collect information on genetic markers and other biomarkers including brain imaging (computed tomography, magnetic resonance imaging or functional magnetic resonance imaging (fMRI) obtained in clinical routine or observational studies) to allow for explanatory analyses of treatment response.

4.5 Safety Objectives

To compare the occurrence of the following measures of potential harm between the two treatment groups: mortality, recurrent stroke, serious adverse events, and non-serious, pre-specified adverse events (see section 10.1.1) possibly related to the IMP.

5. STUDY OUTCOMES

5.1 Primary Outcome

The primary efficacy outcome is the between-group difference of scores in the Fugl-Meyer-Motor Assessment (FMMA)[48] measured 3 months after randomization.

The FMMA is a stroke-specific impairment index designed to assess motor recovery. Scale items are scored on the basis of ability to complete the item using a 3-point ordinal scale (0=cannot perform; 1=performs partially and 2= performs fully). FMMA total scores range from 0 (no movements) to 100 (normal movements) with 66 points for movements of the upper limbs (FMMA-UE) and 34 for those of the lower limbs (FMMA-LE).[49, 50] All assessments are done by a trained outcome assessor. The use of the FMMA to study motor recovery is widely recommended in several stroke rehabilitation guidelines.[35, 51] The FMMA has been considered the gold standard, due to excellent psychometric properties.[49, 52] Data are available on reliability, validity, sensitivity to change, minimal clinically important difference[53, 54] and item functioning. A difference of 5.25 points for the upper extremity and 6 points for the lower extremity part of the score are described as minimal clinically important difference.[53, 54] For ESTREL, based on these data, 6 points difference are considered a patient-relevant difference between both treatment groups for the primary endpoint. To illustrate this, a 6 point increase in the FMMA translates into a recovery of shoulder function or recovery of hip function in the lower extremity.[48] The FMMA has been used in smaller studies using levodopa[27, 28] for improvement of motor recovery and a large randomized trial conducted in the same patient population as in ESTREL (the FLAME study), which tested Fluoxetine to enhance motor recovery and which provides additional useful information supporting sample size calculations.[4]

5.2 Secondary Outcomes

The following secondary outcomes will be assessed during the study period (see section STUDY SCHEDULE for details on timepoints of assessments). Some secondary outcomes will be assessed in a subset of patients in some centers only (where these assessments are part of clinical routine care), to improve feasibility and minimize the burden to patients resulting from the outcome assessment procedures (see section STUDY SCHEDULE).

5.2.1 Secondary outcome assessments (all patients)

The NIH-Stroke Scale score (NIHSS)[55] is the most widely used score to measure and quantify the overall clinical deficit in the acute phase and during follow-up. This score can be measured in short time with rather little required use of resources.
The modified Rankin Scale Score (mRS)[56] is the most widely used score to measure 3-month outcomes on the activity level. This includes nearly all acute stroke treatment trials and several trials about pharmacological enhancement of motor recovery like the DARS trial, and the FLAME trial.[4] Thus, using the mRS allows for a comparison of any treatment effect in ESTREL with that observed in other studies. Furthermore, mRS-data about 3 month-outcome will also become available in the Swiss Stroke Registry for patients not participating in ESTREL. These data offer the opportunity of an external comparison group and assessment of external validity and generalizability of the findings in ESTREL. In addition, the mRS can be obtained with the simplified mRS questionnaire, which is validated, easy to administer, translatable into multiple languages, and can be performed by raters with diverse professional experiences and skill levels with substantial reliability.[57, 58] As a limitation, the mRS has been reported insensitive to detect aspects of outcomes relevant to patients.[58]

Stroke rehabilitation outcomes for disease specific morbidity and quality of life - PROMIS:
Stroke patients have symptoms that are not entirely captured by the conventional or clinician-reported outcomes.[58] Thus, recently, the Patient-Reported-Outcome-Measure-Information Systems (PROMIS) have been recommended by the U.S. National Institutes of Health (http://www.healthmeasures.net/explore-measurement-systems/promis).[58, 59] PROMIS has been developed to include health aspects, which are (i) important for the patient, (ii) can be reliably captured through patient reports, and (iii) can increase statistical power to detect meaningful differences between treatment groups.[58] PROMIS is based on the item response theory. The mean of each scale is 50, reflecting the mean of the general population, and the standard deviation is 10. The minimally clinical important difference is deemed to be one-half of the standard deviation.[58] In 2016, the “International Standard Set of Patient Centered Outcome Measures (ICHOM)” after stroke has been published.[60] It focuses on the patient perspective of recovery and outcome and advocated, as novel aspects, the use of PROMIS. To take these recent developments and standardizations into account and ensure future applicability and usefulness of our results, these novel techniques are adopted in ESTREL. This includes the use of:

(i) PROMIS-29[61] for the patient-self-assessment in the following categories: 1) physical function, 2) anxiety, 3) depression, 4) fatigue, 5) sleep disturbance, 6) ability to participate in social roles and activities, 7) pain interference and 8) pain intensity. Each of the domains 1 to 7 are assessed with 4 questions. Items are scored on 1 of 5 levels based on the ability of the participant to perform activities or the self-assessment of the participant in the various domains. Pain intensity is scored on a visual analogue scale of 0 to 10.

(ii) PROMIS 10[60, 62] addresses general health aspects, quality of life, pain, mood, anxiety, fatigue and social participation. PROMIS-10 covers the outcome domains considered most important by a recent expert panel.[58]

(iii) Patient-reported assessment of relevance of motor improvement: participants will be asked whether they have improved in motor function since the last study visit and if so, whether this improvement is relevant in their personal perception. Improvement in the FMMA will be compared to the patient-reported relevance of improvement.

The Rivermead Mobility Index (RMI) is a measure of activity. While other scales (FMMA and NIHSS) focus on body structure and function rather than activity or disability, this score focuses on the more important patient`s view on recovery in the activity level. The RMI has 15 items that measure the ability of patients to make postural adjustments (e.g, move in bed), transfer (e.g. between bed to chair), walk, and use stairs and is scored from 0-15 points. It can be used to assess the clinician’s perspective on patient’s ability as well as patient’s ability about the own perspective.[43] The RMI has been characterized as internally consistent, robust and valid and responsive.[52] The minimal detectable change was reported to be 2.2 points.[63] However, a clinically meaningful difference is not established for the RMI (Rehabilitation Institute of Chicago, Center for Rehabilitation Outcomes Research, Northwestern University Feinberg School of Medicine Department of Medical Social Sciences Informatics group, www.rehabmeasures.com). A RMI score of ≥ 7 translates into the ability of the patient walking independently of the assistance of another person. The use of the RMI in ESTREL allows for a

comparison of ESTREL findings with those of the DARS trial (follow-up-visit 8 weeks) and – for the proportion of patients able to ambulate without the help of another person – with both the DARS trial and the small levodopa trial of Scheidtmann et. al.[26]

Potential harm will be assessed by the following measures: in the interventional phase of the study, mortality (all cause), recurrent stroke (any), serious adverse events, pre-specified non-serious adverse events possibly related to the IMP (see section 10.1.1) will be assessed. In the observational phase of the study (3 to 12 months post-randomization) mortality (all cause) and recurrent stroke (any) will be assessed.

5.2.2 Secondary outcome assessments in selected centers only:

In contracts with each study site it will be determined which of the following outcome assessments will be performed specifically at the participating site.

The Motricity Index (MI) consists of two subscales, one for the UE (total score range 0 to 100) and one for the LE (total score range 0 to 100).[64] It measures isometric muscle strength and the UE subscale has a predictive value regarding recovery of upper limb activities.

Trunk Control Test: The sitting balance item of the Trunk Control Test (TCT) assesses the trunk abilities of the patient.[64] It contains 4 items, with item scores ranging from 0 to 25. The sitting balance item assesses the patients' ability to sit during 30 seconds without trunk and feet support and has a predictive value for recovery of walking poststroke.

Action Research Arm Test (ARAT): In four subtests, the ARAT assesses the patients` ability to grasp (subscale with 6 items), grip (subscale with 4 items), pinch (subscale with 6 items) and perform gross movements (subscale with 3 items) with the upper extremity.[65] It assesses the patients` ability to handle objects which are differing in size, weight and shape and is therefore an arm-specific measure of the activity limitation or improvement thereof.[66] The ARAT has been demonstrated to have excellent test-retest reliability,[66] as well as excellent inter-/intrarater reliability in acute[67] and chronic stroke.[68]

Box- and Block Test (BBT): this test assesses the unilateral gross manual dexterity. Patients are seated in front of a square box with two compartments. They are asked to move as many wooden cubes as possible from one compartment to the other within 60 seconds of testing time. Normative data in adults exist[69] and the test has been applied in acute and chronic stroke patients. The BBT has been attributed excellent test-retest and interrater/intrarater reliability. In ESTREL, the BBT will be implemented to specifically determine improvement in functionally relevant actions of reaching and grasping and improvement of fine motor skills of the affected upper extremity.

The Functional Ambulation Categories (FAC) is a classification (score range 0 to 5) regarding the ability to walk independently, with or without a walking aid and takes the type of walking surface into account.[70-72]

The Ten-Meter Walk Test (10MWT) assesses walking speed and cadence over a 10 meter track at both a comfortable and a maximum speed.[70, 73]

The Jamar dynamometer testing (JDT) is a quantitative and objective measure of grip strength and strength of the forearm. This testing has been attributed excellent test-retest reproducibility as well as high inter-rater reliability. Normative data are available in older subjects. Interestingly, Yi et al. recently identified grip strength on the unaffected side as an independent predictor of functional improvement after stroke.[74] In ESTREL, participants will be tested for the affected as well as the unaffected upper extremity.

The Montreal Cognitive Assessment (MoCA) is a screening instrument for mild cognitive impairment (score range 0 to 30).[75]

Daily life physical activity engagement and upper limb use will be measured with movement sensors. The sensors allow assessment of physical activity engagement and upper limb use in daily life situations without physically hampering the patient in the performance of their daily activities.[76] Raw sensor data (gyroscope data, acceleration, magnetometer data and pressure sensor data), activity counts (arm

usage) and physical activity (lay, sit, stand walk) will be assessed.

5.2.3 Further assessments (selected centers only)

Genetic markers, blood biomarkers: Blood sampling will be performed at baseline or any follow-up visit. Exploratory analyses will allow to generate hypotheses about differences in treatment response across participants associated to genetic markers or blood biomarkers.

Imaging biomarkers: No imaging will be performed within the ESTREL study, however, brain imaging (Computed Tomography, (functional) Magnetic Resonance Imaging) obtained in clinical routine or observational studies will be analyzed to identify markers potentially influencing treatment response (e.g. ischemic or hemorrhagic stroke lesion type, lesion location and volume).

5.3 Safety Outcomes

See section 5.2.1.

6. STUDY DESIGN

6.1 General study design and justification of design

ESTREL is a multicenter, randomized (ratio 1:1) parallel-group, placebo-controlled superiority trial with blinded patients, care-providers, investigators, and outcome assessors. The main hypothesis of the trial is, that Levodopa with standardized rehabilitative therapy over 5 weeks starting soon after acute stroke will enhance recovery of motor deficits to a patient-relevant extent, i.e.: Levodopa administered in addition to standardized rehabilitative therapy (adapted to principles of motor learning) is superior to placebo and standardized rehabilitative therapy, resulting in an at least 6 points higher FMMA score at 3 months.

Overall, enrolment of 610 patients is planned. Participating study centers are certified stroke units or stroke centers as well as rehabilitation centers with expertise in stroke patient rehabilitation. The design of this study was assessed using the PRagmatic Explanatory Continuum Indicator Summary (PRECIS – www.precis-2.org). Scores (see Figure 2 below) were entered by consensus reading of the Principal Investigator and the Medical Network Advisor.

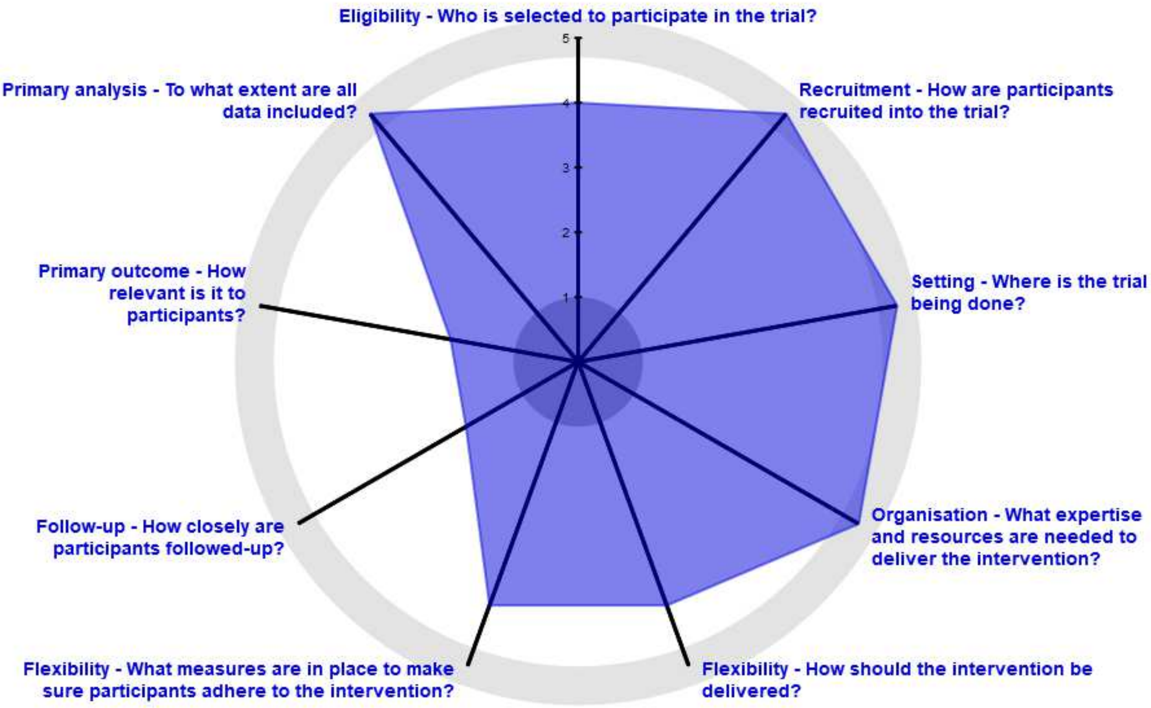

Figure 2: ESTREL scoring according to PRECIS (www.precis-2.org).

Eligibility criteria are outlined in the STUDY SYNOPSIS (see above, section Inclusion and Exclusion criteria). Patients will be screened for eligibility in the acute stroke care center. Consenting and inclusion of the patients will be performed after the patients entered the ward (e.g. IMC/Stroke Unit, neurology ward). No emergency inclusion will be performed. Eligible patient who signed the informed consent will be randomized (1:1 ratio) to either receive Levodopa/Carbidopa or matching placebo three times daily. Study treatment will comprise three phases: 1) *Dose escalation phase*: On day 1-3, patients will receive levodopa/placebo solely in the morning; on day 4-6 in the morning and at lunch time; 2) *full study treatment phase:* from day 7 to day 34, 3 times per day (tid). Treatment will stop with a *tapering phase:* On day 35-37, patients will receive levodopa/placebo in the morning and at lunch time; on day 38 and 39 solely in the morning. All patients in both comparison groups will have standardized rehabilitative therapies. Enrollment, randomization, treatment allocation and treatment onset will be done in the acute stroke unit and transferal to the rehabilitation center will happen thereafter.

For each participant the study participation will comprise two phases with a total duration of 12 months: **(1) Interventional phase** (3 months, i.e. randomization to Visit 3) and **(2) the observational phase** (9 months, i.e. after Visit 3 to Visit 5 at 12 months after randomization). The main intervention in ESTREL will end 5 weeks post-randomization. According to assumed pharmacokinetics (see appendix III) of the IMP multiples of the IMP half-life and wash-out periods will have been reached at Visit 3 (3 months post-randomization) thus justifying the end of the interventional phase at this time point.
Three in-person visits will take place during the interventional phase: Visit 1 (Baseline), Visit 2 (End of treatment), and Visit 3 (primary outcome assessment).
Taking into account recent consensus recommendations on the design of stroke recovery trials[35] participants in ESTREL will be followed up in a purely observational study phase with two in-person long-term follow up visits at 6 months and at 12 months (Visit 4 and 5). The observational phase will clarify if the initial intervention will have a long-lasting patient-relevant effect.
Outcomes as outlined in the STUDY SCHEDULE (see above) will be assessed. Assessment of safety outcomes and (serious) adverse events will be done as described in section 5.2.1 of the study protocol. Additional information (e.g. on number and duration of therapy sessions, and information on patient transfer from acute to rehabilitation center) will be collected at the transition of the patient from the acute care center to rehabilitation center.

6.2 Methods of minimising bias

At Visit 1, patients will be randomly assigned either to the active treatment or the control group. Stratification will be performed by center. The randomization procedure will be implemented by the Clinical Trial Unit of the University Hospital Basel into the Clinical Data Management Application (CDMA) secuTrial®. It will include a standard minimization algorithm which will ensure that the treatment groups are balanced within each stratum. To avoid predictable alternation of treatment allocation, and thus potential loss of allocation concealment, patients will be allocated with a probability of 80 percent to the treatment group that would minimize the difference between the groups within the patient's stratum.

6.2.1 Blinding procedures

All investigators, study participants, care providers (i.e., therapists, physicians, nurses), outcome assessors and the study statistician will remain blinded with respect to the randomization throughout the trial. The study medication is labeled, packed and dispensed by an independent distributor (Bichsel pharmacy) according to the results of the randomization procedure. Placebo and IMP will be indistinguishable (i.e. identical in aspect, texture, and taste) except for the blinded randomization number. The DSMC monitoring trial safety will not be blind to treatment assignment.

6.2.2 Other methods of minimising bias

All outcome assessments (primary, secondary will be performed by trained - for the specific assessment instruments - study personnel only). The use of established, standardized and validated outcome assessment tools will help to minimize any bias in outcome assessment.

6.3 Unblinding Procedures (Code break)

Maintaining the blinding of patients and investigators is crucial, however situations might occur, when information about the patient`s study treatment is mandatory to ensure patient safety and medical management of the patient. The local principal investigators of the study or the treating physician might ask for unblinding by contacting the leading center (Principal Investigator, Medical Network Advisor or their designees). Unblinding can be performed by authorized investigators only, using the CDMA secuTrial ®. Each unblinding is documented in the CDMAs` integrated audit trail system (see section 12.2.).

7. STUDY POPULATION

ESTREL will include patients with acute stroke(≤ 7 days since stroke symptom onset) with clinically meaningful hemiparesis and therefore requiring in-hospital rehabilitation.
Overall, recruitment of 610 patients treated at certified acute stroke care units/ centers in Switzerland is planned. During the study treatment period (day 1 to day 39) patients will transition to Rehabilitation centers with expertise in stroke patient rehabilitation. Additional acute care centers as well as rehabilitation centers may be recruited as ESTREL study centers if patient recruitment goals are not met.

7.1 Eligibility criteria

To be eligible for participation in the present study, each patient must fulfill all of the inclusion criteria and none of the exclusion criteria as outlined in the STUDY SYNOPSIS. Under no circumstances can there be exceptions to this rule.

Full Inclusion and Exclusion Criteria are given in the STUDY SYNOPSIS above (section Inclusion / Exclusion criteria).

7.2 Recruitment and pre-screening

Local Investigators or their delegates at each participating study center will pre-screen potential study participants in the acute stroke phase (Pre-Screening, i.e. day 0-7 since stroke symptom onset). The local Investigator will determine whether a patient is suitable to participate in the present study based on the aforementioned inclusion and exclusion criteria, inform the potential participant (and/or a next-

of-kin) and ultimately will obtain signed informed consent from the patient and/or a next-of-kin before any study specific procedures are performed. Consenting and inclusion of the patients will be performed after the patients entered the ward (e.g. IMC/Stroke Unit, neurology ward). No emergency inclusion will be performed. No advertisement will be made for the study. No payment or compensation will be given to Study Participants. However, participants are eligible for a refund of travel costs if study visits are scheduled outside of routine inpatient or outpatient follow-ups. For administrative reasons this refund will be a flat-rate payment of 20,- CHF per patient / per visit.

7.3 Assignment to study groups

After patients who met the eligibility criteria and have given their written informed consent or written informed consent by a next-of-kin to participate in the present study, the investigator will enrol the patient in the study (Visit 1). Patients will be randomized as described in section 6.2. of the study protocol. Randomization will be done instantly at inclusion of each patient in the CDMA system (computer-generated).
In general, enrolment of patients into the study will be done by local investigators who are part of the local stroke care team and who have been trained on the proposed study during site initiation visit or thereafter. The assignment of patients to the study treatment is done through the randomization procedure in secuTrial®, as described above.

7.4 Criteria for withdrawal / discontinuation of participants

A reduction of the dosage of the study drug to 1-1-0 per day (i.e. one dose in the morning, one at noon, none in the evening) is possible if participants experience adverse reactions at night time. If a participant recovers faster than expected leading to an early discharge (i.e. earlier than 5 weeks minus 7 days after randomization), he/she will be encouraged to continue study medication as planned until the scheduled date of the 5-week-Visit (Visit 2, end of treatment). Participants are likely to continue concomitant rehabilitative therapies in an outpatient setting, thus ongoing effects on neuroplasticity of both rehabilitative therapies as well as the study intervention might be expected in these patients as well. If the participant does not agree to continue the study medication as foreseen in the protocol, the full study treatment will be stopped (discontinuation) followed by a tapering phase during the following 5 days (i.e. study drugs for the last 5 days will be handed to the participant).

An unscheduled visit (end of study) will be performed in case of withdrawal or discontinuation (day of discontinuation/withdrawal +/- 3 days).

In case of Adverse events (AEs), Serious Adverse Events (SAE) or Suspected Unexpected Serious Adverse Reactions (SUSARs) - if relationship to study drug is at least probable – dosage of study medication will be reduced, or study medication will be discontinued temporarily or permanently, whatever is medically appropriate and in agreement with the wish of the patient. In addition, the patient will not continue to receive the study drug if (i) he/she develops contraindications to levodopa (according to appendix III), or (ii) if the treating physician considers the open application of levodopa mandatory, or (iii) if the treating physician deems that the patient is at a significant health risk from continued participation in the trial.

Should during the study drug treatment phase conditions occur which can be treated with Levodopa, (e.g. Restless Legs Syndrome) attending physicians will be asked to prefer non-dopaminergic agents for treatment wherever possible according to treatment guidelines. (as long as the patient is in the main period, i.e. before Visit 3).

Description of follow-up procedures in participants withdrawn from the study treatment or who withdraw their informed consent are given in section 9.1.3 of the study protocol.

8. STUDY INTERVENTION

8.1 Identity of Investigational Products (treatment / medical device)

8.1.1 Experimental Intervention

The IMP in the present study is Levodopa 100mg/Carbidopa 25mg as described in section 3.2 of the study protocol. The IMP will be administered orally from day 1 to day 39 after Visit 1. As described in section 6.1 study drug treatment is administered in three phases (dose escalation phase, full dose phase, tapering phase). Administration of the study drug (Levodopa/Carbidopa or Placebo) via a nasogastric tube (NG) or a percutaneous endoscopic gastrostomy feeding tube (PEG) is allowed in patients unable to swallow study drug capsules. However, such application should be limited to the minimum time frame possible and should be documented as such in the participants medical records as well as the CDMA.
Information on the rationale of the dose, regimen and the rationale for the timing of the study in the course of stroke recovery are given in section 3.1 and 3.5.

8.1.2 Control Intervention

There is no established active comparator to the IMP mentioned in 8.1.1, therefore the comparator used in ESTREL will be matching placebo, identical in aspect, texture, and taste when compared to the IMP. Procedures regarding route of administration, study treatment duration and treatment phases will be identical in the IMP- and the placebo-group (see section 8.1.1).

8.1.3 Packaging, Labelling and Supply (re-supply)

The IMP and the placebo will be capsules identical in aspect, texture and taste. These capsules will be packed in a brown pulvis box. Shelf life is expected to be 12 months. Timely supply of blinded and randomized boxes is guaranteed by Dr. G. Bichsel`s validated distribution channels. Labelling will be as described in section 3.2 of the study protocol.

8.1.4 Storage Conditions

The study medication will be stored at each site at room temperature (range 15–25°C), without exposure to light. Access to the storage area is secured and limited to study personnel.

8.2 Dose / Device modifications

Criteria for the dose modification, temporary or permanent discontinuation are given in section 7.4 of the study protocol.

8.3 Compliance with study intervention

To maximize study drug adherence, the study drug will be administered to the participant by the local study-site nurses. It is expected that all participants in the study will stay hospitalized (acute care and/or rehabilitation center) during the whole study treatment phase, thus participating sites will be able to closely monitor the participants for correct study drug intake and occurrence of adverse events. In case of an unforeseen discharge before Visit 2 (End of treatment), participants will be encouraged to continue study medication after discharge according to the protocol (see section 7.4). In such cases, the study drug will be handed to the participant alongside with a medication plan explaining the intake of the study drug until the scheduled end of treatment visit.
Study drugs (IMP or placebo) will be numbered for the complete study period (i.e. total of 39 days of study drug administration). At Visit 2 the number of returned study drug (IMP or placebo) capsules will be documented.

8.4 Data Collection and Follow-up for withdrawn participants

Procedures for Participants withdrawing from the study (withdrawal of consent) are specified in section 7.4. Any study data collected up to the time of withdrawal from the study will be analyzed.

8.5 Trial specific preventive measures

As described in section 7.4: if conditions occur which might be treated with Levodopa, treating physicians will be asked to preferably use non-dopaminergic alternatives if this is in line with according treatment guidelines.
A standard urine or blood pregnancy test must be obtained in female study participants of childbearing potential before study enrolment. If enrolled, female participants of childbearing potential will be asked

to use contraception (such as oral, injectable, or implantable contraceptives, or intrauterine contraceptive devices) during the study treatment period (day 1-39 after Visit 1) and during one month thereafter.

8.6 Concomitant Interventions (treatments)

The off-label use of any medical product which aims at the enhancement of stroke rehabilitation and participant recovery is prohibited until the 3-month visit (primary outcome assessment).
Concomitant medication will be assessed at each study visit and will be entered in the electronic case report form (eCRF) in the following categories:

- Antiplatelets
- Anticoagulants
- Antihypertensives
- Statins
- Serotonin-Reuptake Inhibitors
- Serotonin-Noradrenalin Reuptake Inhibitors
- Benzodiazepines
- Neuroleptic drugs
- Antiepileptic drugs
- Antidiabetic drugs
- Analgesics
- Immunosuppressive / immunomodulating drugs
- Antibiotics

All patients participating in ESTREL, irrespective of the treatment group, should receive the same standardized rehabilitative therapy that is based on principles of motor learning. The characterization of this program and its implementation is described as follows.
(1) The standardized therapy program will take into account the putative advantages of the modulating effects of dopamine on motor cortex plasticity, action learning, and control of voluntary movements. A review of the evidence–based knowledge about motor rehabilitative therapy shows, that there is no single motor rehabilitation program fitting all needs of motor problems in stroke patients.
(2) Nevertheless, there are the following crucial elements determining the efficacy of rehabilitative therapy based on the motor learning theory, which will be considered as principles of the ESTREL -rehabilitation program.

(i) exercises – i.e. active therapy with voluntary, specific, task-related movements initiated by the patients, while passive therapy (such as massage etc.) are not meant.
(ii) tailored exercise adapted to the individual deficits and its improvement over time, i.e., exercises are progressive, maintaining a challenging nature as the patient improves
(iii) feedback on the performance of the movements to be learned (knowledge of performance)
(iv) repetitions with periods of rest
(v) motivation addressed by setting goals, coaching how to reach the goal, and (positive) feedback

Each patient should receive 3 sessions of rehabilitative care per workday (i.e. physical therapy, occupational therapy, speech of swallowing therapy, and neuropsychological training if required). At least two sessions of this training should be standardized motor rehabilitative therapy, provided by physical and/or occupational therapists experienced in applying therapy according to the principles of motor learning and who have been trained in ESTREL-motor rehabilitative program.
The amount (in minutes/hours) and type of rehabilitative therapies received by each participant will be recorded. In the early phase (i.e. on the monitored stroke unit) shorter but more frequent sessions should be preferred – an approach that has been shown to increase the odds of a favorable outcome after stroke.

Participation in the study does not prohibit patients from any medically required intervention, if necessary. As described in section 7.4 of the protocol, if conditions occur which might be treated with Levodopa, treating physicians will be asked to preferably use non-dopaminergic alternatives, if this is in line with according treatment guidelines. However, if during the study, the use of levodopa is mandatory in the view of the treating physicians, study medication will be discontinued. In such cases, patients stay in ESTREL as "off-study-medication-patients".
As it is clinical practice (also outside studies), concomitant medications can be used if required, taking

account interactions with Levodopa as mentioned in the appendix III.

8.7 Study Drug / Medical Device Accountability

The study drug will be provided to the acute care centers by Bichsel Pharma as described in section 8.1.3 of the study protocol. Each study site will keep detailed record of the study drug received (number of study drug packages as well as check and confirmation of lot/batch number and label, date received by supplier). Drug accountability will be performed as described in section 8.8.

8.8 Return or Destruction of Study Drug

Drug distribution will be documented in a log. At Visit 2, all study drugs and packaging thereof will be returned to the study personnel. At this visit, the study personnel will count the number of study drug capsules that have been returned and will enter this information in the eCRF. The returned study drug packaging and study drug capsules will be stored (separately from unused study drug) at the site or the leading center with the packaging clearly marked as used and returned medication. After completion of on-site monitoring returned study drug will be sent to the Bichsel Pharmacy, Interlaken where the drugs will be destroyed.

9. STUDY ASSESSMENTS

A study flow chart depicting the course of the study is given above.

All collected demographical and clinical data will be entered into the CDMA as described in Section 12.1.1. of the study protocol. In pre-defined centers blood samples (approximately 10 ml of whole blood in EDTA tubes) will be taken for secondary outcome assessment. These blood samples will be stored (-20°C freezer) at each local site and will ultimately be collected by delegates of the leading center Basel upon final outcome analyses.

9.1 Assessments of outcomes

9.1.1 Assessment of primary outcome

The primary outcome is the between-group difference of scores in the FMMA[48] measured 3 months (+/- 14 days) after randomization (Visit 3).
The assessment of the FMMA will be performed by trained study personnel only (i.e. trained study investigators, ESTREL study core therapist or trained local therapists at each center). Any FMMA assessor within the study will have to provide certification of FMMA training to be eligible for FMMA assessments within the ESTREL study. FMMA scores will be entered in the CRF at the study visit. The FMMA assessment at 3 months will also be filmed. The recorded video will be stored and provided to the leading center Basel for a central primary outcome rating by an assessor blinded to study treatment. All personnel involved in primary outcome assessment will be blinded to the allocated study treatment.

9.1.2 Assessment of secondary outcomes

A detailed description of the secondary outcomes as well as information on timepoints of each assessment is provided in section 5.2 of the study protocol as well as the STUDY SCHEDULE.

The assessment of the secondary outcomes will be performed by trained study personnel only (i.e. trained study investigators, the ESTREL study core therapist or trained local therapists at each center). As described in section 9.1.1 all outcome assessors will have to provide certification of training in the assessment of the various study outcomes.

9.1.2.1 Adverse events and measures of harm

Mortality (all cause), recurrent stroke (any) as well as all SAEs and non-serious, pre-specified AEs (see section 10.1.1) possibly related to the IMP occurring between Visit 1 and Visit 3 will be recorded by local study personnel at each center during the interventional phase of the study. Mortality (all cause) and recurrent stroke (any) will be recorded by study personnel at each center during the observational study phase (3 to 12 months post randomization). Definitions of Adverse and Serious Adverse events are provided in section 10 of the study protocol.

9.1.3 Assessments in participants who prematurely stop the study

Criteria and procedures for withdrawal from the study are provided in section 7.4 of the study protocol. Study data collected up to the time of study withdrawal may be used for further analyses.

9.2 Procedures at each visit during the Interventional Study Phase

9.2.1 Pre-screening (day -7 to day 0 before randomization)

Potentially eligible patients treated at the stroke unit or stroke centers of the participating centers will be pre-screened for potential study participation. Taking into account potential rapid and early recovery (e.g. after thrombectomy or thrombolysis), a latency period of 24h after thrombolysis and/or thrombectomy has to be respected before pre-screening and enrolment as a meaningful hemiparesis (i.e., NIHSS ≥ 3 for arm plus leg motor functions) has to be present to fulfil the inclusion criteria. Baseline demographic data will be assessed from routine clinical patient charts. If eligible, potential participants and/or their next-of-kin will be informed about the study by the local study investigator. Potential participants will be handed a patient informed consent form.

9.2.2 Visit 1: (day 0)

At Visit 1 participants and / or their next-of-kin will give their written informed consent to participate in the study. The participant will be screened according to the in- and exclusion criteria. Final eligibility will be confirmed. The investigator will then obtain baseline demographic data, medical history, will perform a physical examination and will note the current concomitant medication. At the day of randomization and prior to treatment allocation the following baseline assessments will have to be performed (mandatory): FMMA, NIHSS, mRS, RMI.

In selected centers only the ARAT, BBT, JDT, MI, TCT, FAC, 10MWT, MoCa and assessment of daily activity with movement sensors will be performed.

At day 0 the investigator will initiate randomization via the eCRF. The study participant will be assigned to the study treatment. The local investigator will recognize and document the assigned study drug by matching description and label of the assigned study drug treatment in the eCRF and the study medication supplied to each center.

Study treatment will start at the day after randomization (day 1) (see section 9.2.3 below).

Enrolment, randomization, treatment allocation and treatment onset will be done in the acute stroke unit and transferal to the rehabilitation center will happen thereafter.

9.2.3 Study treatment: Run-in, full-dose treatment, tapering phase (day 1-39)

Participants will be randomized to receive either Levodopa 100mg/Carbidopa 25mg or matching placebo (ratio 1:1) beginning from day 1 after randomization until day 39. Treatment will start with a dose escalation phase: On day 1-3, participants will receive levodopa/placebo solely in the morning; on day 4-6 in the morning and at lunch time; from day 7 to day 34, 3 times per day (tid). Treatment will stop after a tapering phase: On day 35-37, participants will receive levodopa/placebo in the morning and at lunch time; on day 38 and 39 solely in the morning. All participants in both comparison groups will have standardized concomitant rehabilitative therapies (details given in section 8.6 of the study protocol).

Please see section 9.4for additional scheduled or unscheduled visits during the study treatment phase.

9.2.4 Visit 2: (5 weeks +/- 7 days)

Five weeks (+/- 7 days) after randomization, Visit 2 (End of treatment, in-person visit) will be performed. The investigator will perform a physical examination and will note the current concomitant medication as well as the number, the length and the type (physiotherapy, occupational therapy, therapy according to principles of motor learning, other therapies) of the concomitant therapies during study treatment phase. Outcome measures will be obtained as outlined in the STUDY SCHEDULE as well as section 5 of the protocol. The following measures of harm will be assessed: any non-serious, pre-specified adverse event possibly related to the IMP or SAE, recurrent stroke (any), mortality (all cause).

Study drugs and study drug packaging will be returned to the study site at this visit. Remaining study drug capsules (if any) will be counted and the number of returned capsules will be entered in a log and the eCRF.

9.2.5 Visit 3: (3 months +/- 14 days)

Three months (+/- 14 days) after randomization, Visit 3 (primary outcome assessment, in-person visit)

will be performed. The investigator will perform a physical examination and will note the current concomitant medication as well as concomitant therapies during post-study treatment phase. Outcome measures will be obtained as outlined in the STUDY SCHEDULE as well as section 5 of the protocol. The following measures of harm will be assessed: any non-serious, pre-specified adverse event possibly related to the IMP or SAE, recurrent stroke (any), mortality (all cause).

9.3 Procedures at each visit during the Observational Study Phase

9.3.1 Visit 4: 6-months long-term follow-up (6 months +/- 20 days)

Six months (+/- 20 days) after randomization, Visit 4 (first long-term follow-up visit, in-person visit) will be performed. The investigator will perform a physical examination and will note the current concomitant medication as well as concomitant therapies during post-study treatment phase. Outcome measures will be obtained as outlined in the STUDY SCHEDULE as well as section 5 of the protocol. The following measures of harm will be assessed: recurrent stroke, mortality (all cause).

9.3.2 Visit 5: 12-months long-term follow-up (12 months +/- 30 days)

Twelve months (+/- 30 days) after randomization, Visit 5 (second long-term follow-up visit, in-person visit) will be performed. The course and the performed procedures at Visit 5 are identical to Visit 4 (please see section 9.3.1 above).

9.4 Additional data collection and visits

Transition
Enrollment, randomization, treatment allocation and treatment onset will be done in the acute stroke unit and transferal to the rehabilitation center will happen thereafter. At the day of the transition of the patient from the acute care center to the rehabilitation center a transition data assessment (not in-person) will be performed. This includes: a) assessment of concomitant therapies during the study treatment at the acute care center (i.e. the number, the length and the type (physiotherapy, occupational therapy, therapy according to principles of motor learning, other therapies) of the concomitant therapies), b) ensuring, that study drug is transferred together with the patient from acute care to rehabilitation center, c) assessment of measures of harm since randomization (AE/SAE, mortality (all cause), recurrent stroke (any)), d) entering the name of the rehabilitation center to which the participant is transferred.

Unscheduled (end of study): will be performed as described in the STUDY SCHEDULE and in section 7.4 of the protocol.

10. SAFETY

10.1 Safety Interventional Study Phase

Mortality (all cause), recurrent stroke (any) and all serious adverse events (SAEs) and non-serious pre-specified adverse events are collected, fully investigated and documented in source documents and case report forms (CRF) up to 3 months (i.e. Visit 3) after study enrolment.

10.1.1 (Serious) Adverse events and other safety related events

10.1.2 Definition and assessment of (serious) adverse events and other safety related events

An **Adverse Event (AE)** is any untoward medical occurrence in a patient or a clinical investigation participant administered a pharmaceutical product, and which does not necessarily have a causal relationship with the study procedure. An AE can therefore be any unfavourable and unintended sign (including an abnormal laboratory finding), symptom, or disease temporally associated with the use of a medicinal (investigational) product, whether or not related to the medicinal (investigational) product. [ICH E6 1.2]

The following pre-specified AEs of interest will be assessed in ESTREL (see also section 3.7 of the study protocol):
Nausea, Vomiting, Taste disturbances, Dry mouth, Anorexia, Arrhythmias, Postural hypotension Syncope (unconsciousness for a short time as a result of reduced blood flow to the brain), drowsiness

(including sudden onset of sleep) Fatigue, Dementia, Psychoses (a distorted perception of reality), Hallucinations, Confusion, Euphoria, Abnormal dreams, Insomnia, Depression, Anxiety, Dizziness, Dystonia (involuntary contractions), Dyskinesia (inability to control voluntary movements), Chorea (sudden twitching of the face and shoulders).

A **Serious Adverse Event (SAE)** is classified as any untoward medical occurrence that:

- results in death,
- is life-threatening,
- requires in-patient hospitalization or prolongation of existing hospitalisation,
- results in persistent or significant disability/incapacity, or
- is a congenital anomaly/birth defect

In addition, important medical events that may not be immediately life-threatening or result in death, or require hospitalisation, but may jeopardise the patient`s health or may require intervention to prevent one of the other outcomes listed above should also usually be considered serious. [ICH E2A]
SAEs will be followed until resolution or stabilisation. Participants with ongoing SAEs at the end of the interventional study phase (including safety visit) will be further followed up until recovery or until stabilisation of the disease after termination.

Assessment of Causality
Both, the local Investigator and the Sponsor-investigator make a causality assessment of the event to the study drug, based on the criteria listed in the ICH E2A guidelines[77]:

Relationship	Description
Definitely	Temporal relationship<br>Improvement after dechallenge*<br>Recurrence after rechallenge<br>(or other proof of drug cause)
Probably	Temporal relationship<br>Improvement after dechallenge<br>No other cause evident
Possibly	Temporal relationship<br>Other cause possible
Unlikely	Any assessable reaction that does not fulfil the above conditions
Not related	Causal relationship can be ruled out
*Improvement after dechallenge only taken into consideration, if applicable to reaction	

Unexpected Adverse Drug Reaction
An “unexpected” adverse drug reaction is an adverse reaction, the nature or severity of which is not consistent with the applicable product information (see appendix III for detailed information on IMP).[78]

Suspected Unexpected Serious Adverse Reactions (SUSARs)
The Sponsor-Investigator evaluates any SAE that has been reported regarding seriousness, causality and expectedness. If the event is related to the investigational product and is both serious and unexpected, it is classified as a SUSAR.

Assessment of Severity
Severity of any AEs will be graded by the local Investigator as well as the Sponsor-Investigator based on the following criteria according to the Common Terminology Criteria for Adverse Events Version 5 published November 27, 2017:

Severity	Description
Grade 1	Mild; asymptomatic or mild symptoms; clinical or diagnostic<br>observations only; intervention not indicated.
Grade 2	Moderate; minimal, local or noninvasive intervention indicated;<br>limiting age-appropriate instrumental ADL.
Grade 3	Severe or medically significant but not immediately life-threatening;<br>hospitalization or prolongation of hospitalization<br>indicated; disabling; limiting self care ADL.
Grade 4	Life-threatening consequences; urgent intervention indicated.
Grade 5	Death related to AE.

A Semi-colon indicates 'or' within the description of the grade. ADL: activities of daily living.

10.1.3 Reporting of serious adverse events (SAE) and other safety related events

Reporting of SAEs

All SAEs must be reported immediately and within a maximum of 24 hours to the Sponsor-Investigator of the study. The Sponsor-Investigator will re-evaluate the SAE and return the form to the site. SAEs resulting in death are reported to the lead CEC via BASEC within 7 days. The other in the trial involved Ethics Committees receive SAEs resulting in death in Switzerland via Sponsor-Investigator via BASEC within 7 days.

Reporting of SUSARs

A SUSAR needs to be reported to the Ethics Committee (local event via local Investigator) via BASEC and to Swissmedic for category B and C studies (via Sponsor-Investigator) within 7 days, if the event is fatal, or within 15 days (all other events). The Sponsor-Investigator must inform all Investigators participating in the clinical study of the occurrence of a SUSAR. The CEC will be informed about SUSARs in via Sponsor-Investigator via BASEC according to the same timelines.

Reporting of Safety Signals

All suspected new risks and relevant new aspects of known adverse reactions that require safety-related measures, i.e. so-called safety signals, must be reported to the Sponsor-Investigator within 24 hours. The Sponsor-Investigator must report the safety signals within 7 days to the CEC (local event via local Investigator) via BASEC and to Swissmedic.

The Sponsor-Investigator must immediately inform all participating Investigators about all safety signals.

Reporting and Handling of Pregnancies

Pregnancy, breast-feeding and lack of safe contraception are exclusion criteria for participation in the study. Pregnant participants must immediately be withdrawn from the clinical study. Any pregnancy during the treatment phase of the study and within 30 days after discontinuation of study medication will be reported to the Sponsor-Investigator within 24 hours. The course and outcome of the pregnancy should be followed up carefully, and any abnormal outcome regarding the mother or the child should be documented and reported.

Periodic reporting of safety

The Sponsor-Investigator will submit an annual safety report once a year to the CA and CEC. The annual safety report contains information from all sites. The Sponsor-Investigator prepares the report, and then submits it to the CA and CEC.

10.1.4 Follow up of (Serious) Adverse Events

Any SAEs and non-serious, pre-specified adverse events possibly related to the IMP of interest that are unresolved at the time of the Participant's Visit 3 in the study are followed up by the local Investigator for as long as medically indicated, but without further recording in the EDCS. The Sponsor-Investigator retains the right to request additional information for any Participant with ongoing AE(s) of interest /SAE(s) at the end of the study, if judged necessary.

The following variables will be collected and recorded for each non-serious AE of interest and AE leading to discontinuation of study medication:

- AE description (verbatim)
- The date when the AE started and stopped
- Maximum intensity (grading)
- Whether the AE is serious or not
- Investigator causality rating against the study medication (yes or no)
- Frequency (single episode, intermittent, continuous)
- Action taken with regard to study medication
- Outcome

In addition, the following variables will be collected for SAEs:

- SAE description (verbatim) and evaluation of event (severity, outcome, expected vs unexpected)
- Date AE met criteria for serious AE
- Date Investigator became aware of serious AE

- Seriousness criteria present
- Date of hospitalization
- Date of discharge
- Probable cause of death
- Date of death
- Autopsy report (if performed)
- Autopsy results
- Causality assessment in relation to Study procedure(s)
- Causality assessment in relation to other medication
- Concomitant drug and medical history at timepoint of SAE

10.2 Safety Observational Study Phase

Mortality (all cause), recurrent stroke (any) and Serious events (SEs) according to HRO Art. 20 collected, fully investigated and documented in source documents and case report forms (CRF) during the observational study phase (i.e. 3 to 12 months after randomization).

10.2.1 Notification of safety and protective measures (HRO Art. 20)

The Sponsor is promptly notified (within 24 hours) if immediate safety and protective measures have to be taken during the conduct of the research project. The Ethics Committee will be notified via BASEC of these measures and of the circumstances necessitating them within 7 days.

10.2.2 Serious events (HRO Art. 21)

According to HRO Art. 21 a Serious event is defined as: " (…) any adverse event where it cannot be excluded that the event is attributable to the sampling of biological material or the collection of health-related personal data, and which: a) requires inpatient treatment not envisaged in the protocol or extends a current hospital stay; b) results in permanent or significant incapacity or disability; or c) is life-threatening or results in death." (HRO Art. 21)

If a serious event occurs, the research project will be interrupted and the Ethics Committee notified on the circumstances via BASEC within 7 days according to HRO Art. 21.

11. STATISTICAL METHODS

11.1 Hypothesis

Primary hypothesis: Levodopa with standardized rehabilitative therapy over 5 weeks starting soon after acute stroke will enhance recovery of motor deficits to a patient-relevant extent, i.e.: Levodopa administered in addition to standardized rehabilitative therapy is superior to placebo and standardized rehabilitative therapy, resulting in an at least 6 points higher FMMA score at 3 months.

11.2 Determination of Sample Size

The sample size estimate is based on the primary hypothesis, i.e. this trial is powered to be able to detect a difference in the FMMA score of 6 points at 3 months.
There is no information on FMMA from the DARS-trial, therefore information on distribution of FMMA measurements from the large FLAME-trial[4] was used which was conducted with the same intention as ESTREL, i.e. to evaluate a pharmacologically enhanced motor recovery (albeit using another drug treatment), and in a similar setting and target population as ESTREL (patients with ischemic stroke and moderate to severe motor deficit, treated in nine stroke units in France). Based on the FLAME-trial, the FMMA was assumed to be normally distributed with a standard deviation of 25 points, which is slightly more conservative than the observed SD in the placebo group (mean FMMA score in the placebo arm of the FLAME-trial: 35.1 points (22 SD) at day 90 after randomization). The intra-patient correlation of the FMMA score between baseline and follow-up was assumed to be high. Since there was no reliable information on this correlation in the aforementioned publications, the power gain resulting from the baseline adjustment could not be estimated and a t-test was assumed in the power calculation. The adjustment for the baseline measurement is expected to rather increase power than to decrease it.
Under the aforementioned conditions, assuming that the FMMA is normally distributed with a standard deviation of 25 points, 548 patients should allow to detect a mean difference between the experimental and control group (1:1 allocation of treatments) in the FMMA score of 6 points (what is deemed clinically- and patient-relevant) at 3 months with a power of 80% (two-sided significance

level of 5%).
To allow for 10% drop out, 305 patients will be recruited per arm, i.e., 610 in total. Applying the placebo-data from the FLAME-trial, the treatment effect in ESTREL would result in a mean FMMA score of 41.1 points in the levodopa group compared to 35.1 in the placebo group.

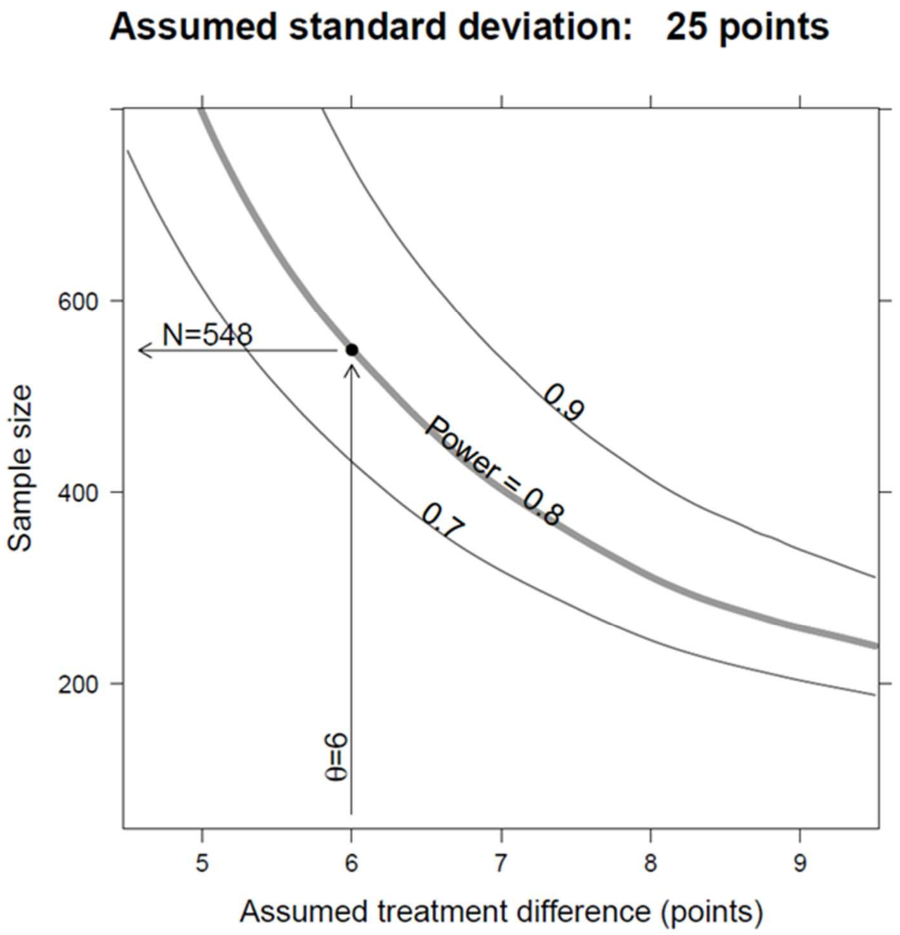

Fig 3: Sensitivity of the sample size with respect to the assumed difference in the Fugl Meyer Score in points due to levopoda. The curves for a power of 0.7, 0.8 and 0.9 (ie. 70 %, 80 % and 90 %) are shown. (The curves are smoothed and are shown for illustration only.)

The trial was also designed to have sufficient power (80%) to detect patient-reported improvements as measured by the novel PROMIS tool at 3 months. However, given the novelty of this measurement tool, there is less empirical data supporting the application in the ESTREL target population and setting, than for the well-established traditional scores. Therefore, the sample size calculation was based primarily on the traditional measures.
This sample size of 610 recruited patients will also provide sufficient information to evaluate the impact of the intervention on the ability to walk (i.e. a RMI score of ≥ 7, as in DARS) and to directly interpret the findings in context of the other large trial in this field (DARS). Assuming that 45% of patients in the placebo group will walk independently at 5 weeks (as in DARS), this given sample size would provide statistical power of 80% (at 5% significance level) to detect a minimum improvement in the active treatment group of 12% more patients walking independently after 5 weeks (i.e. 57% vs. 45%). Given the high prevalence of stroke and its tremendous burden for the patients, this *absolute* increase of 12% or a *relative* increase of 27% more patients walking independently would be highly relevant and would alter clinical practices.

11.3 Statistical criteria of termination of trial

No efficacy stopping rules are foreseen in the trial, however the study may be terminated for safety reasons according to the recommendation of the DSMC which will monitor participants` safety in the study.

The DSMB will monitor the frequency of the following primary composite safety events: i) death of any cause, ii) recurrent stroke, iii) occurrence of Serious Adverse Events. Composite safety risk thresholds above which the DSMC will alert the Sponsor-Investigator will be defined in the DSMC charter taking into account the findings from the DARS trial.

11.4 Planned Analyses

Detailed methodology for summaries and statistical analyses of the data collected in this study will be documented in a statistical analysis plan.

The statistical analysis plan will be finalized before database closure and will be under version control at the Clinical Trial Unit, University Hospital Basel.

11.4.1 Datasets to be analysed, analysis populations

The full analysis set (FAS) consists of all patients who were randomized. According to the intention-to-treat principle, each patient will be analyzed according to the treatment they were randomly allocated to.
The complete cases analysis set will include all patients in the full analysis set without any missing values in the FMMA at baseline or at 3 months.
The per protocol (PP) set consists of patients in the FAS who had at least 80% of the prescribed study treatment (Levodopa / Carbidopa or matching Placebo) or justified reduced dosage described in section 7.4 of the study protocol (reduction to 1-1-0 per day). Furthermore, PP patients will have to attend at least 5 sessions of rehabilitation therapy per week.
All patients will be analyzed according to their randomly allocated treatment. Reasons for withdrawal will be reported for each treatment group and qualitatively compared.

11.4.2 Primary Analysis

Primary outcome: The FMMA at 3 months after onset of treatment is the primary endpoint. It will be analyzed by a mixed effects model, with FMMA-score at baseline as covariate to adjust for differences at baseline and treatment as two-level factor ("levodopa'' vs. "placebo'') as well as type of event (acute ischemic vs hemorrhagic stroke) as fixed effects. Furthermore, for each center a random intercept will be estimated.
The statistical analyses will be done according to the statistical analysis plan (see section 11.4) after study termination and database closure at the CTU Basel. A two-sided significance level of 5% will be applied for the analyses.

11.4.3 Sensitivity Analyses

The following sensitivity analyses will be performed for the primary endpoint (FMMA at 3 months):

a) The heterogeneity of treatment effects across centers will be investigated in an explorative manner by graphical display of the results stratified by center.

b) The main analysis will be repeated including an interaction term between treatment ("levodopa" vs "placebo") and type of index event (acute ischemic vs hemorrhagic stroke) as additional covariate

c) Explorative analyses will be performed including the following covariables:
- Age
- Sex
- Dementia
- presence of selective hand and wrist movement at baseline
- cortical dysfunction (aphasia neglect/hemianopia
- fatigue (self-report by patient)
- presence of Selective Serotonin Reuptake Inhibitor (SSRI) treatment
- mild extrapyramidal motoric symptoms (by judgement of the treating physician),
- reduced impetus (by judgement of the treating physician)

The results will be compared to the results from the main analysis.
d) The main analysis will be repeated on the complete cases analysis set (excluding patients with

imputation of the primary endpoint or the baseline FMMA score) and will be compared to the main analysis.
e) The analysis will be repeated on the per protocol set and compared to the main results of the analysis to investigate the influence of protocol deviations. In case of major protocol deviations, the use of marginal structural models will be considered.
All analyses except analysis (c and d) will be performed on the full analysis set.

11.4.4 Secondary Analyses

Secondary outcomes: The secondary outcomes will be analyzed by regression models appropriate to the data type. The analyses will be adjusted as defined for the main analysis. In particular, baseline measures will be included where available. Binary endpoints will be compared using a logistic regression (generalized linear model with logit link and binomial error distribution). For continuous endpoints, linear models will be used. Ordinal endpoints will be analyzed using ordinal logistic regression. The models will be inspected as described for the main analysis. In case of severe violation of the model assumptions or presence of outliers with high leverage, transformation of the data or the use of other methods such as non-parametric tests or robust regression models will be considered. For each model the estimated treatment difference, the p-value and the 95% confidence interval will be presented. The analysis will be performed using a two-sided significance level of 5%.
Furthermore, all endpoints will be presented descriptively in summary tables and graphs. The analyses will be performed on the full analysis set including all patients that were randomized. According to the intention-to-treat principle, each patient will be analyzed according to the treatment they were randomly allocated to.
Exploratory analyses will be performed to identify genetic markers and other biomarkers (including imaging biomarkers as obtained in routine clinical imaging or fMRI in observational studies) that potentially influence treatment response. As examples, specific gene variants, associated with levodopa metabolism may be tested for association with levodopa treatment response in the context of motor recovery (for instance variant rs4633 in exon 5 or variant rs4680 in exon 6 of the catechol-o-methyl transferase (COMT) gene). Adjustment for such genetic variants may reduce the variation of in outcome and therefore increase the power to detect a significant difference between levodopa and placebo in the current study. Moreover, the ESTREL study would allow a detailed and high-quality investigation or the association between stroke outcome and genomic imbalance. For such a genome wide study of genetic imbalance, DNA of patients will be hybridized on high density SNP microarrays platforms and analyzed for Copy Number Variation.

Participation in future individual patient data meta-analyses with similar studies (e.g. DARS-trial) is intended.
Additional exploratory analyses might be performed triggered by scientifically relevant findings from the study outcomes.

11.4.5 Interim analyses

An interim analysis is not planned.

11.4.6 Safety analysis

Safety will be assessed via rigorous and detailed examination of pre-specified events, adverse events and serious adverse events. The proportion of patients with at least one pre-specified adverse event will be estimated by treatment group. Safety will closely be monitored by the ESTREL DSMC as described in section 1.6.

11.4.7 Deviation(s) from the original statistical plan

Any deviation from the aforementioned analyses will be reported to the CEC and CA. If substantial deviations of the analysis as outlined in these sections are needed for whatever reason, the protocol will be amended. All deviations of the analysis from the protocol or from the detailed analysis plan will be listed and justified in a separate section of the final statistical report.

11.5 Handling of missing data and drop-outs

Careful planning and conduct of the study will minimize the occurrence of missing data as far as possible but we considered up to 10% drop outs in our sample size calculation.

Missing data will be addressed using multiple imputation techniques by chained equations according to Van Buuren et al. (2011).[79] Thereby predictive models will be used to create a set of clinically reasonable imputations. We assess the impact in sensitivity analyses using the complete case approach.

12. QUALITY ASSURANCE AND CONTROL

12.1 Data handling and record keeping / archiving

12.1.1 Electronic Case Report Forms

Data will be recorded with electronic Case Report Forms (eCRF) provided by the CDMA secuTrial®. For each enrolled study participant, a CRF is maintained. A unique patient identifier will be used to identify patients. Site Principal Investigators and, if applicable, delegates at each site will be authorized to do eCRF entries.

12.1.2 Specification of source documents

Source data will include all study documents, like e.g. informed consent forms, laboratory reports, AE/SAE forms, etc. Source data will be available at all sites and may be found in paper or electronic form.

12.1.3 Record keeping / archiving

All study data will be archived for a minimum of 10 years after study termination or premature termination of the clinical trial. Any study relevant source data and documents will be archived at each study site for a minimum of 10 years.

12.2 Data management

Study data will be captured via an online CDMA, secuTrial®, based at the IT-department of the University Hospital Basel. The data collected is entered into the study eCRF. An audit trail will maintain a record of initial entries and any changes made; time and date of entry; and user name of person authorizing entry or change. For each patient enrolled an eCRF must be completed. The principal investigator and Co-Investigator at the study site will be responsible for assuring that the data entered into the eCRF is complete, accurate, and that the entry and updates are performed in timely manner. If a patient withdraws from the study, the reason must be noted on a dropout form of the eCRF.

12.2.1 Data Management System

The eCRF will be implemented by the Datamanagement group at the Clincial Trial Unit (CTU) of the University Hospital Basel using the CDMA secuTrial®. The CDMA runs on a server maintained by the IT-department of the University Hospital Basel. Additional storage capacity can be added as needed. Data entry will be performed by trained clinical investigators.

12.2.2 Data security, access and back-up

The CDMS is accessible via a standard browser on devices with internet connection. Password protection and user-right management ensures that only authorized study investigators, monitors, data managers and local authorities (if necessary) will have access to the data during and after the study. User administration and user training is performed by the CTU Basel according to predefined processes. An integrated audit trail system will maintain a record of initial entries and changes made; time and date of entry; and user name of person authorizing entry or change. Backup of secuTrial® study data is performed regularly according to the processes of the IT-department of the University Hospital Basel.

12.2.3 Analysis and archiving

The CDMA will be locked after eCRF data entry is completed, all data has been monitored and raised queries have been resolved. The complete study dataset is exported from the database and transferred to the study statistician as well as the principal investigator through a secure channel. The statistical analysis will be performed completely independent by the involved statistician of the CTU Basel. The exported data will be archived for 10 years by the principal investigator.

12.2.4 Electronic and central data validation

Data entered into the CDMA will be validated for completeness and discrepancies automatically. The data will be reviewed by the responsible investigator as well as an independent monitor (see section

12.3). The monitor will raise queries using the query management system implemented in secuTrial®. Designated investigators have to respond to the query and confirm or correct the corresponding data. Thereafter the monitor can close the query.

12.3 Monitoring

Regular monitoring visits at each participating site will be performed by the CTU Basel and will be organized in coordination with the Sponsor. The frequency as well as the extent of the monitoring will be specified in a separate, study specific monitoring plan.
All study sites will make requested source data and/or documents accessible to monitors. All issues and questions that might arise during monitoring will be answered by the local Principal Investigators or their designees.

12.4 Audits and Inspections

In accordance with ICH GCP Guidelines audits may be performed by the CEC and CA during the course of the study. The audits will include control of adherence to the protocol, standard operating procedures (SOPs), ICH GCP Guidelines and national law. Source data verification and checking of data entered in the eCRF will be used for assessment of complete and reliable documentation. The local Investigators ensure that source data and documents are made accessible to auditors and inspectors and answer their questions. All involved parties must keep the participant data strictly confidential.

12.5 Confidentiality, Data Protection

All data collected as part of this study are coded by the participant study ID. No personal data will be stored apart from age and sex. For the purposes of site monitoring (see section 12.3.), and audits and inspections (see section 12.4.), Study Monitors or designated staff of the CEC or CA will be granted access to source documents (ICHE6, 6.10). However, all involved parties will keep personal data of study participants strictly confidential.

Study data entered into the eCRF is only accessible by authorized persons. Once all data is entered into the CDMA and monitoring is completed, the database will be locked and closed for further data entry. The complete dataset is then exported and transferred to the study statistician as well as the principal investigator through a secure channel (see section 12.2).

Study results disseminated at conferences or published in medical journals will include summary data of study participants. Under no circumstances will the identity of study participants be revealed. The study protocol will be published in a medical journal with open access to the public in conjunction with the main results of the study.

12.6 Storage of biological material and related health data

For assessments of secondary outcomes (genetic variables and biomarkers) blood samples will be taken from the participants upon patient informed consent (selected centers only) independent from the primary consent to participation in the study. Coded samples will be stored at the participating sites and will be centrally (University Hospital Basel) collected from participating sites. This storage will be done according to the “Biobank reglement of the Stroke Research Group Basel”.

13. PUBLICATION AND DISSEMINATION POLICY

Results of ESTREL will be presented at international stroke and stroke rehabilitation conferences and will be published in an open-access peer-reviewed medical journal. Main results will also be published in the Swiss Medical Forum (Section “Forschung.ch”) to reach a wide range of Swiss physicians involved. The main results will also be presented in a press release to spread the main information to the interested patients, relatives and laypersons. A press release will also be published online via the website of the Felix-Platter-Spital, the University Hospital Basel, the Swiss Stroke Society and the Swiss Society of Neurehabilitation. Patient support groups will be informed on the results of the study via Social Networks.

14. FUNDING AND SUPPORT

14.1 Funding

This study is funded by the Swiss National Science Foundation Grant Number: 33IC30_179667.

15. INSURANCE

Insurance will be provided by the Sponsor. A copy of the certificate is filed in each investigator site file and the trial master file. The certificate will be provided as soon as ethics approval has been obtained.

16. REFERENCES

1. Cramer SC. Drugs to Enhance Motor Recovery After Stroke. Stroke; a journal of cerebral circulation 2015;46:2998-3005.

2. Ricci S, Celani MG, Cantisani AT, Righetti E. Piracetam for acute ischaemic stroke. The Cochrane database of systematic reviews 2006:CD000419.

3. Martinsson L, Hardemark H, Eksborg S. Amphetamines for improving recovery after stroke. The Cochrane database of systematic reviews 2007:CD002090.

4. Chollet F, Tardy J, Albucher JF, et al. Fluoxetine for motor recovery after acute ischaemic stroke (FLAME): a randomised placebo-controlled trial. Lancet neurology 2011;10:123-130.

5. Mead GE, Hsieh CF, Lee R, et al. Selective serotonin reuptake inhibitors (SSRIs) for stroke recovery. The Cochrane database of systematic reviews 2012;11:CD009286.

6. Tritsch NX, Sabatini BL. Dopaminergic modulation of synaptic transmission in cortex and striatum. Neuron 2012;76:33-50.

7. McAllister TW. Polymorphisms in genes modulating the dopamine system: do they inf luence outcome and response to medication after traumatic brain injury? J Head Trauma Rehabil 2009;24:65-68.

8. Abe M, Schambra H, Wassermann EM, Luckenbaugh D, Schweighofer N, Cohen LG. Reward improves long-term retention of a motor memory through induction of offline memory gains. Curr Biol 2011;21:557-562.

9. Salamone JD, Correa M. The mysterious motivational functions of mesolimbic dopamine. Neuron 2012;76:470-485.

10. Salamone JD, Yohn SE, Lopez-Cruz L, San Miguel N, Correa M. Activational and effort-related aspects of motivation: neural mechanisms and implications for psychopathology. Brain : a journal of neurology 2016;139:1325-1347.

11. Costa RM. Plastic corticostriatal circuits for action learning: what's dopamine got to do with it? Ann N Y Acad Sci 2007;1104:172-191.

12. Surmeier DJ, Graves SM, Shen W. Dopaminergic modulation of striatal networks in health and Parkinson's disease. Curr Opin Neurobiol 2014;29:109-117.

13. Floel A, Breitenstein C, Hummel F, et al. Dopaminergic influences on formation of a motor memory. Ann Neurol 2005;58:121-130.

14. Floel A, Hummel F, Breitenstein C, Knecht S, Cohen LG. Dopaminergic effects on encoding of a motor memory in chronic stroke. Neurology 2005;65:472-474.

15. Molina-Luna K, Pekanovic A, Rohrich S, et al. Dopamine in motor cortex is necessary for skill learning and synaptic plasticity. PloS one 2009;4:e7082.

16. McEntee WJ, Mair RG, Langlais PJ. Neurochemical specificity of learning: dopamine and motor learning. Yale J Biol Med 1987;60:187-193.

17. Mohr C, Landis T, Bracha HS, Fathi M, Brugger P. Human locomotion: levodopa keeps you straight. Neurosci Lett 2003;339:115-118.

18. Doyon J. Motor sequence learning and movement disorders. Current opinion in neurology 2008;21:478-483.

19. Ruscher K, Kuric E, Wieloch T. Levodopa treatment improves functional recovery after experimental stroke. Stroke; a journal of cerebral circulation 2012;43:507-513.

20. Miyazaki I, Asanuma M, Diaz-Corrales FJ, Miyoshi K, Ogawa N. Direct evidence for expression of dopamine receptors in astrocytes from basal ganglia. Brain Res 2004;1029:120-123.

21. Ohta K, Kuno S, Mizuta I, Fujinami A, Matsui H, Ohta M. Effects of dopamine agonists bromocriptine, pergolide, cabergoline, and SKF-38393 on GDNF, NGF, and BDNF synthesis in cultured mouse astrocytes. Life Sci 2003;73:617-626.

22. Sarkar C, Basu B, Chakroborty D, Dasgupta PS, Basu S. The immunoregulatory role of dopamine: an update. Brain Behav Immun 2010;24:525-528.

23. Cepeda C, Colwell CS, Itri JN, Gruen E, Levine MS. Dopaminergic modulation of early signs of excitotoxicity in visualized rat neostriatal neurons. Eur J Neurosci 1998;10:3491-3497.

24. Biegon A, Fry PA, Paden CM, Alexandrovich A, Tsenter J, Shohami E. Dynamic changes in N-

methyl-D-aspartate receptors after closed head injury in mice: Implications for treatment of neurological and cognitive deficits. Proceedings of the National Academy of Sciences of the United States of America 2004;101:5117-5122.

25. Hosp JA, Pekanovic A, Rioult-Pedotti MS, Luft AR. Dopaminergic projections from midbrain to primary motor cortex mediate motor skill learning. J Neurosci 2011;31:2481-2487.

26. Scheidtmann K, Fries W, Muller F, Koenig E. Effect of levodopa in combination with physiotherapy on functional motor recovery after stroke: a prospective, randomised, double-blind study. Lancet 2001;358:787-790.

27. Lokk J, Salman Roghani R, Delbari A. Effect of methylphenidate and/or levodopa coupled with physiotherapy on functional and motor recovery after stroke--a randomized, double-blind, placebo-controlled trial. Acta neurologica Scandinavica 2011;123:266-273.

28. Sonde L, Lokk J. Effects of amphetamine and/or L-dopa and physiotherapy after stroke - a blinded randomized study. Acta neurologica Scandinavica 2007;115:55-59.

29. Seniow J, Litwin M, Litwin T, Lesniak M, Czlonkowska A. New approach to the rehabilitation of post-stroke focal cognitive syndrome: effect of levodopa combined with speech and language therapy on functional recovery from aphasia. Journal of the neurological sciences 2009;283:214-218.

30. Ford GA, Bhakta BB, Cozens A, et al. Safety and efficacy of co-careldopa as an add-on therapy to occupational and physical therapy in patients after stroke (DARS): a randomised, double-blind, placebo-controlled trial. Lancet neurology 2019;18:530-538.

31. Paolucci S, Antonucci G, Grasso MG, et al. Functional outcome of ischemic and hemorrhagic stroke patients after inpatient rehabilitation: a matched comparison. Stroke; a journal of cerebral circulation 2003;34:2861-2865.

32. Ford GA, Bhakta BB, Cozens A, et al. DARS (Dopamine Augmented Rehabilitation in Stroke): Longer-term results for a randomised controlled trial of Co-careldopa in addition to routine ocupational and physical therapy after stroke International Journal of Stroke 2015;10:5-13.

33. Delbari A, Salman-Roghani R, Lokk J. Effect of methylphenidate and/or levodopa combined with physiotherapy on mood and cognition after stroke: a randomized, double-blind, placebo-controlled trial. European neurology 2011;66:7-13.

34. Bernhardt J, Hayward KS, Kwakkel G, et al. Agreed definitions and a shared vision for new standards in stroke recovery research: The Stroke Recovery and Rehabilitation Roundtable taskforce. International journal of stroke : official journal of the International Stroke Society 2017;12:444-450.

35. Kwakkel G, Lannin NA, Borschmann K, et al. Standardized measurement of sensorimotor recovery in stroke trials: Consensus-based core recommendations from the Stroke Recovery and Rehabilitation Roundtable. International journal of stroke : official journal of the International Stroke Society 2017;12:451-461.

36. Engelter ST, Frank M, Lyrer PA, Conzelmann M. Safety of pharmacological augmentation of stroke rehabilitation. European neurology 2010;64:325-330.

37. Reinholz J, Skopp O, Breitenstein C, Winterhoff H, Knecht S. Better than normal: improved formation of long-term spatial memory in healthy rats treated with levodopa. Exp Brain Res 2009;192:745-749.

38. Knecht S, Breitenstein C, Bushuven S, et al. Levodopa: faster and better word learning in normal humans. Ann Neurol 2004;56:20-26.

39. Berends HI, Nijlant JM, Movig KL, Van Putten MJ, Jannink MJ, Ijzerman MJ. The clinical use of drugs influencing neurotransmitters in the brain to promote motor recovery after stroke; a Cochrane systematic review. Eur J Phys Rehabil Med 2009;45:621-630.

40. Yeo SH, Lim ZI, Mao J, Yau WP. Effects of Central Nervous System Drugs on Recovery After Stroke: A Systematic Review and Meta-Analysis of Randomized Controlled Trials. Clin Drug Investig 2017.

41. Çelik C, Uzun M, Karaoğlan B. Inmeli Hastalarda Rehabilitasyon Programi ile Birlikte Levodopa Tedavisinin Fonksionel Motor Iyileşme Üzerine Etkisi. Türkiye Fiziksel Tip ve Rehabilitasyon Dergisi 2004;5:18-20.

42. Acler M, Fiaschi A, Manganotti P. Long-term levodopa administration in chronic stroke patients. A clinical and neurophysiologic single-blind placebo-controlled cross-over pilot study. Restor Neurol Neurosci 2009;27:277-283.

43. Bhakta BB, Hartley S, Holloway I, et al. The DARS (Dopamine Augmented Rehabilitation in

Stroke) trial: protocol for a randomised controlled trial of Co-careldopa treatment in addition to routine NHS occupational and physical therapy after stroke. Trials 2014;15:316.

44. https://compendium.ch/prod/sinemet-tabl-25mg-100mg/de.

45. Monte-Silva K, Liebetanz D, Grundey J, Paulus W, Nitsche MA. Dosage-dependent non-linear effect of L-dopa on human motor cortex plasticity. J Physiol 2010;588:3415-3424.

46. Thirugnanasambandam N, Grundey J, Paulus W, Nitsche MA. Dose-dependent nonlinear effect of L-DOPA on paired associative stimulation-induced neuroplasticity in humans. J Neurosci 2011;31:5294-5299.

47. Weis T, Puschmann S, Brechmann A, Thiel CM. Effects of L-dopa during auditory instrumental learning in humans. PloS one 2012;7:e52504.

48. Fugl-Meyer AR, Jaasko L, Leyman I, Olsson S, Steglind S. The post-stroke hemiplegic patient. 1. a method for evaluation of physical performance. Scand J Rehabil Med 1975;7:13-31.

49. Sullivan KJ, Tilson JK, Cen SY, et al. Fugl-Meyer assessment of sensorimotor function after stroke: standardized training procedure for clinical practice and clinical trials. Stroke; a journal of cerebral circulation 2011;42:427-432.

50. Salter K, Campbell N, Richardson M, et al. Outcome Measures in Stroke Rehabilitation EBRSR Evidence-Based Review of Stroke Rehabilitation wwwebrsrcom 2013.

51. Miller EL, Murray L, Richards L, et al. Comprehensive overview of nursing and interdisciplinary rehabilitation care of the stroke patient: a scientific statement from the American Heart Association. Stroke; a journal of cerebral circulation 2010;41:2402-2448.

52. Zipp GP, Sullivan JE, Rose D, et al. Neurology Section. StrokeEDGE Taskforce. . 2011.

53. Page SJ, Fulk GD, Boyne P. Clinically important differences for the upper-extremity Fugl-Meyer Scale in people with minimal to moderate impairment due to chronic stroke. Phys Ther 2012;92:791-798.

54. Pandian S, Arya KN, Kumar D. Minimal clinically important difference of the lower-extremity fugl-meyer assessment in chronic-stroke. Top Stroke Rehabil 2016;23:233-239.

55. Lyden P, Brott T, Tilley B, et al. Improved reliability of the NIH Stroke Scale using video training. NINDS TPA Stroke Study Group. Stroke; a journal of cerebral circulation 1994;25:2220-2226.

56. van Swieten JC, Koudstaal PJ, Visser MC, Schouten HJ, van Gijn J. Interobserver agreement for the assessment of handicap in stroke patients. Stroke; a journal of cerebral circulation 1988;19:604-607.

57. Bruno A, Shah N, Lin C, et al. Improving modified Rankin Scale assessment with a simplified questionnaire. Stroke; a journal of cerebral circulation 2010;41:1048-1050.

58. Katzan IL, Thompson N, Uchino K. Innovations in Stroke: The Use of PROMIS and NeuroQoL Scales in Clinical Stroke Trials. Stroke; a journal of cerebral circulation 2016;47:e27-30.

59. Katzan IL, Fan Y, Uchino K, Griffith SD. The PROMIS physical function scale: A promising scale for use in patients with ischemic stroke. Neurology 2016;86:1801-1807.

60. Salinas J, Sprinkhuizen SM, Ackerson T, et al. An International Standard Set of Patient-Centered Outcome Measures After Stroke. Stroke; a journal of cerebral circulation 2016;47:180-186.

61. Hays RD, Spritzer KL, Schalet BD, Cella D. PROMIS((R))-29 v2.0 profile physical and mental health summary scores. Qual Life Res 2018;27:1885-1891.

62. Katzan IL, Lapin B. PROMIS GH (Patient-Reported Outcomes Measurement Information System Global Health) Scale in Stroke: A Validation Study. Stroke; a journal of cerebral circulation 2018;49:147-154.

63. Chen HM, Hsieh CL, Sing Kai L, Liaw LJ, Chen SM, Lin JH. The test-retest reliability of 2 mobility performance tests in patients with chronic stroke. Neurorehabil Neural Repair 2007;21:347-352.

64. Collin C, Wade D. Assessing motor impairment after stroke: a pilot reliability study. Journal of neurology, neurosurgery, and psychiatry 1990;53:576-579.

65. Lyle RC. A performance test for assessment of upper limb function in physical rehabilitation treatment and research. Int J Rehabil Res 1981;4:483-492.

66. Platz T, Pinkowski C, van Wijck F, Kim IH, di Bella P, Johnson G. Reliability and validity of arm function assessment with standardized guidelines for the Fugl-Meyer Test, Action Research Arm Test and Box and Block Test: a multicentre study. Clin Rehabil 2005;19:404-411.

67. Nijland R, van Wegen E, Verbunt J, van Wijk R, van Kordelaar J, Kwakkel G. A comparison of two validated tests for upper limb function after stroke: The Wolf Motor Function Test and the Action Research Arm Test. J Rehabil Med 2010;42:694-696.

68. Van der Lee JH, De Groot V, Beckerman H, Wagenaar RC, Lankhorst GJ, Bouter LM. The intra- and interrater reliability of the action research arm test: a practical test of upper extremity function in patients with stroke. Arch Phys Med Rehabil 2001;82:14-19.

69. Mathiowetz V, Volland G, Kashman N, Weber K. Adult norms for the Box and Block Test of manual dexterity. Am J Occup Ther 1985;39:386-391.

70. Collen FM, Wade DT, Bradshaw CM. Mobility after stroke: reliability of measures of impairment and disability. Int Disabil Stud 1990;12:6-9.

71. Holden MK, Gill KM, Magliozzi MR. Gait assessment for neurologically impaired patients. Standards for outcome assessment. Phys Ther 1986;66:1530-1539.

72. Holden MK, Gill KM, Magliozzi MR, Nathan J, Piehl-Baker L. Clinical gait assessment in the neurologically impaired. Reliability and meaningfulness. Phys Ther 1984;64:35-40.

73. Wade DT. Measurement in neurological rehabilitation. Curr Opin Neurol Neurosurg 1992;5:682-686.

74. Yi Y, Shim JS, Oh BM, Seo HG. Grip Strength on the Unaffected Side as an Independent Predictor of Functional Improvement After Stroke. Am J Phys Med Rehabil 2017;96:616-620.

75. Nasreddine ZS, Phillips NA, Bedirian V, et al. The Montreal Cognitive Assessment, MoCA: a brief screening tool for mild cognitive impairment. J Am Geriatr Soc 2005;53:695-699.

76. Leuenberger K, Gonzenbach R, Wachter S, Luft A, Gassert R. A method to qualitatively assess arm use in stroke survivors in the home environment. Med Biol Eng Comput 2017;55:141-150.

77. USE ICOHOTRFROPFH. Clinical Safety Data Management: Definitions and Standards for Expedited Reporting E2A. 1994.

78. International Conference on Harmonisation of Technical Requirements for Registration of Pharmaceuticals for Human Use. ICH Harmonised Tripartite Guideline. Clinical Safety Data Management: Definitions and Standards for Expedited Reporting E2A. . 1994.

79. Van Buuren S, Groothuis-Oudshoorn K. mice: Multivariate Imputation by Chained Equations in R. Journal of Statistical Software 2011;45.

17. APPENDICES

Appendix I: List of local principal investigators at all centers anticipated to participate in ESTREL.

Appendix II: List of members of the TSC.

Appendix III: Summary of product characteristics (IMP, Levodopa/Carbidopa).

Appendix IV: Summary of existing studies comparing Levodopa to control in stroke patients.

Appendix V: Genetic and biomarker analyses
