# Supplementary material for: ESTREL-Fatigue—association of levodopa with post-stroke fatigue
Source: Eur Stroke J. 2026 Apr 7;11(4):aakag029. doi: 10.1093/esj/aakag029 (PMC13131240; doi:10.1093/esj/aakag029)

# Supplementary Materials

**Table 1. Comparison between all participants of ESTREL and included and excluded participants of ESTREL-Fatigue**

|  | **Overall** | **Excluded** | **Included** | **p** |
| --- | --- | --- | --- | --- |
| **n** | 610 | 154 | 456 |  |
| **Age (median [IQR])** | 73.00 [64.00, 82.00] | 74.00 [64.00, 82.00] | 73.00 [63.00, 82.00] | 0.289 |
| **Sex = Female (%)** | 252 (41.3) | 66 (42.9) | 186 (40.8) | 0.722 |
| **mRS = mean [SD]** | 4.09 (0.94) | 4.18 (0.89) | 4.06 (0.96) | 0.180 |
| **FMMA = mean [SD]** | 36.03 (23.56) | 33.81 (24.59) | 36.78 (23.18) | 0.179 |
| **NIHSS = mean [SD]** | 8.23 (3.87) | 8.77 (4.18) | 8.05 (3.74) | 0.044 |
| **Aphasia = Yes (%)** | 101 (16.6) | 30 (19.5) | 71 (15.6) | 0.316 |

**Table 2. Comparison of medication adherence between the Levodopa and Placebo group**

First comparison between the levodopa and placebo allocation shows all 610 ESTREL participants. The second comparison shows 590 participants after the exclusion of death/withdrawal and before the exclusion due to non-adherence.

| **Medication** | **all (n)** | **adherent (n)** | **non adherent (n)** | **adherent (%)** | **non adherent (%)** | **p** |
| --- | --- | --- | --- | --- | --- | --- |
| ESTREL total  n = 610 |  |  |  |  |  |  |
| **Levodopa** | 307 | 256 | 51 | 83.4 | 16.6 | 0.7697 |
| **Placebo** | 303 | 249 | 54 | 82.2 | 17.8 |  |
|  |  |  |  |  |  |  |
| ESTREL total excl. death/withdrawal  n = 590 |  |  |  |  |  |  |
| **Levodopa** | 299 | 256 | 43 | 85.6 | 14.4 | 0.9136 |
| **Placebo** | 291 | 249 | 42 | 85.6 | 14.4 |  |

**Table 3. Comparison of medication adherence between participants with Fatigue and without Fatigue**

First comparison includes all 610 ESTREL participants and examines the association between adherence and fatigue. The second comparison includes the 85 participants excluded in ESTREL-Fatigue due to non-adherence and reports the proportion of fatigue (binomial test).

| ESTREL total  n = 610 | **missing data (n)** | **no Fatigue (n)** | **Fatigue (n)** | **no Fatigue (%)** | **Fatigue (%)** | **p** |
| --- | --- | --- | --- | --- | --- | --- |
| **adherent** | 49 | 324 | 132 | 71,1 | 28,9 | 0,1769 |
| **non-adherent** | 51 | 33 | 21 | 61,1 | 38,9 |  |
|  |  |  |  |  |  |  |
| **Excluded from ESTREL-Fatigue**  **n = 85** | **missing data (n)** | **no Fatigue (n)** | **Fatigue (n)** | **no Fatigue (%)** | **Fatigue (%)** | **p** |
| **non-adherent** | 31 | 33 | 21 | 61.1 | 38.9 | 0.1337 |

**Figure 1. Fatigue items of the PROMIS Fatigue Shortform-4a within the PROMIS-29 questionnaire**


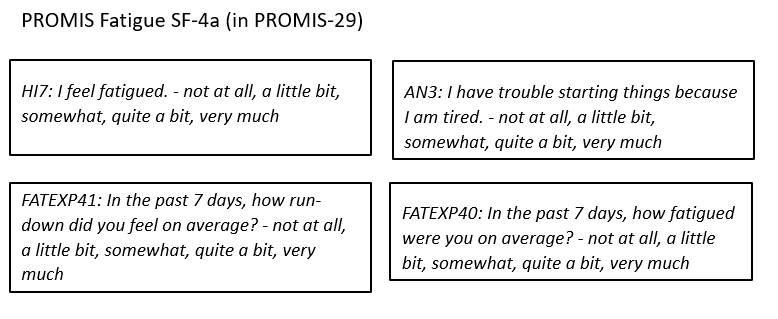


**Figure 2. Fatigue item within the PROMIS-10 questionnaire**


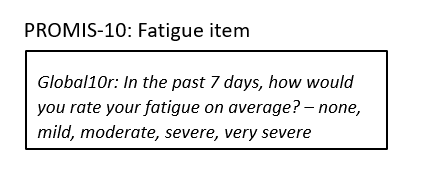

Supplement: aakag029_Supplemental_Files [file aakag029_supplemental_files.zip › ESTREL-Fatigue Supplementary Materials.docx]
