# Supplementary material for: ESTREL-Fatigue—association of levodopa with post-stroke fatigue
Source: Eur Stroke J. 2026 Apr 7;11(4):aakag029. doi: 10.1093/esj/aakag029 (PMC13131240; doi:10.1093/esj/aakag029)
Supplement: aakag029_Supplemental_Files [file aakag029_supplemental_files.zip › SAP_ESTREL_prod_1761597796.4701.pdf]

Universität Basel
Departement Klinische Forschung

Universitätsspital Basel

Universität Basel, Departement Klinische Forschung, c/o Universitätsspital Basel, Spitalstrasse 12, 4031 Basel

Prof. Dr. Stefan Engelter
Felix-Platter Spital Basel
Burgfelderstrasse 101
4002 Basel

Final Report

Basel, September 27, 2024

Methods & Statistics

Study Title	Enhancement of Stroke Rehabilitation with Levodopa
Internal DKF ID	me16Engelter
Document Status	Final Report

1 Background

In stroke medicine, the large body of high-quality evidence proving benefits of acute revascularization therapies and secondary prevention is offset by a large gap of evidence on means to enhance stroke recovery. Dopamine is a key player in processes of motor learning, reward, and brain plasticity. Levodopa - a precursor of dopamine - is a promising candidate for the pharmacological enhancement of stroke recovery. Preclinical research and studies with healthy individuals suggest, that there is scope for benefit from applying Levodopa in addition to standardized rehabilitation. Indeed, there are some, however limited and inconsistent data from small randomized controlled trials (RCTs) testing Levodopa in stroke patients. A meta-analysis across 6 RCTs indicated the possibility of a more favorable outcome in Levodopa-treated stroke patients than in control patients. Heterogeneity between trials was considerable. The RCTs differed regarding patient populations (chronic/acute stroke), types of stroke (ischemic/hemorrhagic), dosage and duration of the study treatment, length of follow-up, and outcome measures. None mentioned adaptation of concomitant rehabilitation therapy to the principles of motor learning. Of note, safety concerns were absent. Motor deficits are common and affect quality of life in stroke patients prompting for motor improvement as a top priority. Given the high prevalence and tremendous burden of stroke, a straightforward applicable measure to improve motor outcome is highly relevant. Given the promising but inconclusive clinical trial evidence on benefits, a well-designed, randomized controlled trial studying the usefulness of Levodopa in enhancing motor recovery after stroke is warranted.

Universität Basel
Departement Klinische Forschung
c/o Universitätsspital Basel
Spitalstrasse 12
4031 Basel, Switzerland

Sabine Schädelin, MSc
Statistician, Department of Clinical Research
Telefon +41 61 556 51 67
sabine.schaedelin@usb.ch
dkf.unibas.ch

Final Report

2 Objectives of the analysis

In this study we investigate whether Levodopa/Carbidopa 100/25mg three times daily compared to placebo given in addition to standardized rehabilitation is associated with a patient-relevant enhancement of functional recovery in acute ischemic or hemorrhagic stroke patients.

2.1 Primary objective

In **patients with acute ischemic stroke or hemorrhagic stroke** we investigate if treatment with **Levodopa** in combination with standardized rehabilitation as compared to **placebo** in combination with standardized rehabilitation improves functional recovery after three months as quantified using the **Fugl-Meyer Motor Assessment (FMMA)**.

2.2 Secondary objectives

The secondary objective is to investigate potential effects of Levodopa on the survival and general health of the patients.

Final Report

3 Study design, population and analysis sets

3.1 Study design

This study is a randomized multicenter superiority trial.

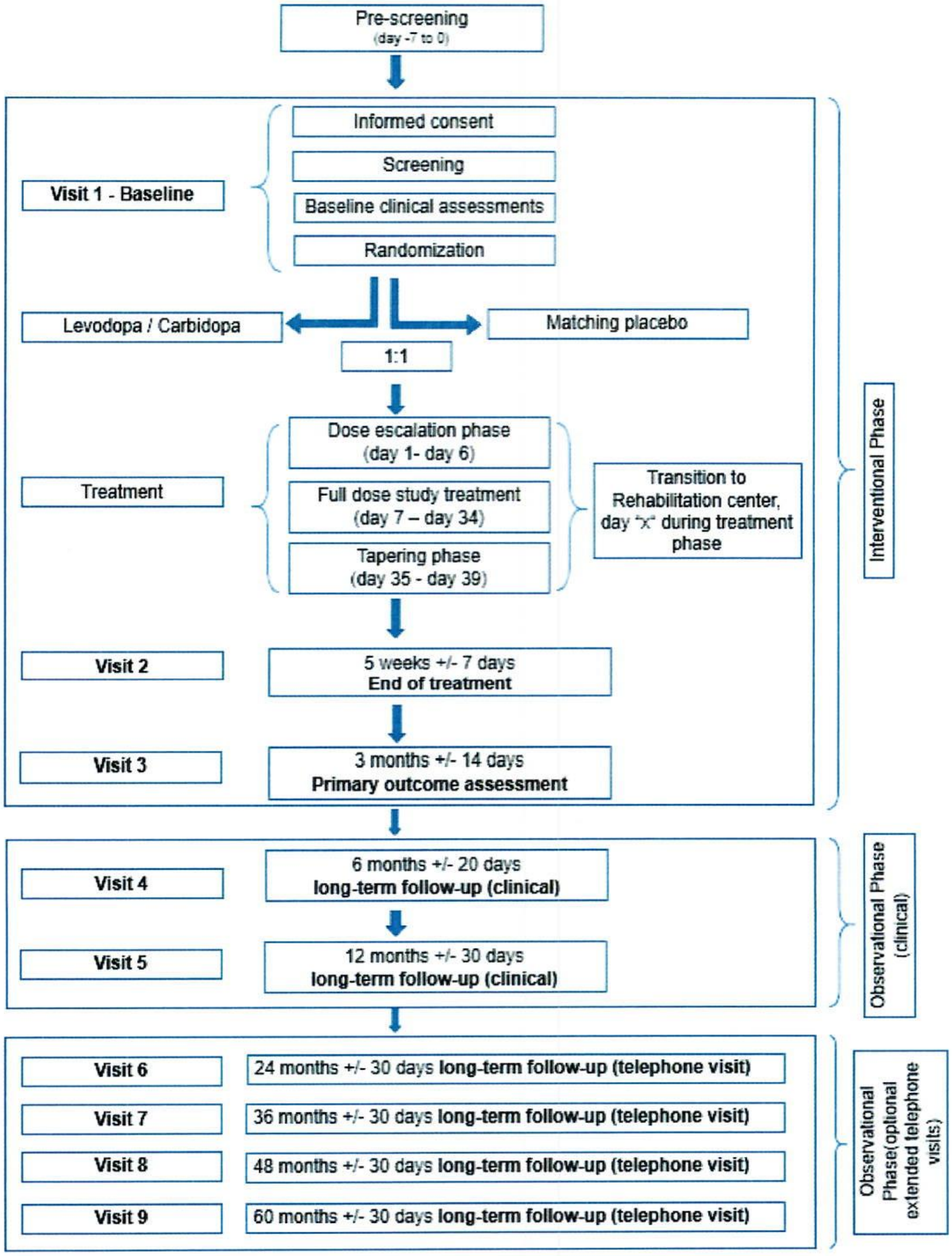

Figure 1: Study Flow Chart

3.2 Population and Analysis sets

The **full analysis set (FAS)** consists of all patients that were randomised. We will use multiple imputations based on chained equations to impute missing FMMA in patients who survived up to Visit 3 (see section 6 for details). Patients who died before Visit 3 will not be imputed except for the analysis Treatment policy strategy III[1].

The **modified intention to treat set (mITT)** consists of all patients in the FAS with a complete FMMA at Visit 3.

3.3 Inclusion criteria

- Acute ischemic or hemorrhagic (i.e. intracerebral hemorrhage excluding subarachnoid hemorrhage and cerebal venous sinus thrombosis) stroke $\leq$ 7 days prior to randomization
- Clinically meaningful hemiparesis (i.e. scoring a total of $\geq$ 3 points on the following NIH stroke scale score items (i) motor arm, (ii) motor leg, (iii) limb ataxia; a distal arm paresis is equivalent to one of the aforementioned (i-iii)).
- Time of randomization $\geq$ 24 hours since thrombolysis or thrombectomy.
- In-hospital rehabilitation required
- Capable to participate in standardized rehabilitation therapy
- Informed consent of patient or next of kin

3.4 Exclusion criteria

- Age $<$ 18 years
- Diagnosis of Parkinsons Disease
- Use of Levodopa mandatory according to judgement of treating physician
- Inability or unwillingness to comply with study procedures including adherence to study drug intake (orally, or via nasogastric tube or percutaneous endoscopic gastrostomy tube)
- Severe aphasia (i.e. unable to follow two-stage-commands)
- Previously dependent in the basal activities of daily living (defined as modified Ranking Scale prior to stroke $>$ 3)
- Pre-existing hemiparesis
- Known hypersensitivity to Levodopa/Carbidopa and other contraindications for Levodopa/Carbidopa as outlined in the summary of product characteristics (as appended to the study protocol)

[1] Patients who died before Visit 3 are part of the FAS but will not contribute to any analysis but the Treatment policy strategy III and the Composite strategy.

Final Report

- Women who are pregnant or breast feeding, or who intend to become pregnant during the course of the study. Women of childbearing age must take a pregnancy test to be eligible for the study.
- Lack of safe contraception, defined as: Female Participants of childbearing potential, not using and not willing to continue using a medically reliable method of contraception for the entire study duration, such as oral, injectable, or implantable contraceptives, or intrauterine contraceptive devices, or who are not using any other method considered sufficiently reliable by the Investigator in individual cases. Female Participants who are surgically sterilized / hysterectomized or post-menopausal for longer than 2 years are not considered as being of child-bearing potential.

Final Report

4 Outcomes

4.1 Primary outcome

The primary efficacy outcome is the final score in the Fugl-Meyer Motor Assessment (FMMA) measured at Visit 3.

The FMMA is a stroke-specific impairment index designed to assess motor recovery. Scale items are scored on the basis of ability to complete the item using a 3-point ordinal scale (0=cannot perform; 1=performs partially and 2=performs fully). FMMA total scores range from 0 (no movements) to 100 (normal movements) with 66 points for movements of the upper limbs (FMMA-UE) and 34 for those of the lower limbs (FMMA-LE). All assessments are done by a trained outcome assessor.

4.2 Secondary outcomes

The following secondary outcomes will be analysed at the end of treatment (Visit 2), at end of the interventional study phase (Visit 3), and in the long-term follow up (Visit 4 and Visit 5).

FMMA:
- FMMA total score additionally at Visit 2, Visit 4, and Visit 5 months[2]
- FMMA upper extremity and lower extremity score (separately)
- FMMA of the affected side

Stroke Severity: NIH-Stroke Scale Score

Disability and dependence in daily activities: Modified Rankin Scale (mRS)

Patient-reported outcome: Patient Reported Outcome Measure Information System (PROMIS)

1. PROMIS-29
2. PROMIS 10 (10 Question short form)

Patient-reported assessment of relevance of motor improvement

[2] FMMA total score at Visit 3 serves as primary endpoint.

Mobility: Rivermead Mobility Index - In order to allow comparison with the DARS study, this endpoint will be analysed as a continuous variable as well as binary variable "ability to walk independently" defined by a score of 7 or above.

Potential harm: Serious adverse events and non serious adverse events are measured up to Visit 3, mortality and recurrent stroke until Visit 5. These endpoints of potential harm are summarized as follows:

The following endpoints will be summarized up to Visit 5:

1. Mortality (all cause)
2. Recurrent stroke

The following endpoints will be summarized up to Visit 3:

1. Serious adverse events
2. Non-serious adverse events possibly related to the IMP

4.3 Estimands

An estimand defines in detail what needs to be estimated to address a specific scientific question of interest. A description of an estimand includes five attributes: A) the treatment, B) the population, C) the endpoint, D) the population-level summary and E) the handling of intercurrent events. Each estimand handles intercurrent events in a different way and thus answers a different study question.

We assume the following intercurrent events that potentially impact our outcome assessment:

(a) Death prior to Visit 3

(b) Serious adverse event leading to a motor impairing event prior to Visit 3 (e.g., restrokes, fractures, comparable complications)

(c) Intake of less than 80% of the study medication

(d) At least one week with fewer than five rehabilitation sessions per week during a period of 4 weeks.

(e) Visit 3 was out of window

The primary analysis will be the **treatment policy strategy**. The other estimands help to better understand the data and should be interpreted as supportive analyses only.

4.3.1 Primary estimand: treatment policy strategy

For the primary analysis we will use a **treatment policy strategy**. To this end attributes A) - E) are defined as follows:

A) the treatment Levodopa in combination with standardized rehabilitation vs placebo in combination with standardized rehabilitation,

Final Report

B) the population FAS as defined in section 3.2,

C) the endpoint Fugl-Meyer Motor Assessment (FMMA) measured at Visit 3,

D) the population-level summary mean difference between the two treatment arms,

E) the handling of intercurrent events According to the intention to treat principle all patients will be analyzed according to randomization irrespective of the presence or absence of intercurrent events defined in section 4.3.

To address the intercurrent events listed in section 4.3, we defined the following further estimands. Deviations from the primary estimand are highlighted in blue:

Final Report

4.3.2 Treatment policy strategy II, complete case analysis

To assess the effect of missing FMMA we will use a **Treatment policy strategy II, complete case analysis** defining A) - E) as follows:

A) the treatment Levodopa in combination with standardized rehabilitation vs placebo in combination with standardized rehabilitation,

B) the population mITT set: as defined in section 3.2,

C) the endpoint Fugl-Meyer Motor Assessment (FMMA) measured at Visit 3,

D) the population-level summary mean difference between the two treatment arms,

E) the handling of intercurrent events According to the intention to treat principle all patients will be analyzed according to randomization irrespective of the presence or absence of intercurrent events defined in section 4.3.

4.3.3 Treatment policy strategy III, FAS including patients who died

To assess the effect of missing FMMA due to death before Visit 3 we will use a **Treatment policy strategy III, FAS including patients who died** defining A) - E) as follows:

A) the treatment Levodopa in combination with standardized rehabilitation vs placebo in combination with standardized rehabilitation,

B) the population FAS set: as defined in section 3.2

C) the endpoint Fugl-Meyer Motor Assessment (FMMA) measured at Visit 3,

D) the population-level summary mean difference between the two treatment arms,

E) the handling of intercurrent events According to the intention to treat principle all patients will be analyzed according to randomization irrespective of the presence or absence of intercurrent events defined in section 4.3. In this analysis FMMA of patients who died will be imputed as well.

4.3.4 Composite strategy using win-ratios

To assess the effect of death we will use a **Composite strategy using win-ratios** defining A) - E) are defined as follows:

A) the treatment Levodopa in combination with standardized rehabilitation vs placebo in combination with standardized rehabilitation,

B) the population FAS as defined in section 3.2[3],

C) the endpoint Death and Fugl-Meyer Motor Assessment (FMMA) measured at Visit 3,

D) the population-level summary difference in win-ratio at Visit 3,

E) the handling of intercurrent events According to the intention to treat principle all patients will be analyzed according to randomization irrespective of the presence or absence of intercurrent events defined in section 4.3.

4.3.5 Hypothetical strategy

To assess the effect of a serious adverse event leading to a motor impairing event prior to Visit 3 (e.g., restrokes, fractures, comparable complications) between randomization and Visit 3 we will use a **Hypothetical strategy** defining A) - E) are defined as follows:

A) the treatment Levodopa in combination with standardized rehabilitation vs placebo in combination with standardized rehabilitation,

B) the population FAS as defined in section 3.2,

C) the endpoint Fugl-Meyer Motor Assessment (FMMA) measured at Visit 3,

D) the population-level summary mean difference between the two treatment arms,

E) the handling of intercurrent events According to the intention to treat principle all patients will be analyzed according to randomization excluding participants in whom the intercurrent event (b) described in section 4.3 occurred.

4.3.6 On treatment strategy

To assess the effect of low documented intake of study medication we will use the **While on treatment strategy** defining A) - E) are defined as follows:

A) the treatment Levodopa in combination with standardized rehabilitation vs placebo in combination with standardized rehabilitation,

B) the population FAS set: as defined in section 3.2,

C) the endpoint Fugl-Meyer Motor Assessment (FMMA) measured at Visit 3,

[3] In this analysis patients who died before Visit 3 are included as well. No FMMA is needed for these patients - death is considered as the worst possible outcome.

Final Report

D) the population-level summary mean difference between the two treatment arms,

E) the handling of intercurrent events According to the intention to treat principle all patients will be analyzed according to randomization excluding participants in whom the intercurrent event (c) described in section 4.3 occurred.

4.3.7 On treatment strategy II

To assess the effect of low amount of documented rehabilitation therapy we will use the **While on treatment strategy II** defining A) - E) are defined as follows:

A) the treatment Levodopa in combination with standardized rehabilitation vs placebo in combination with standardized rehabilitation,

B) the population FAS set: as defined in section 3.2,

C) the endpoint Fugl-Meyer Motor Assessment (FMMA) measured at Visit 3,

D) the population-level summary mean difference between the two treatment arms,

E) the handling of intercurrent events According to the intention to treat principle all patients will be analyzed according to randomization excluding participants in whom the intercurrent event (d) described in section 4.3 occurred.

4.3.8 Measured according to protocol

To assess the effect of outcome assessment outside the predefined window we use a **While measured according to protocol strategy** defining A) - E) are defined as follows:

A) the treatment Levodopa in combination with standardized rehabilitation vs placebo in combination with standardized rehabilitation,

B) the population FAS set: as defined in section 3.2,

C) the endpoint Fugl-Meyer Motor Assessment (FMMA) measured at Visit 3,

D) the population-level summary mean difference between the two treatment arms,

E) the handling of intercurrent events According to the intention to treat principle all patients will be analyzed according to randomization excluding participants in whom the intercurrent event (e) described in section 4.3 occurred.

4.4 Study sensitivity level

Given the vulnerability of this population, safety analyses (endpoints for potential harm) are of importance. Potential safety issues were mitigated by the fact that there is a DSMB which investigates safety independently of the results presented in this report.

The main result has the potential of altering the standard management of stroke patients and thus will be considered as a result of high impact.

Final Report

Final Report

5 Specific methods

5.1 Demographic and baseline characteristics

Demographics and relevant baseline variables will be summarized for the FAS and the mITT set[4]. The data will be stratified by treatment arm.

Categorical data will be presented as frequencies and percentages. For continuous variables the mean and the standard deviation will be presented. In case of non-normally distributed variables, the median and the interquartile range will be presented.

5.2 Analysis of the Primary Objective

Fugl-Meyer Motor Assessment at Visit 3 will be analyzed by a linear model, with Fugl-Meyer Motor Assessment at baseline as covariate to adjust for differences at baseline and treatment as two-level factor ("Levodopa" vs. "placebo") as variable of interest. The estimated treatment effects will be presented together with p-values and 95 % confidence intervals. This estimate can be interpreted as the mean difference[5] of the FMMA between patients treated with "Levodopa" vs. "placebo" at Visit 3.

The model assumptions will be tested by checking the model assumptions and by inspecting the residuals and leverages. In case of violation of the model assumptions, data transformation will be considered.

The analysis will be performed using $\alpha = 0.05$ (two-sided).

5.3 Secondary Analyses

All secondary outcomes except for the outcomes for potential harm (see below) will be analysed by regression models appropriate to the data type. The analyses will be adjusted for the baseline measures where available. Binary outcomes will be compared using a logistic regression (generalized linear model with logit link and binomial error distribution). For continuous outcomes, linear models will be used. Ordinal outcomes will be analysed using ordinal logistic regression.

The models will be inspected as described for the main analysis. In case of severe violation of the model assumptions or presence of outliers with high leverage, transformation of the data or the use of other methods such as non-parametric tests or robust regression models will be considered.

The outcomes for potential harm will be summarized by treatment arm.

Furthermore, all secondary outcomes will be presented descriptively in summary tables and graphs, possibly in separate publications if deemed useful.

For all secondary analyses the primary estimand will be used. However, for the outcome FMMA on the affected side, we will exclude patients with bilateral pathology.

[4] We will consider presenting additional baseline tables including only patients used in the model for a given estimand if the result from the estimand deviates from the main estimand.

[5] adjusted for FMMA at baseline. This estimate is equivalent with the difference in the change in FMMA between baseline and Visit 3 (when adjusted for FMMA at baseline).

Final Report

6 Handling missing data

The number of missing values will be presented for each variable.

We will use 100 multiple imputations using chained equations. The imputed values, that have been generated by the imputation model, will be displayed in plots. Thereafter the model will be fit on each imputed data set. Estimates and variances will be combined using Rubins rules to give a single estimate and variance for each parameter of interest across all imputations.

Patients with a missing value in a secondary outcome will be imputed as described for the primary analysis. Outcomes for potential harm will not be imputed.

7 Supplementary analyses

Any heterogeneity of treatment effects across centers will be investigated in an explorative manner. Thereby center as well as a term for treatment-by-centre interaction will be included in the main model. In addition, the heterogeneity will be investigated by graphical display of the results stratified by center, or groups of centers.

7.1 Analyses for secondary estimands

Treatment policy strategy II, complete case analysis: The same model will be fit as described for the primary estimand.

Treatment policy strategy III, complete case analysis: The same model will be fit as described for the primary estimand.

Composite variable strategy: Levodopa and placebo will be compared on the basis of the unmatched win-ratio approach of Pocock et al. (2012). In this approach a algorithm is pre-defined which is able to determine for each pair of patients, who is "better off" ie. the "winner".

More precise, every patient in the Leopoda group will be compared with every patient in the placebo group determining the winner as follows:

1. A patient who survives up to Visit 3 wins over a patient who dies before Visit 3
2. If both patients die before Visit 3, the patient who survives longer wins
3. If both patients survive up to end of study, the patient with the higher Fugl-Meyer Motor Assessment wins

The win ratio will be estimated by dividing the number of cases in which treatment wins with the number of cases in which placebo wins and will be presented together with its 95 % confidence interval (CI) estimated based on 1000 bootstrap samples as described in the statistical appendix of Pocock et al. (2012). The analysis will be repeated on each imputed data set. If the CI for the win ratio does not contain the value 1 we will consider this as evidence for differences between the two treatment arms and discuss it similar to a conventional sensitivity analyses with a p-value < 0.05[6].

[6] Since all analyses in this section are sensitivity analyses and no correction for multiple testing is performed. All results have to be discussed in the context of the main analysis and should not be used as stand-alone evidence.

Final Report

Hypothetical strategy: The same model will be fit as described for the primary estimand.

While on treatment strategy: The same model will be fit as described for the primary estimand.

While on treatment strategy II: The same model will be fit as described for the primary estimand.

While measured according to protocol strategy: The same model will be fit as described for the primary estimand.

7.2 Subgroup analyses

The underlying hypotheses of the subgroups are pre-specified with the ICEMAN instrument prior to the subgroup analyses and recorded in an addendum to the SAP at that time.

1. Age ("$<$ median" vs. "$\geq$ median")
2. Sex ("male" vs. "female")
3. Presence of selective hand and wrist movement at baseline (NIHSS, subscore *Distal Motor Function*: "0" vs. "$>$ 0")
4. Cortical dysfunction (sum of NIHSS, subscores *Best Language*, *Extinction and Inattention*, and *Visual* "0" vs. "$>$ 0")
5. Presence of Selective Serotonin Re-uptake Inhibitor (SSRI) treatment at baseline ("yes" vs. "no")
6. Stroke type ("Acute ischemic stroke" vs. "intracerebral hemorrhage").
7. Severity of FMMA at baseline

For each subgroup the primary estimand will be used. Subgroups will be analysed using the same model as described for the main analysis but including the variable of the subgroup and its interaction with treatment arm as additional fixed effect. The subgroup analyses will be presented in a forest plot.

All analyses will be performed in `R version 4.3.1` or higher .

Role: Sponsor-Investigator:
Name: Prof. Dr. med. Stefan Engelter

Place/Date: Basel 27.9. 2024 Signature: [signature]

Role: Trial Statistician
Name: Sabine Schädelin

Place/Date: Basel, 27.9.2024 Signature: [signature]

Final Report

References

Pocock, S. J.; Ariti, C. A.; Collier, T. J. & Wang, D. 2012: The win ratio: a new approach to the analysis of composite endpoints in clinical trials based on clinical priorities. European heart journal 33(2):176–182.

Addendum to the Methods & Statistics

Version: Final Report from 04.10.2024

Inhalt

Prespecified Hypotheses of Subgroups with ICEMAN 2

Prespecified Hypotheses of Biomarker Subgroups with ICEMAN 4

Prespecified Hypotheses of Genetic Subgroup Analyses with ICEMAN 5

Predefined Analyses 6

Prespecified Hypotheses of Subgroups with ICEMAN

Subgroups	Dichotomization	A priori hypothesized direction of the effect modification
Age	< median vs. ≥ median	Younger individuals typically exhibit greater neuroplasticity and learning capacity, which are critical for motor recovery following a stroke. Levodopa is known to enhance motor learning by modulating dopaminergic pathways. Given that dopamine plays a key role in both learning and motor control, we hypothesize that younger participants may experience a more pronounced enhancement of motor learning when treated with levodopa, compared to older participants. Therefore, might be an effect modifier in the relationship between levodopa and motor learning outcomes.
Sex	Male vs. female	We do not expect any difference in the direction or magnitude of the treatment effect of levodopa between sexes.
Presence of selective hand and wrist movement at baseline	NIHSS, subscore Distal Motor Function: 0 vs. > 0	We do not expect any difference in the direction or magnitude of the treatment effect of levodopa between participants with and without selective hand and wrist movement.
Cortical dysfunction	The sum of NIHSS, subscores; Best Language, Extinction and Inattention, and Visusl: 0 vs > 0	We do not expect any difference in the direction or magnitude of the treatment effect of levodopa between participants with and without cortical dysfunction, however, the cortical dysfunction could have an impact on the learning capacities and therefore lead to a worse stroke recovery.
Presence of Selective Serotonin Re-uptake Inhibitor (SSRI) treatment at baseline	Yes vs. no	We do not expect any difference in the direction or magnitude of the treatment effect of levodopa between participants with and without SSRI treatment at baseline.
Stroke type	Acute ischemic stroke vs. intracerebral hemorrhage	We do not expect any difference in the direction or magnitude of the treatment effect of levodopa between participants with acute ischemic stroke versus intracerebral hemorrhage.
Severity of FMMA at baseline	< median vs. ≥ median	We do not expect any difference in the direction or magnitude of the treatment effect of levodopa between participants with different severities of FMMA at baseline.

Prespecified Hypotheses of Biomarker Subgroups with ICEMAN

Biomarker	Dichotomization	A priori hypothesized direction of the effect modification
Neuroaxonal injury	Neurofilament light chain (NfL)	We do expect a difference in the direction or magnitude of stroke recovery based on the concentration of NfL. However, we do not anticipate any difference in the direction or magnitude of the treatment effect of levodopa between participants with high versus low concentrations of NfL.
	Tau	We do expect a difference in the direction or magnitude of stroke recovery based on the concentration of Tau. However, we do not anticipate any difference in the direction or magnitude of the treatment effect of levodopa between participants with high versus low concentrations of Tau.
Myocardial injury and dysfunction	Hs-troponinT	We do expect a difference in the direction or magnitude of stroke recovery based on the concentration of Hs-troponinT. However, we do not anticipate any difference in the direction or magnitude of the treatment effect of levodopa between participants with high versus low concentrations of Hs-troponinT.
	NT-pro-BNP	We do expect a difference in the direction or magnitude of stroke recovery based on the concentration of NT-pro-BNP. However, we do not anticipate any difference in the direction or magnitude of the treatment effect of levodopa between participants with high versus low concentrations of NT-pro-BNP.
Aging processes of the immune system	GDF-15	We do expect a difference in the direction or magnitude of stroke recovery based on the concentration of GDF-15. However, we do not anticipate any difference in the direction or magnitude of the treatment effect of levodopa between participants with high versus low concentrations of GDF-15.
	Senescence-associated secretory phenotype panel (IL-1b, IL-6, IL-10, MCP-1, TNFa, MMP-1, oestopontin)	We do expect a difference in the direction or magnitude of stroke recovery based on the Senescence-associated secretory phenotype panel. However, we do not anticipate any difference in the direction or magnitude

		of the treatment effect of levodopa between participants with high versus low concentrations in the Senescence-associated secretory phenotype panel.

Prespecified Hypotheses of Genetic Subgroup Analyses with ICEMAN

SNP	Dichotomization	A priori hypothesized direction of the effect modification
Rs1842681	Homozygous / heterozygous	We hypothesize that the risk allele of SNP rs1842681 might contribute to an unfavorable stroke recovery.
ApoE	Homozygous / heterozygous	We hypothesize that the risk allele of the ApoE might contribute to an unfavorable stroke recovery in patients suffering from a hemorrhagic stroke.
For all other planned genetic analyses (including levodopa score, copy number variation, and SNPs), the approach will be exploratory, as there is currently limited evidence on the role of genetics in stroke recovery.		

Predefined Analyses

Planned Analyses	Dependent on a positive result	A priori hypothesized direction of the effect modification
Comparison of ESTREL participants with patients in the Swiss Stroke Registry	NO	We will compare the outcomes of the ESTREL participants with those of similarly affected patients who did not participate in ESTREL from the National (i.e. Swiss) stroke Registry<br><br>We hypothesize that patients who participated in the ESTREL trial will exhibit better stroke recovery outcomes compared to those who did not participate.
Impact of type and amount of rehabilitation therapies on motor recovery – with and without levodopa	YES/NO	We will investigate (i) the impact of the type and amount of rehabilitation therapies on motor recovery and (ii) if the levodopa treatment effect depends on the type and amount of rehabilitation therapies.<br><br>We hypothesize that a greater amount of motor rehabilitative therapy based on the principles of motor learning will lead to better stroke recovery outcomes. Furthermore, we hypothesize that the treatment effect of levodopa will be influenced by the extent of motor rehabilitative therapy that incorporates these principles
Association of levodopa and post-stroke fatigue		We hypothesize that Levodopa, due to its motivational properties, may be associated with reduced post-stroke fatigue.
Association of levodopa and post-stroke depression		We hypothesize that Levodopa, due to its motivational properties, may be associated with reduced post-stroke depression.

Universitäre Altersmedizin FELIX PLATTER
Burgfelderstrasse 101 | 4055 Basel | Schweiz

Prof. Dr. med. Stefan Engelter
Chefarzt Rehabilitation, Neurologie & Neurorehabiliation
+41 61 326 41 30 | stefan.engelter@felixplatter.ch

Basel 4.10.2024
